# Supplementary figures and images for: Interplay between PML NBs and HIRA for H3.3 dynamics following type I interferon stimulus
Source: eLife. 2023 May 25;12:e80156. doi: 10.7554/eLife.80156 (PMC10212570; doi:10.7554/eLife.80156)

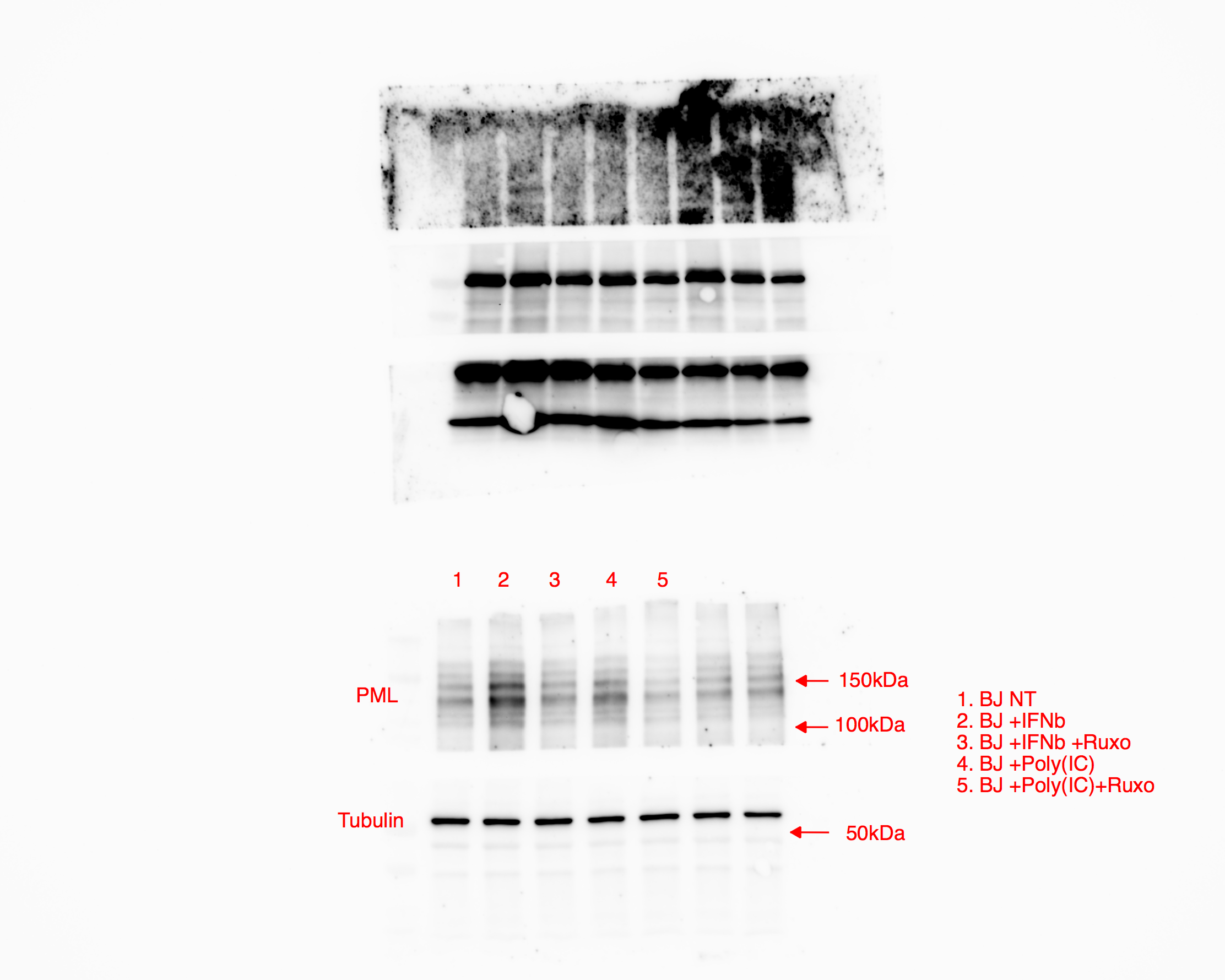

Supplement: Figure 1—source data 1. [file elife-80156-fig1-data1.tif]

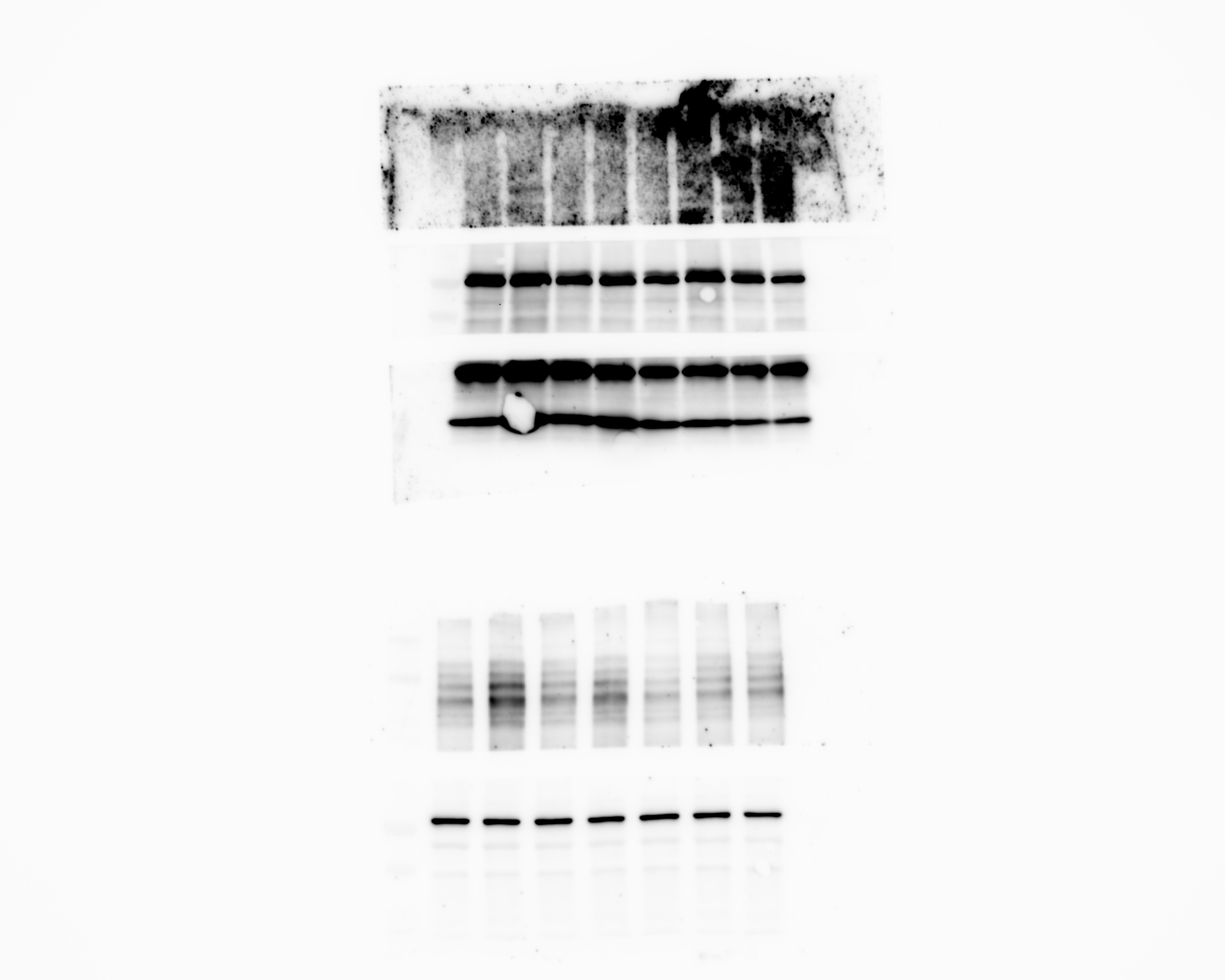

Supplement: Figure 1—source data 2. [file elife-80156-fig1-data2.tif]

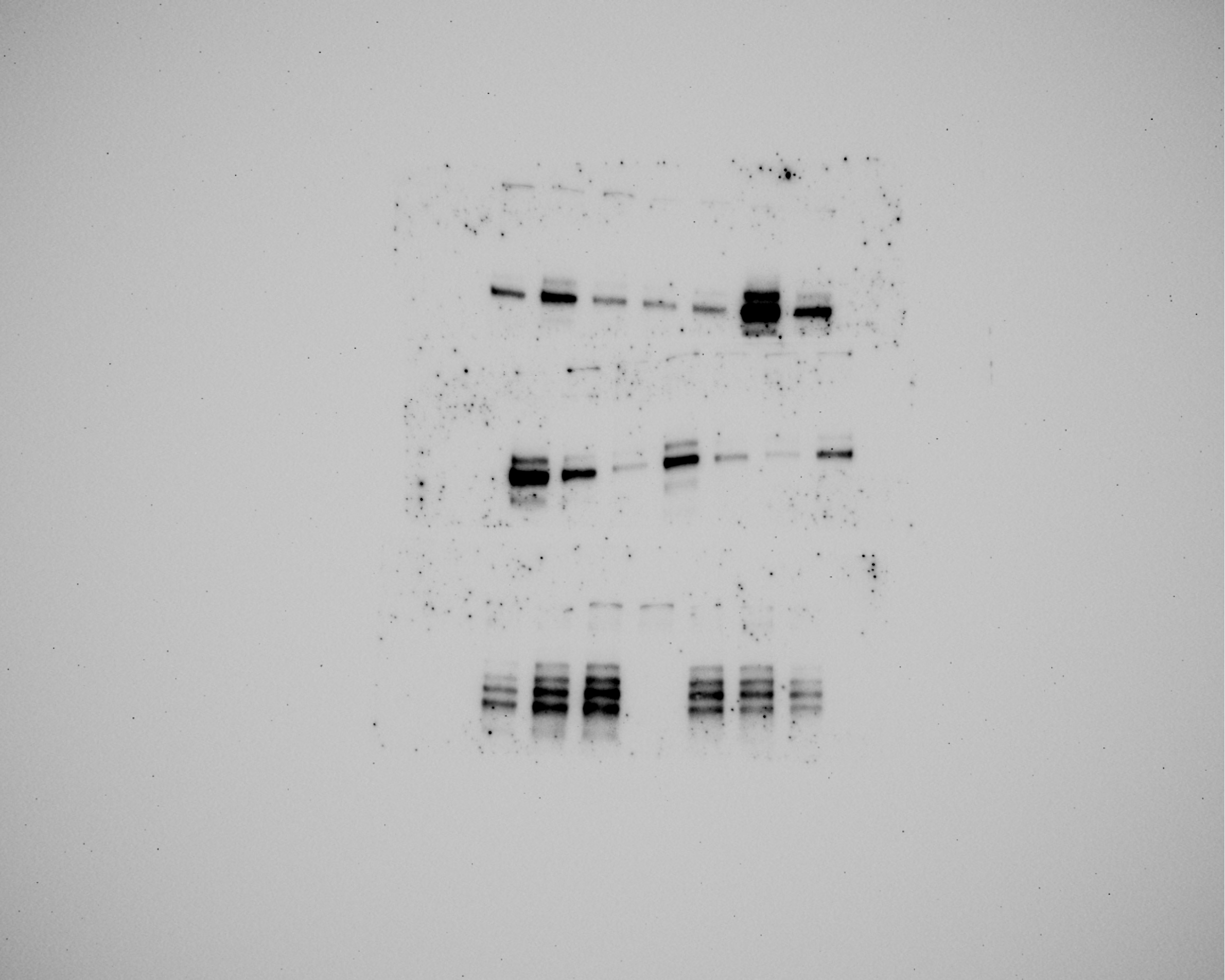

Supplement: Figure 1—source data 3. [file elife-80156-fig1-data3.tif]

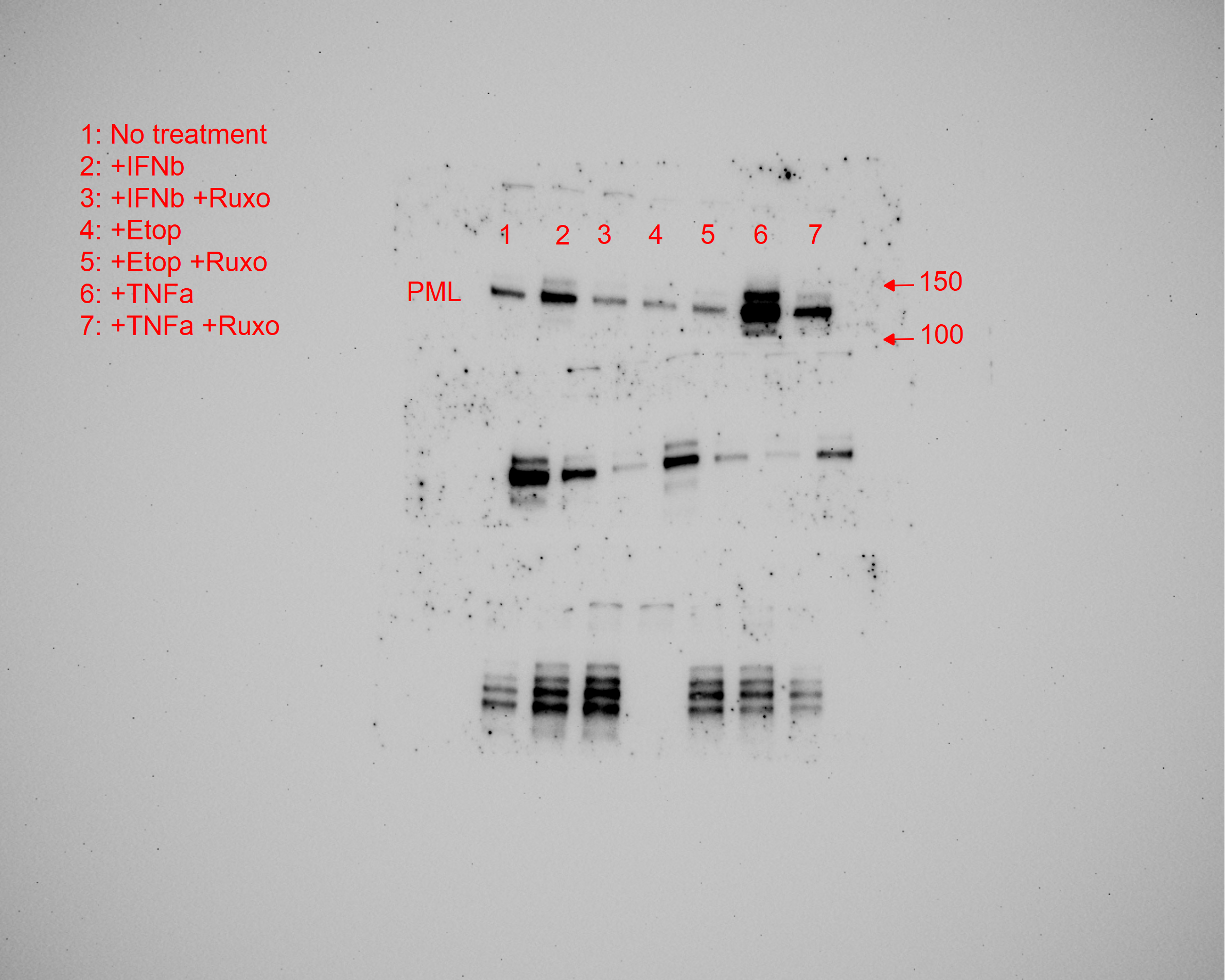

Supplement: Figure 1—source data 4. [file elife-80156-fig1-data4.tif]

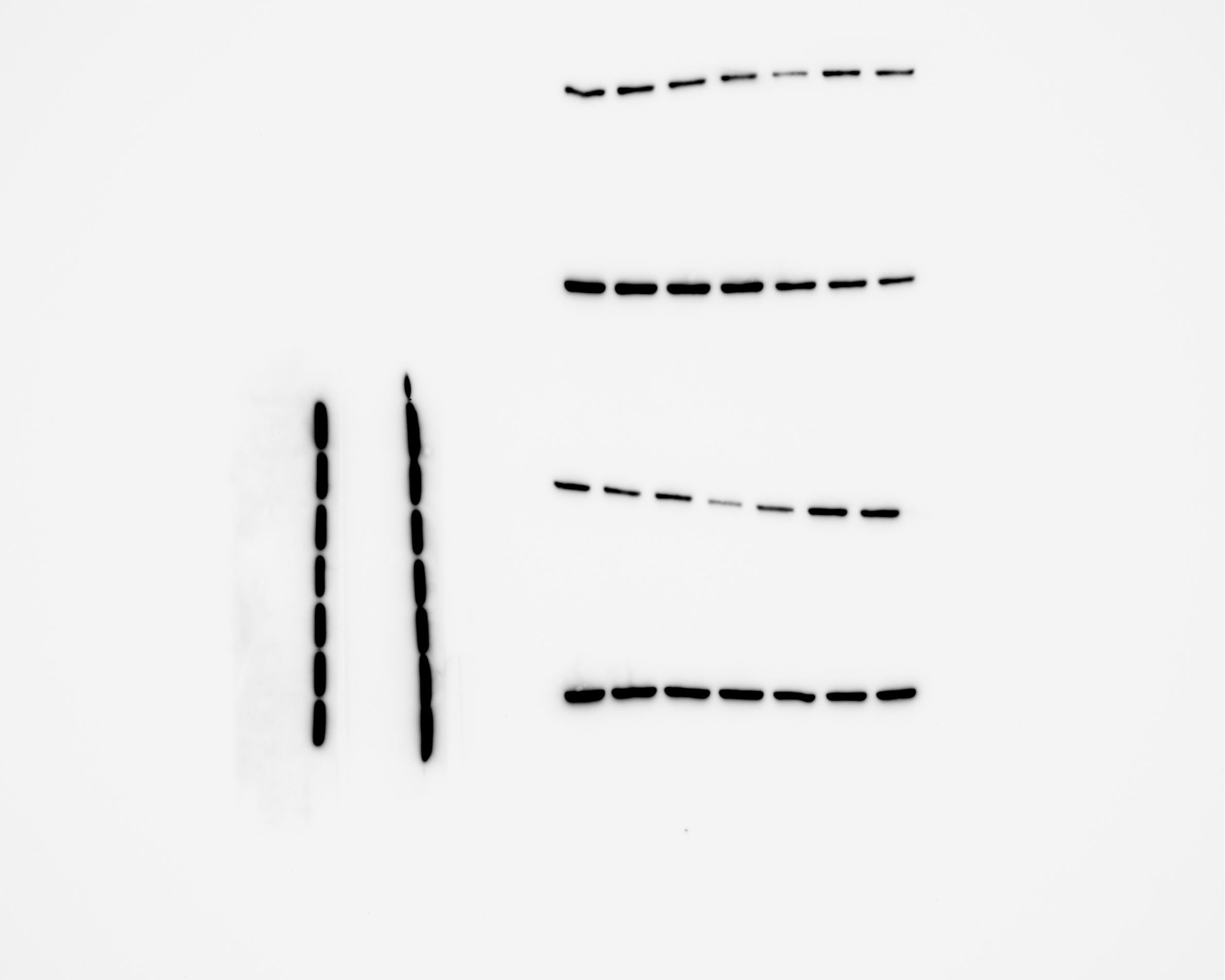

Supplement: Figure 1—source data 5. [file elife-80156-fig1-data5.tif]

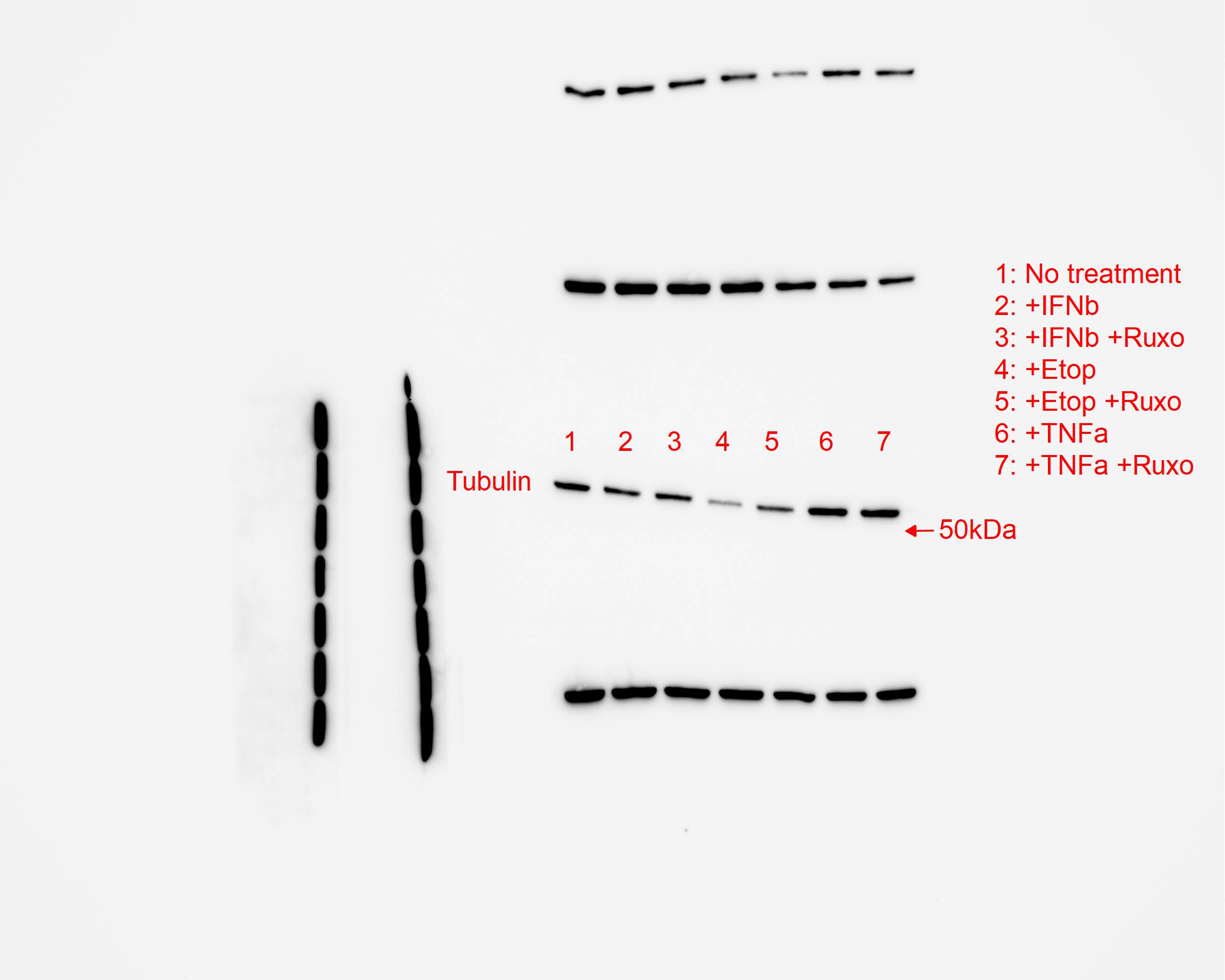

Supplement: Figure 1—source data 6. [file elife-80156-fig1-data6.tif]

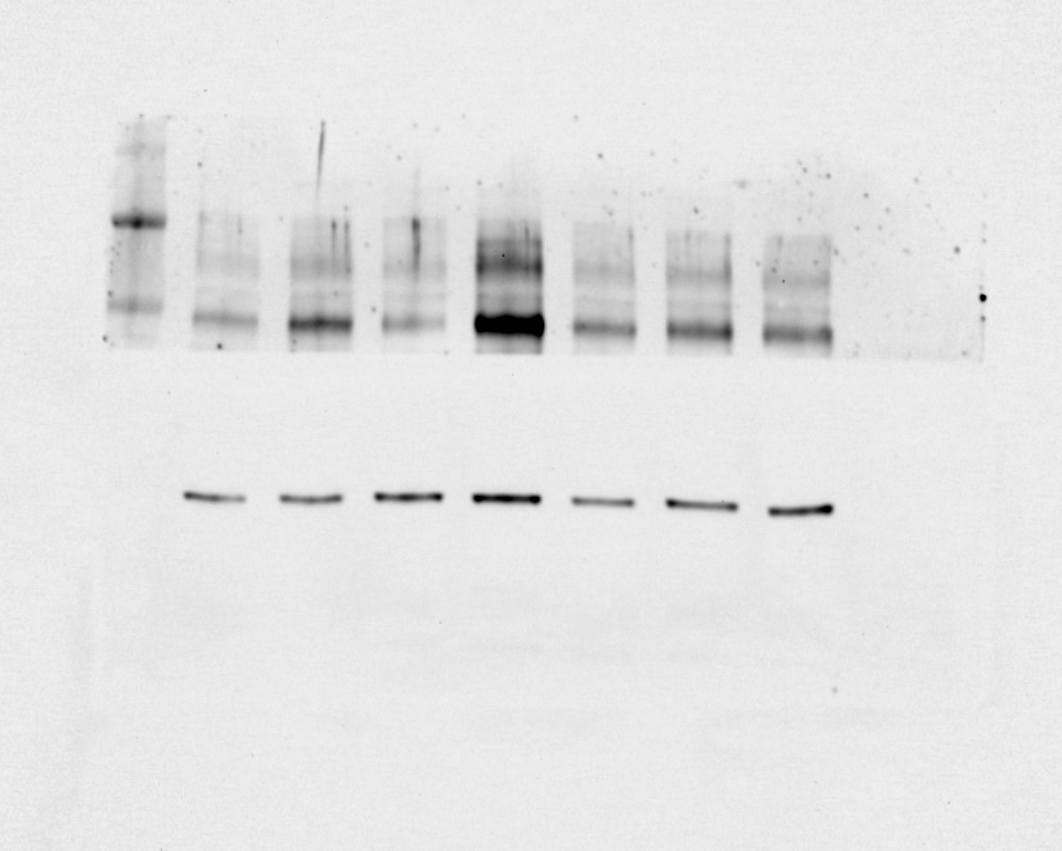

Supplement: Figure 1—source data 7. [file elife-80156-fig1-data7.tif]

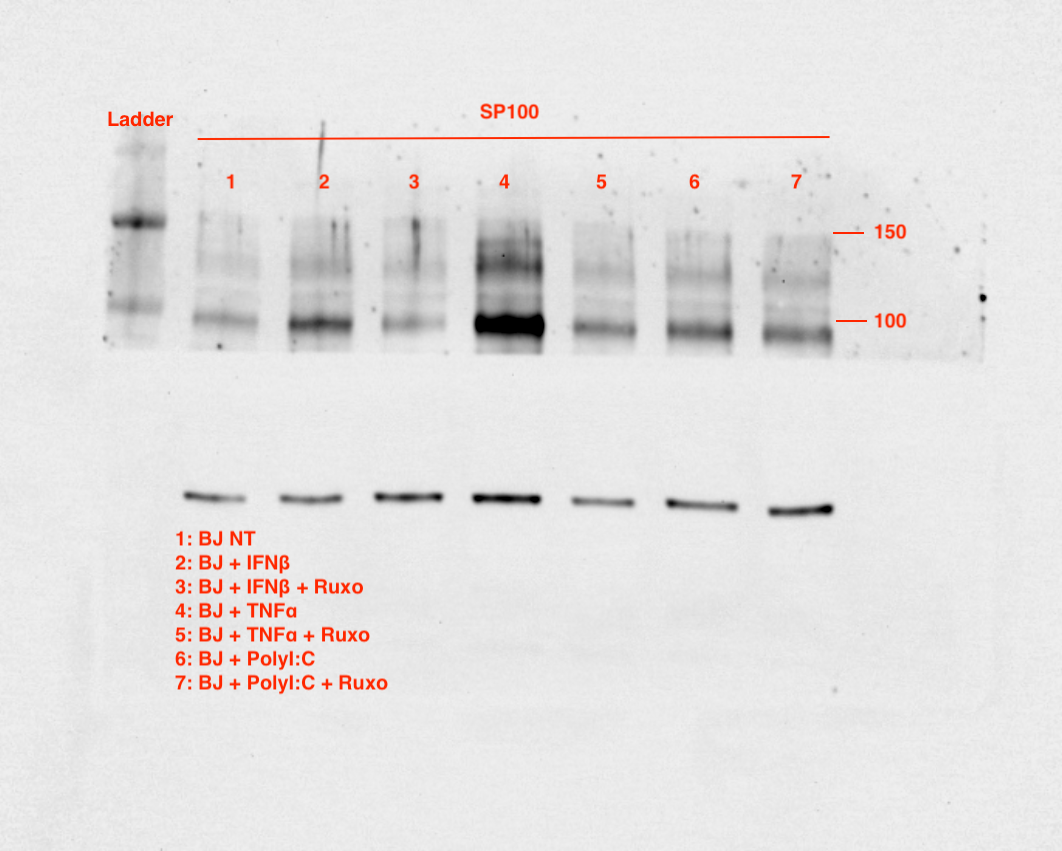

Supplement: Figure 1—source data 8. [file elife-80156-fig1-data8.tif]

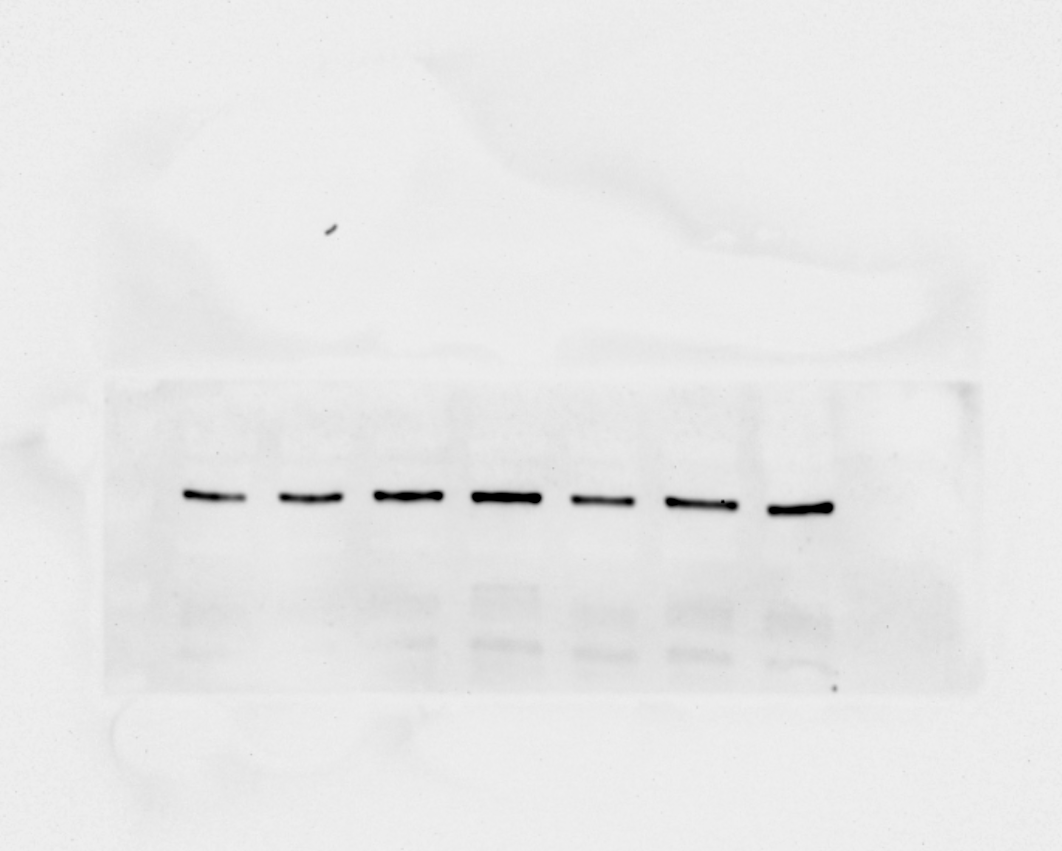

Supplement: Figure 1—source data 9. [file elife-80156-fig1-data9.tif]

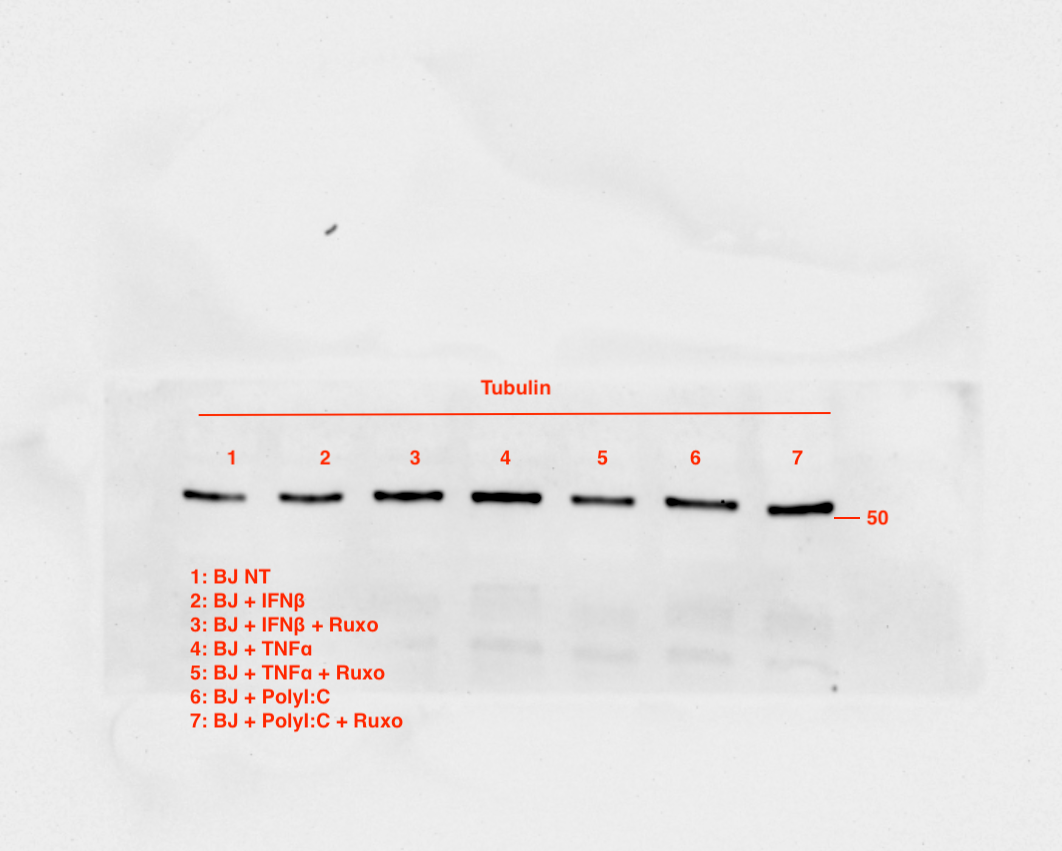

Supplement: Figure 1—source data 10. [file elife-80156-fig1-data10.tif]

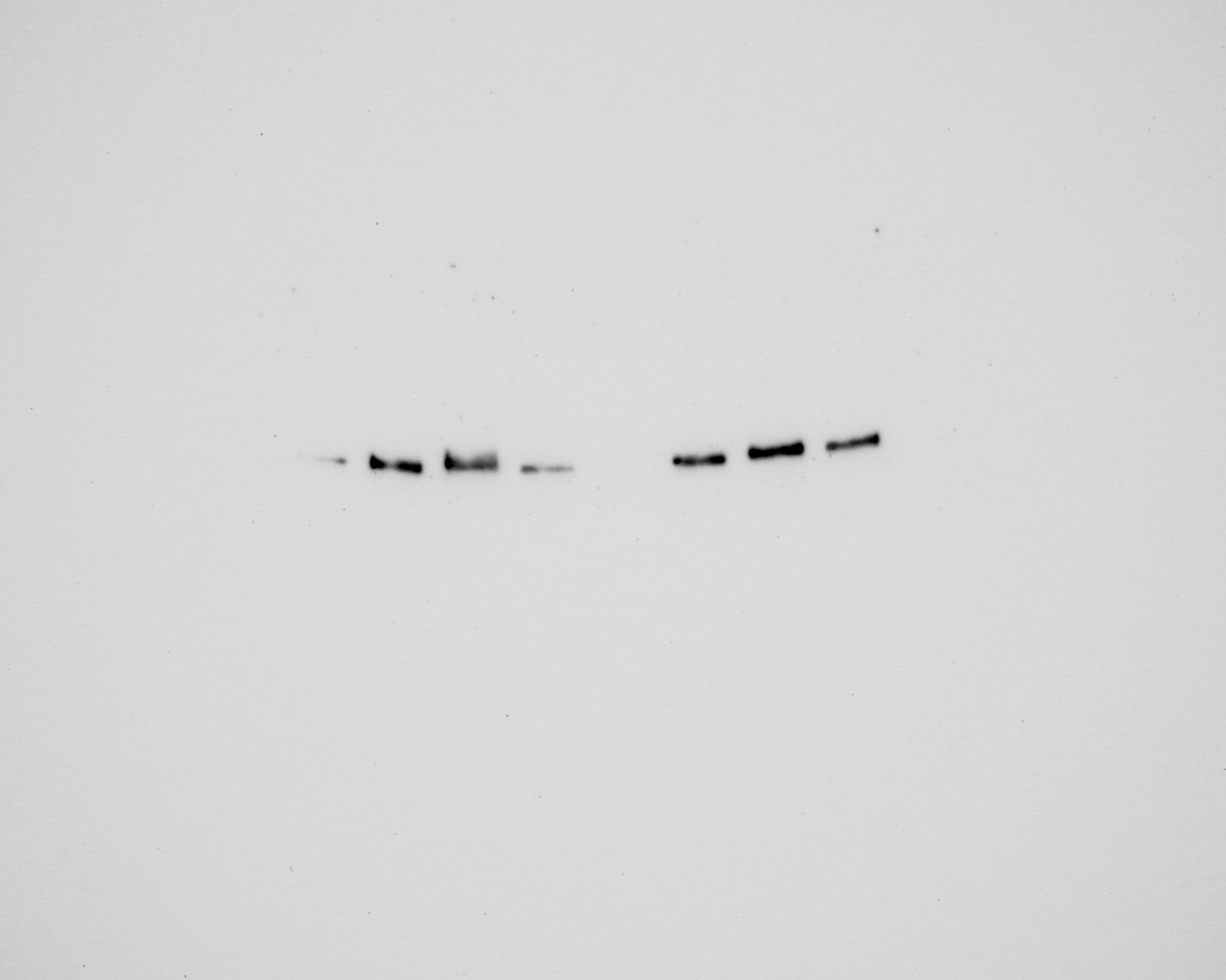

Supplement: Figure 1—figure supplement 1—source data 1. [file elife-80156-fig1-figsupp1-data1.tif]

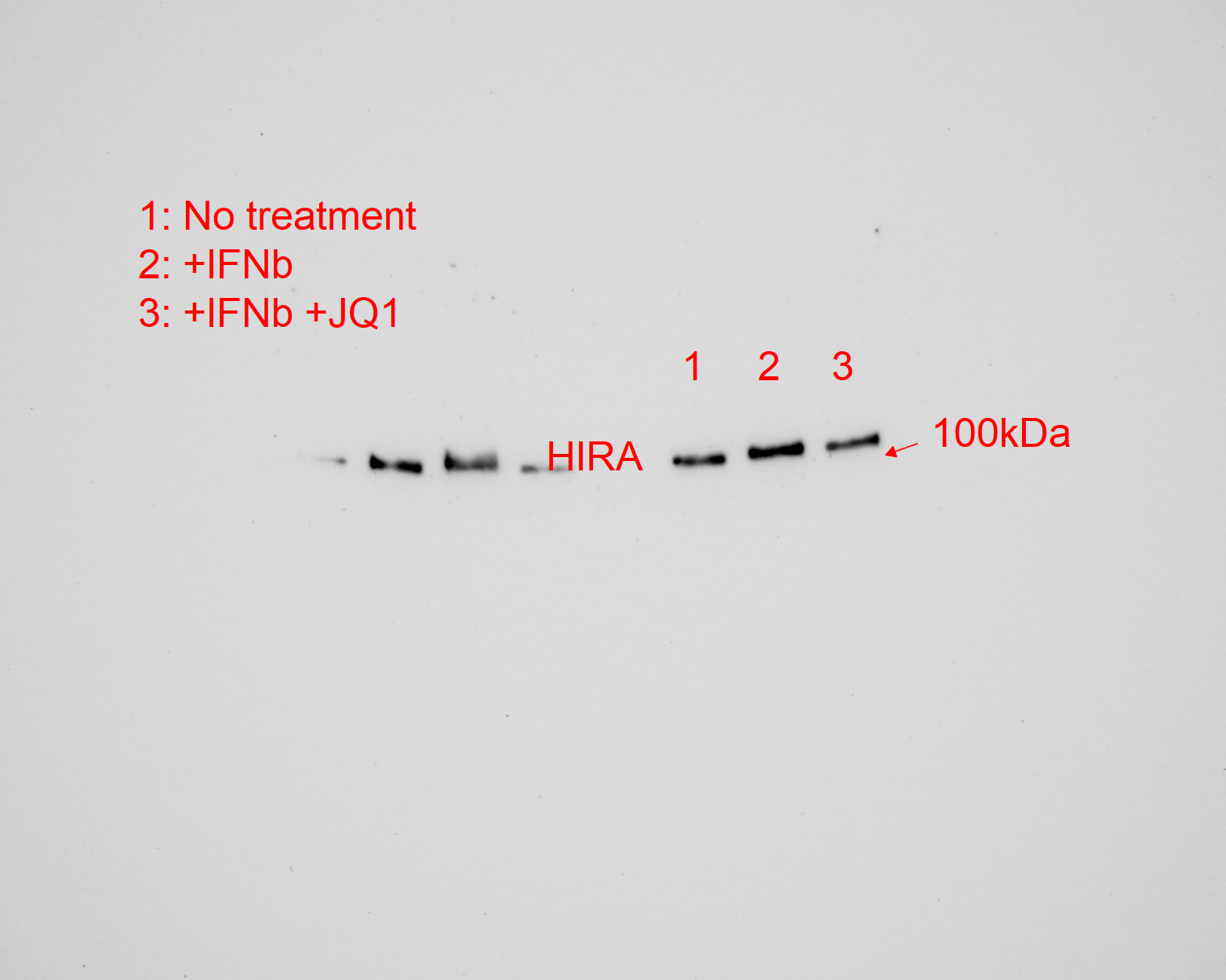

Supplement: Figure 1—figure supplement 1—source data 2. [file elife-80156-fig1-figsupp1-data2.tif]

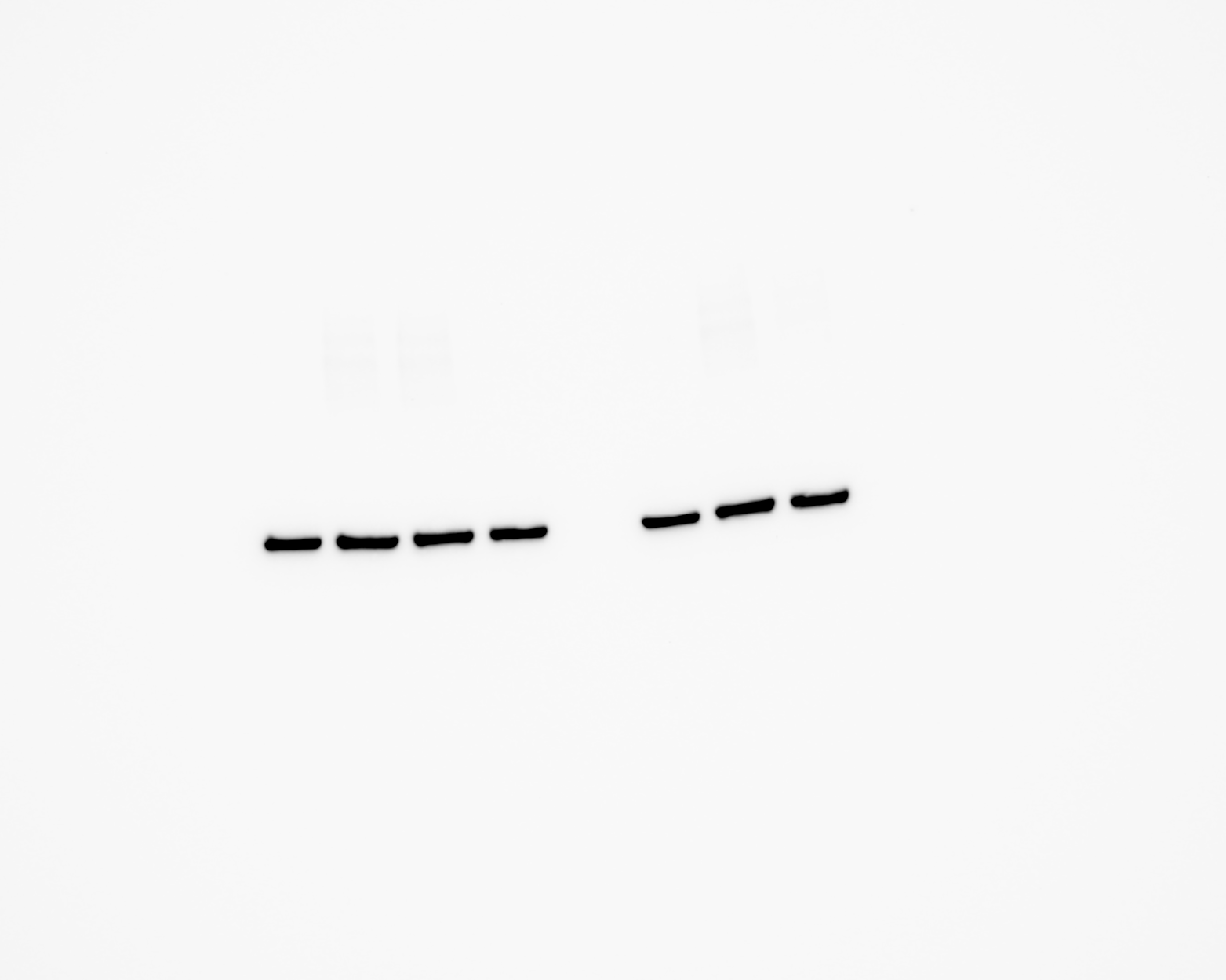

Supplement: Figure 1—figure supplement 1—source data 3. [file elife-80156-fig1-figsupp1-data3.tif]

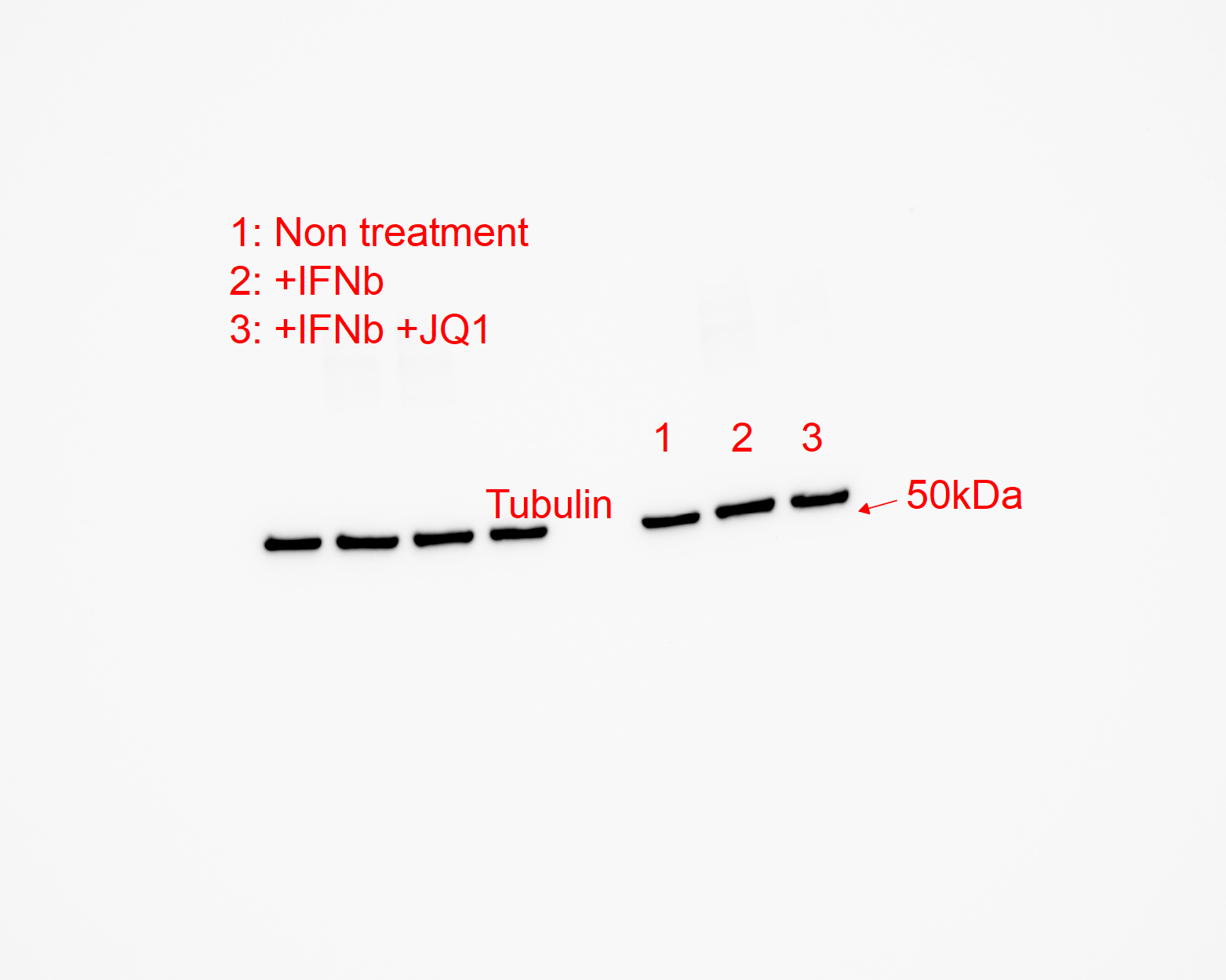

Supplement: Figure 1—figure supplement 1—source data 4. [file elife-80156-fig1-figsupp1-data4.tif]

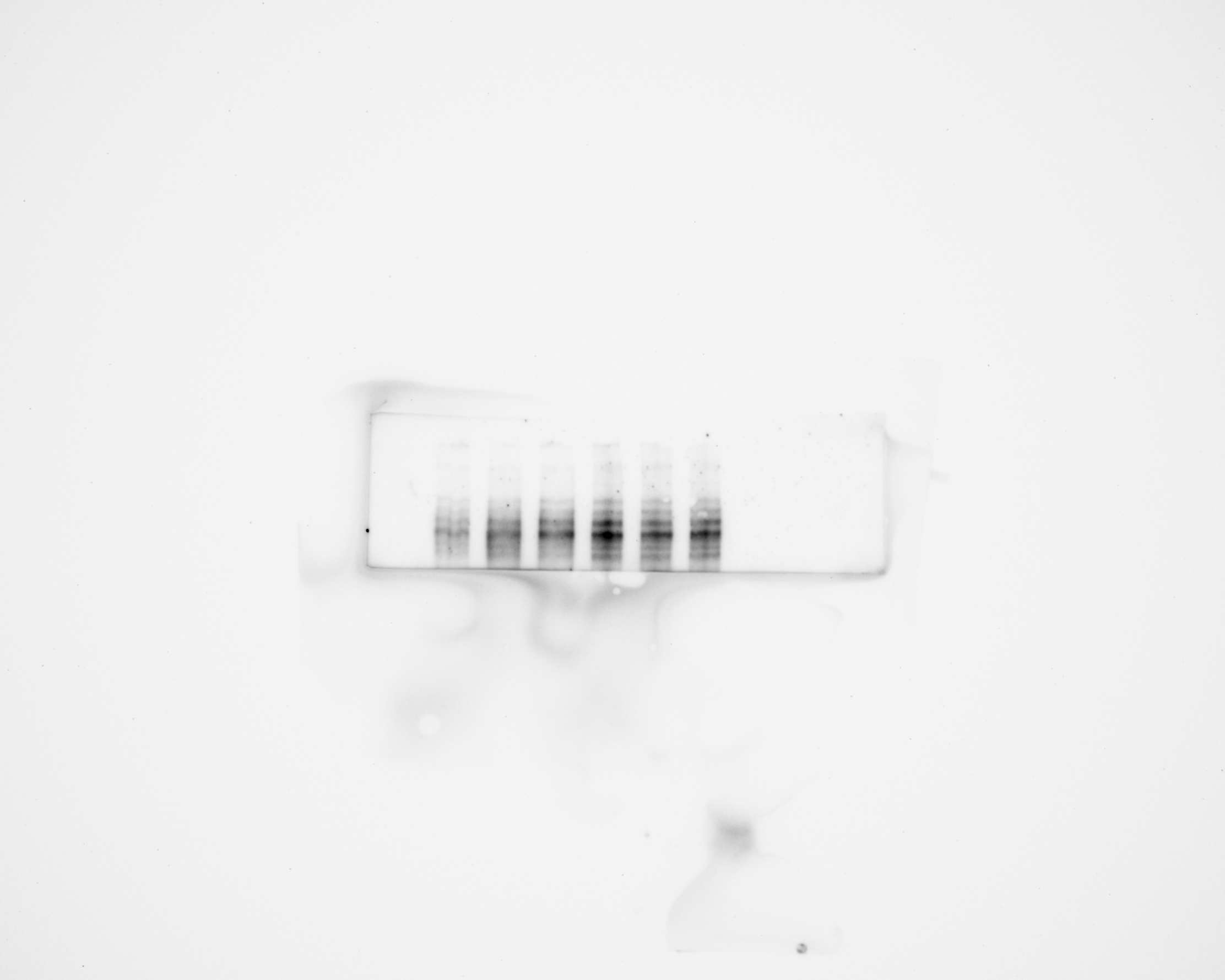

Supplement: Figure 1—figure supplement 1—source data 5. [file elife-80156-fig1-figsupp1-data5.tif]

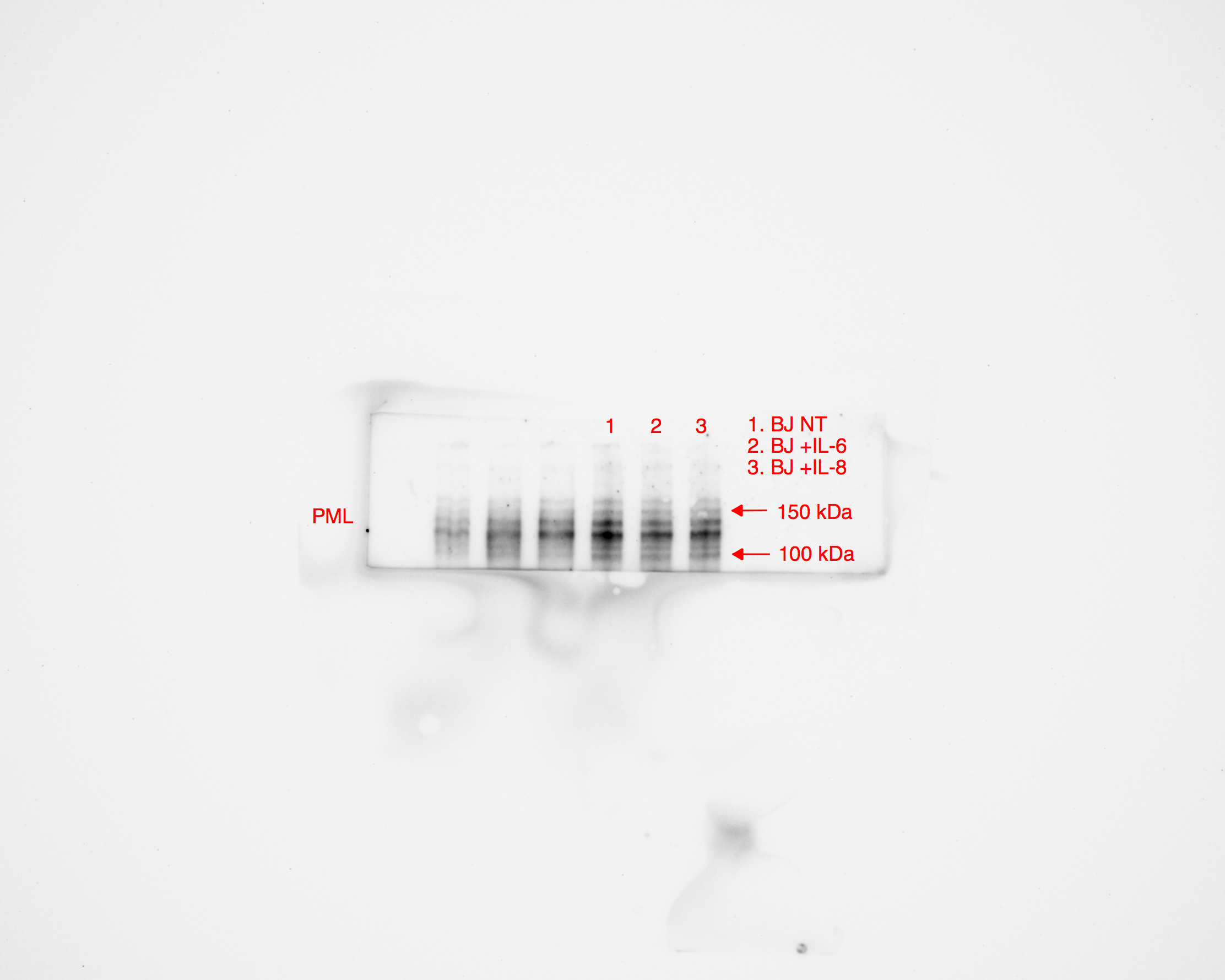

Supplement: Figure 1—figure supplement 1—source data 6. [file elife-80156-fig1-figsupp1-data6.tif]

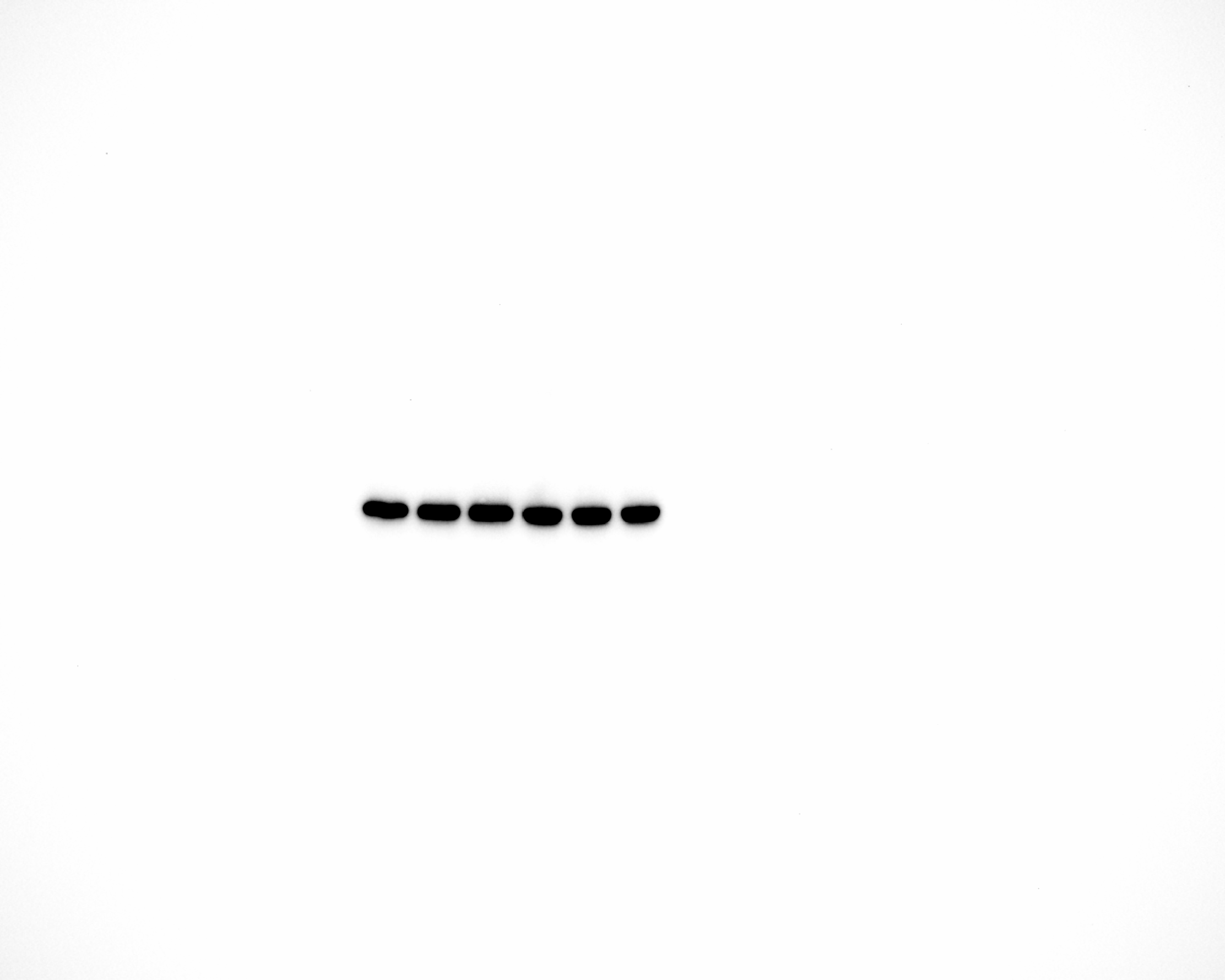

Supplement: Figure 1—figure supplement 1—source data 7. [file elife-80156-fig1-figsupp1-data7.tif]

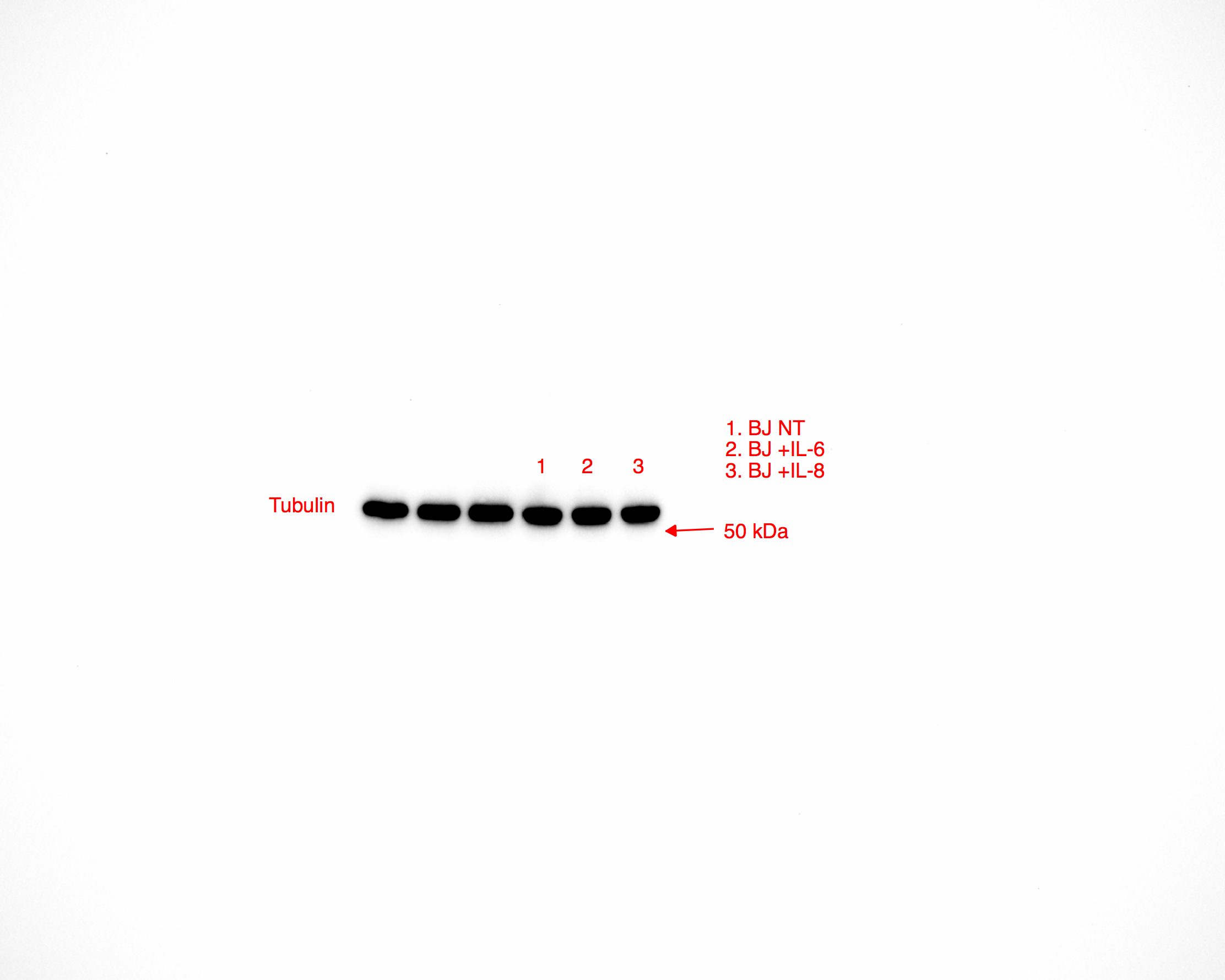

Supplement: Figure 1—figure supplement 1—source data 8. [file elife-80156-fig1-figsupp1-data8.tif]

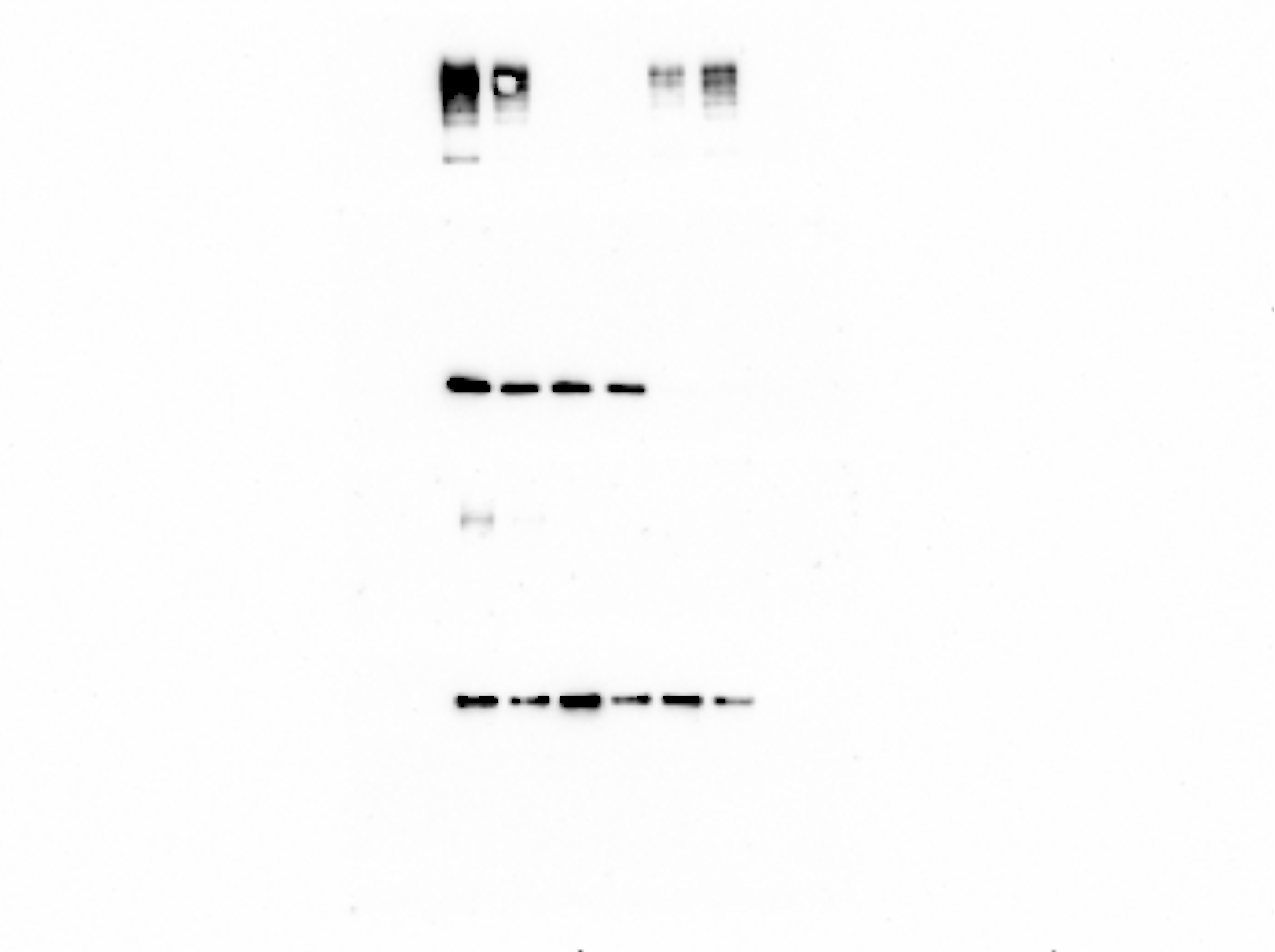

Supplement: Figure 2—source data 1. [file elife-80156-fig2-data1.tif]

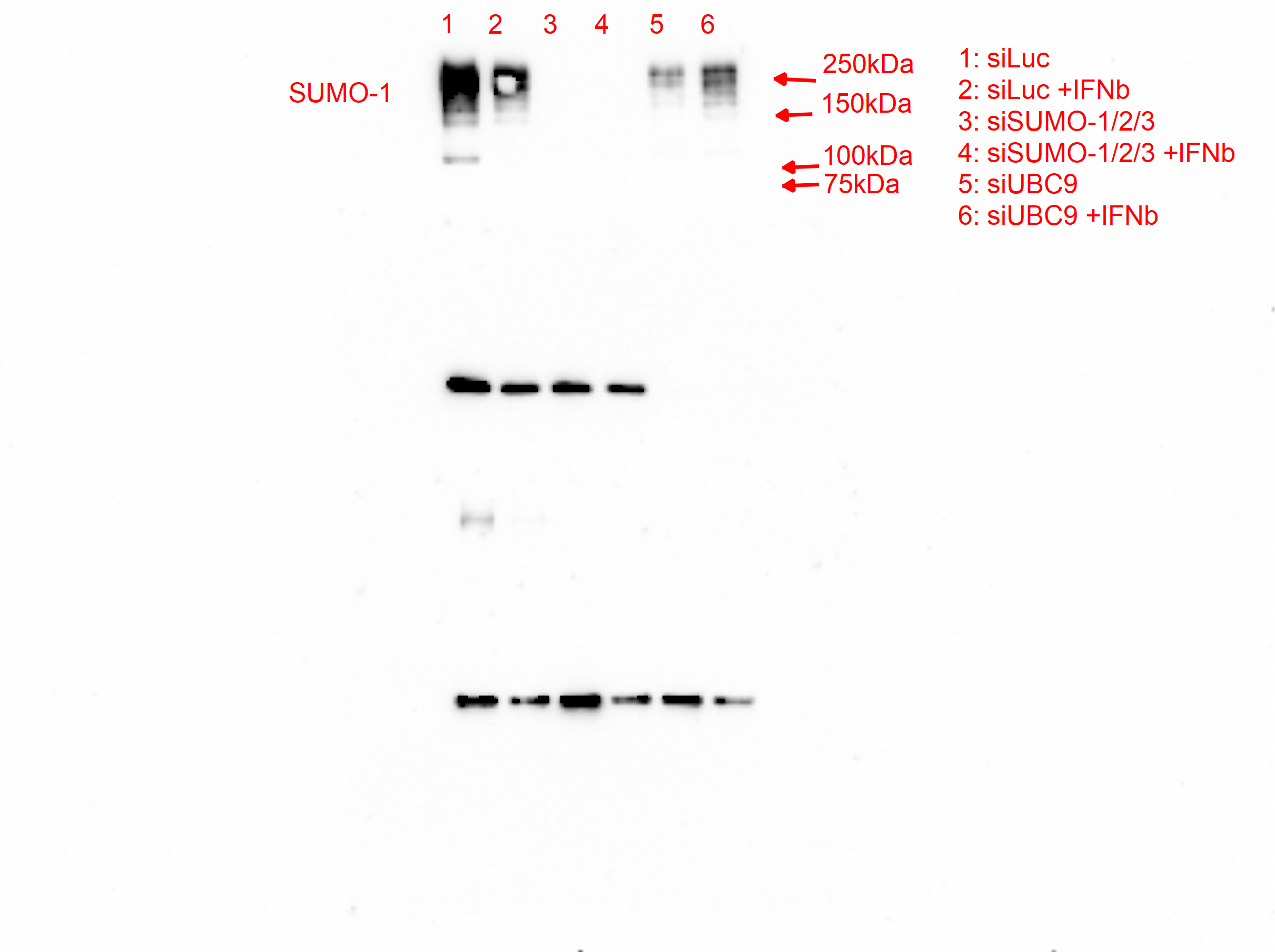

Supplement: Figure 2—source data 2. [file elife-80156-fig2-data2.tif]

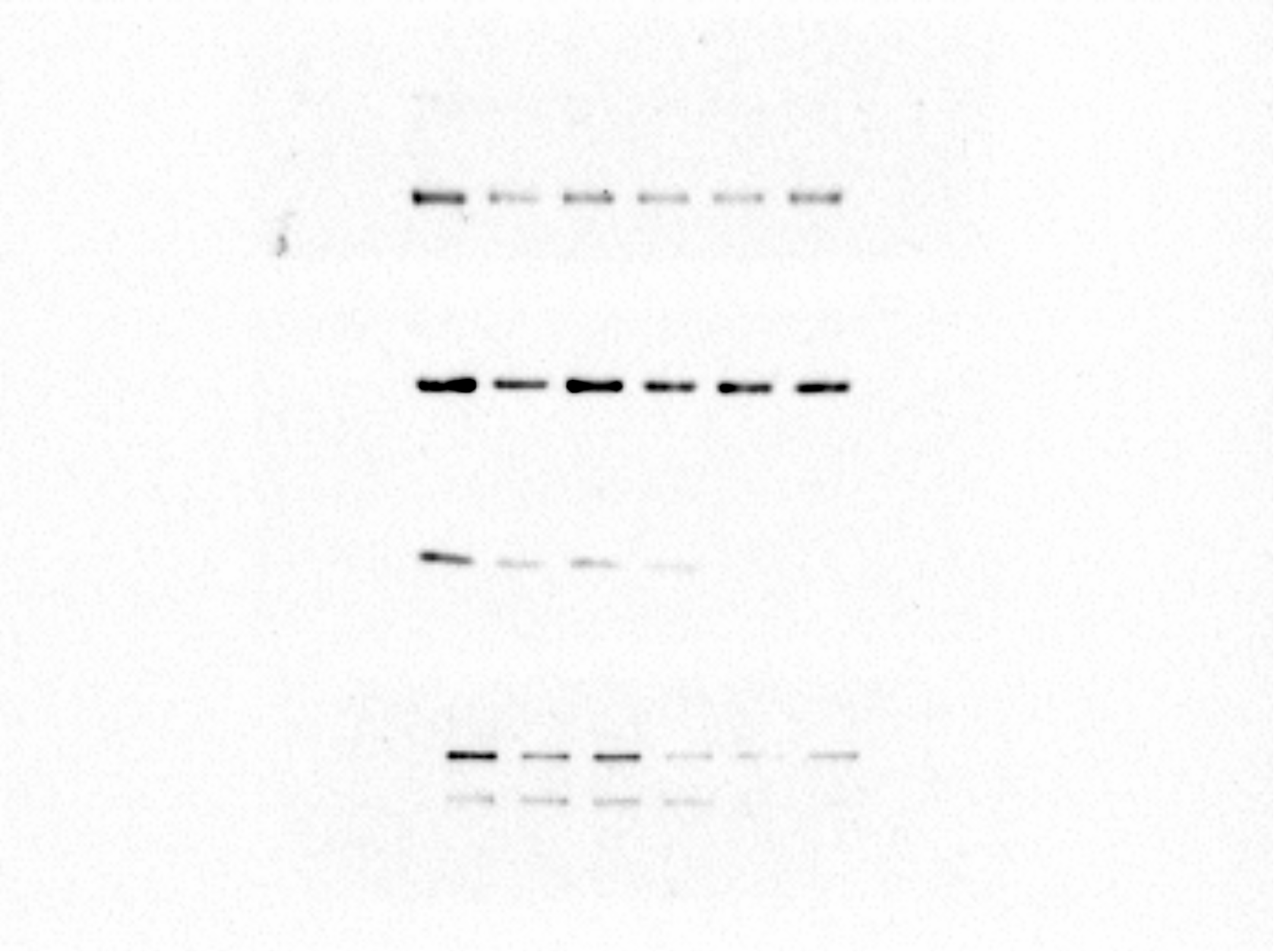

Supplement: Figure 2—source data 3. [file elife-80156-fig2-data3.tif]

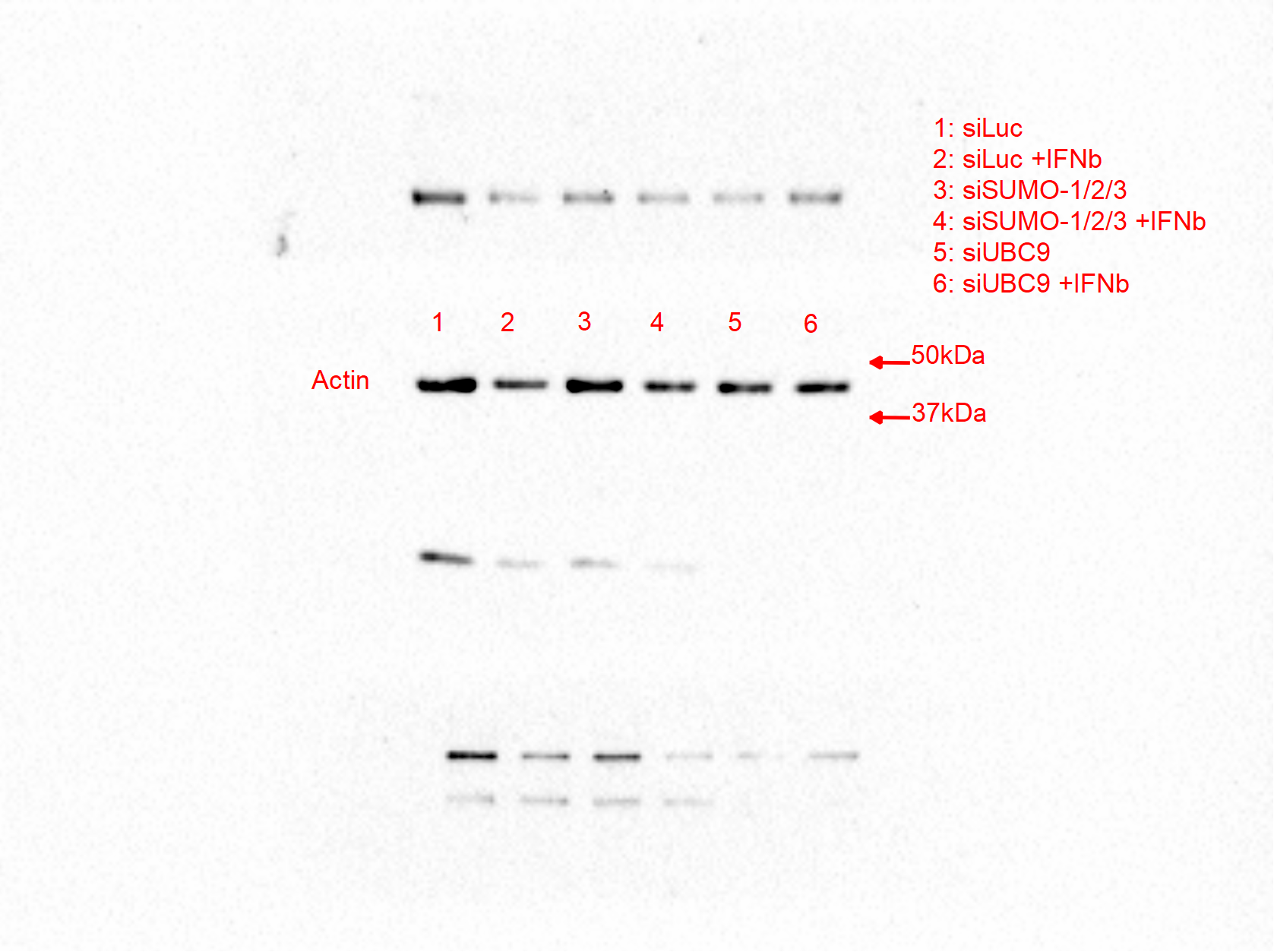

Supplement: Figure 2—source data 4. [file elife-80156-fig2-data4.tif]

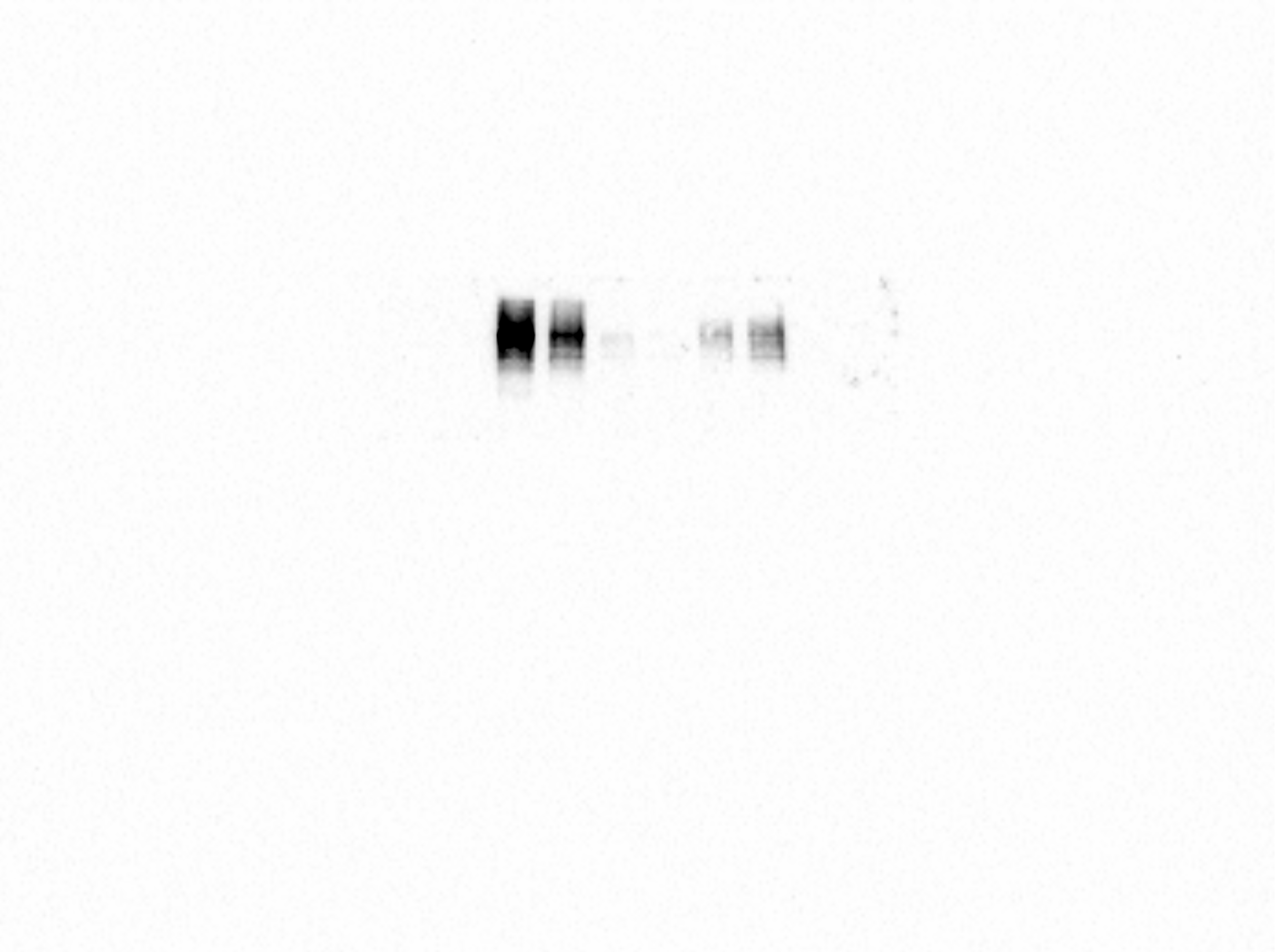

Supplement: Figure 2—source data 5. [file elife-80156-fig2-data5.tif]

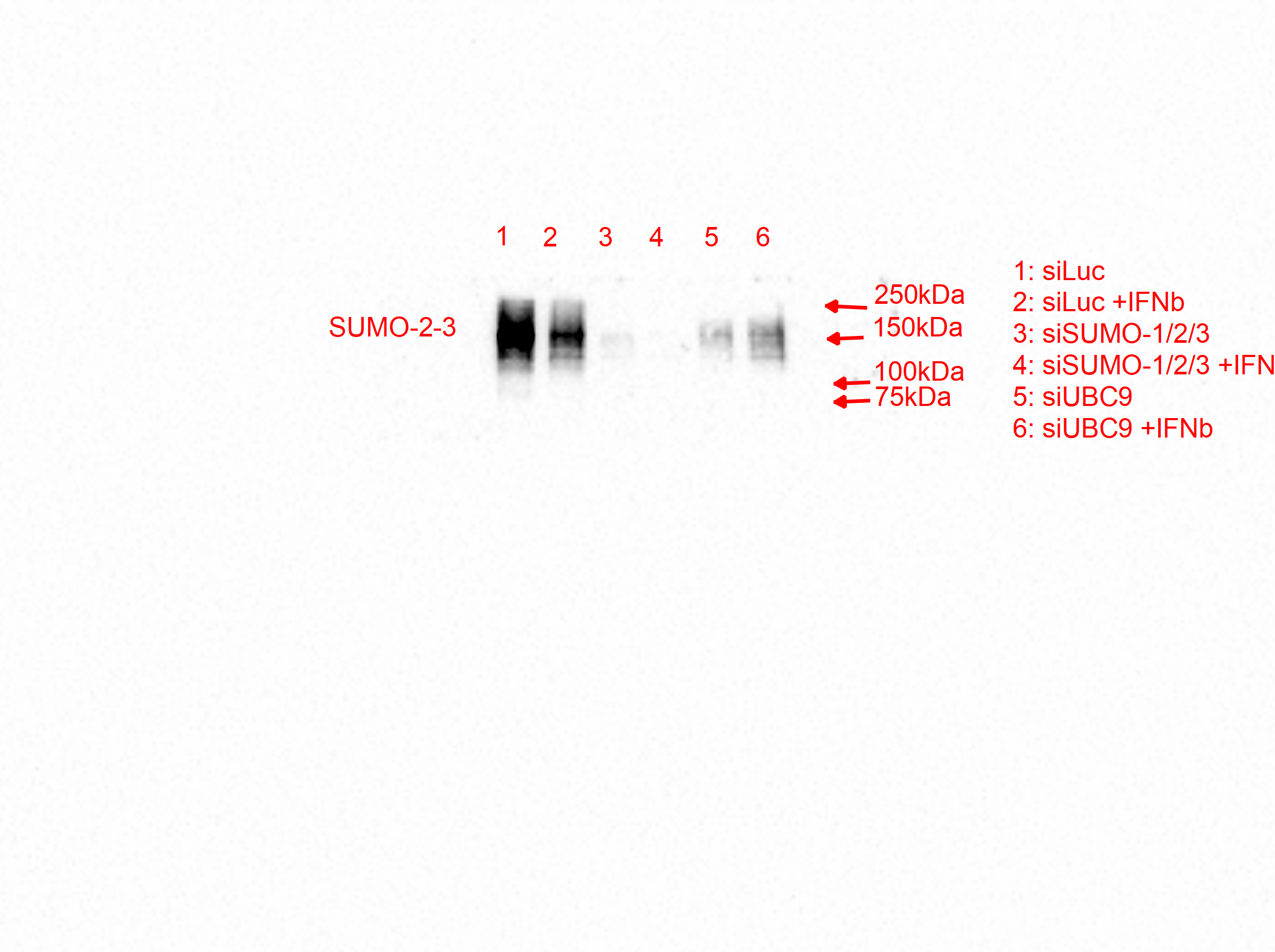

Supplement: Figure 2—source data 6. [file elife-80156-fig2-data6.tif]

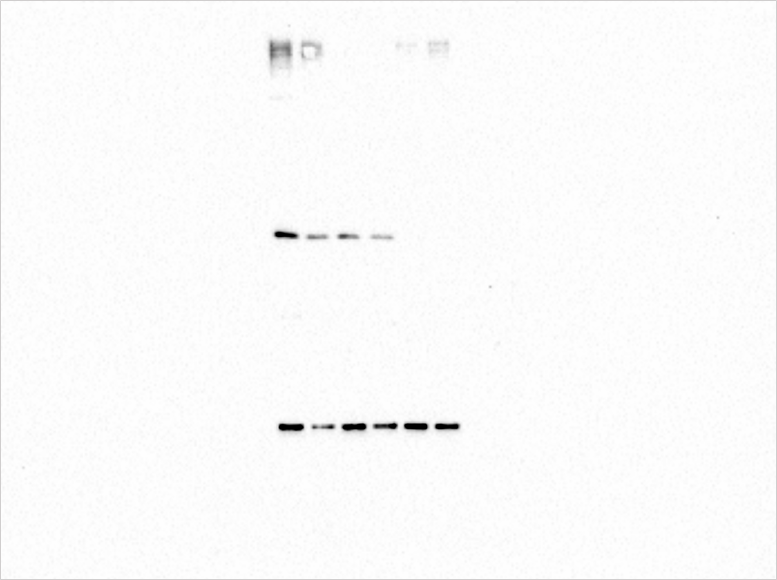

Supplement: Figure 2—source data 7. [file elife-80156-fig2-data7.tif]

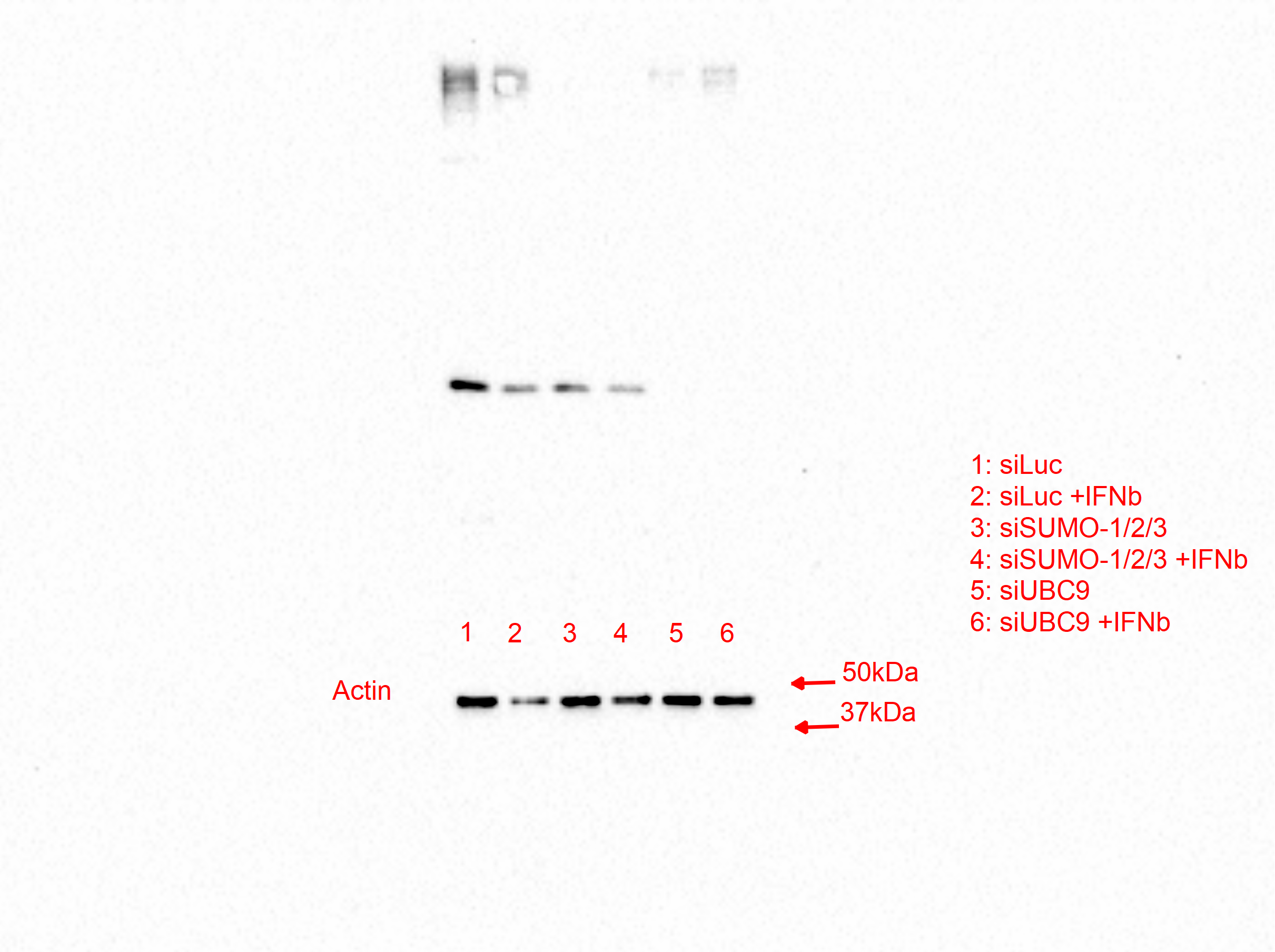

Supplement: Figure 2—source data 8. [file elife-80156-fig2-data8.tif]

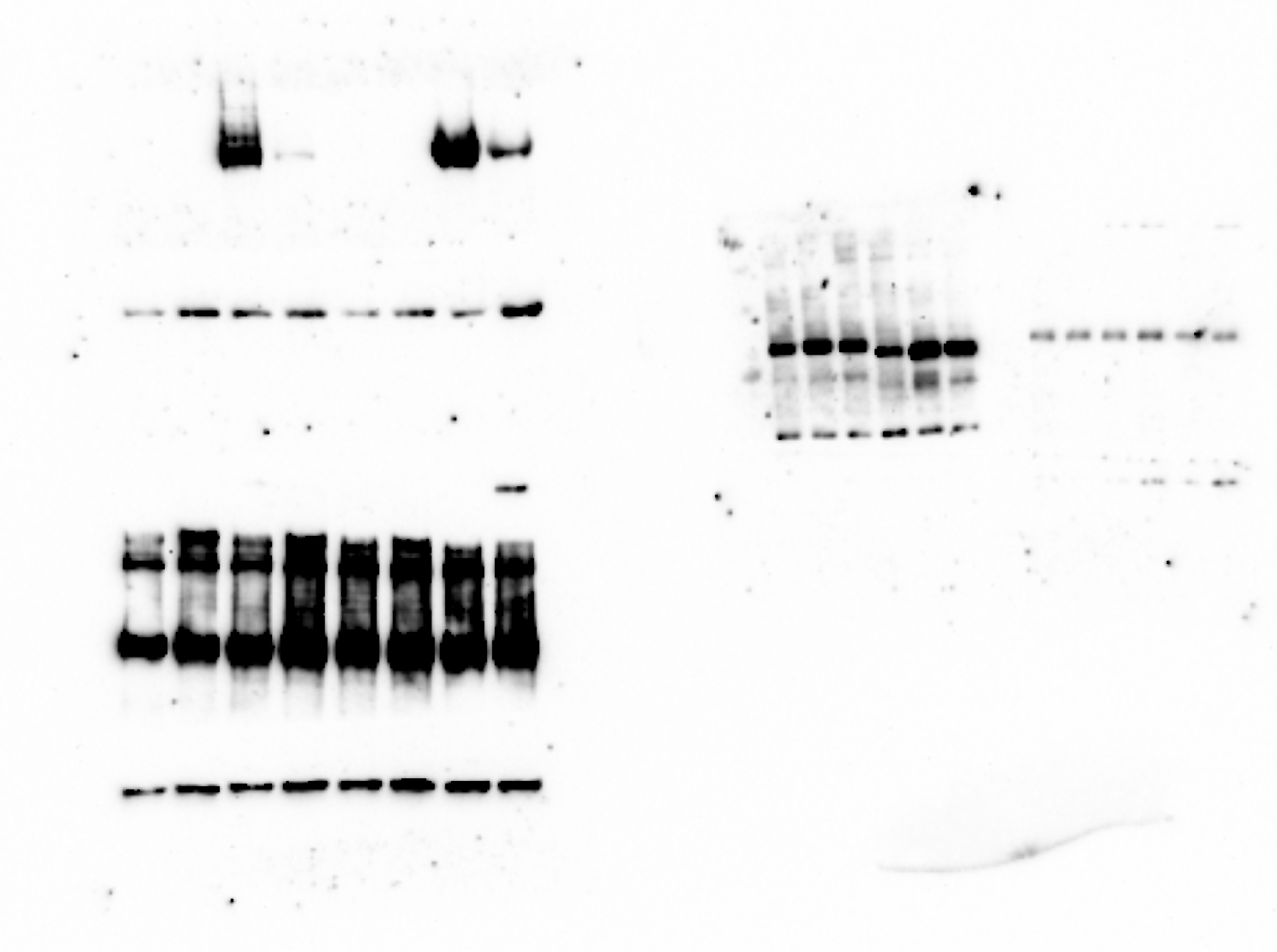

Supplement: Figure 3—source data 1. [file elife-80156-fig3-data1.tif]

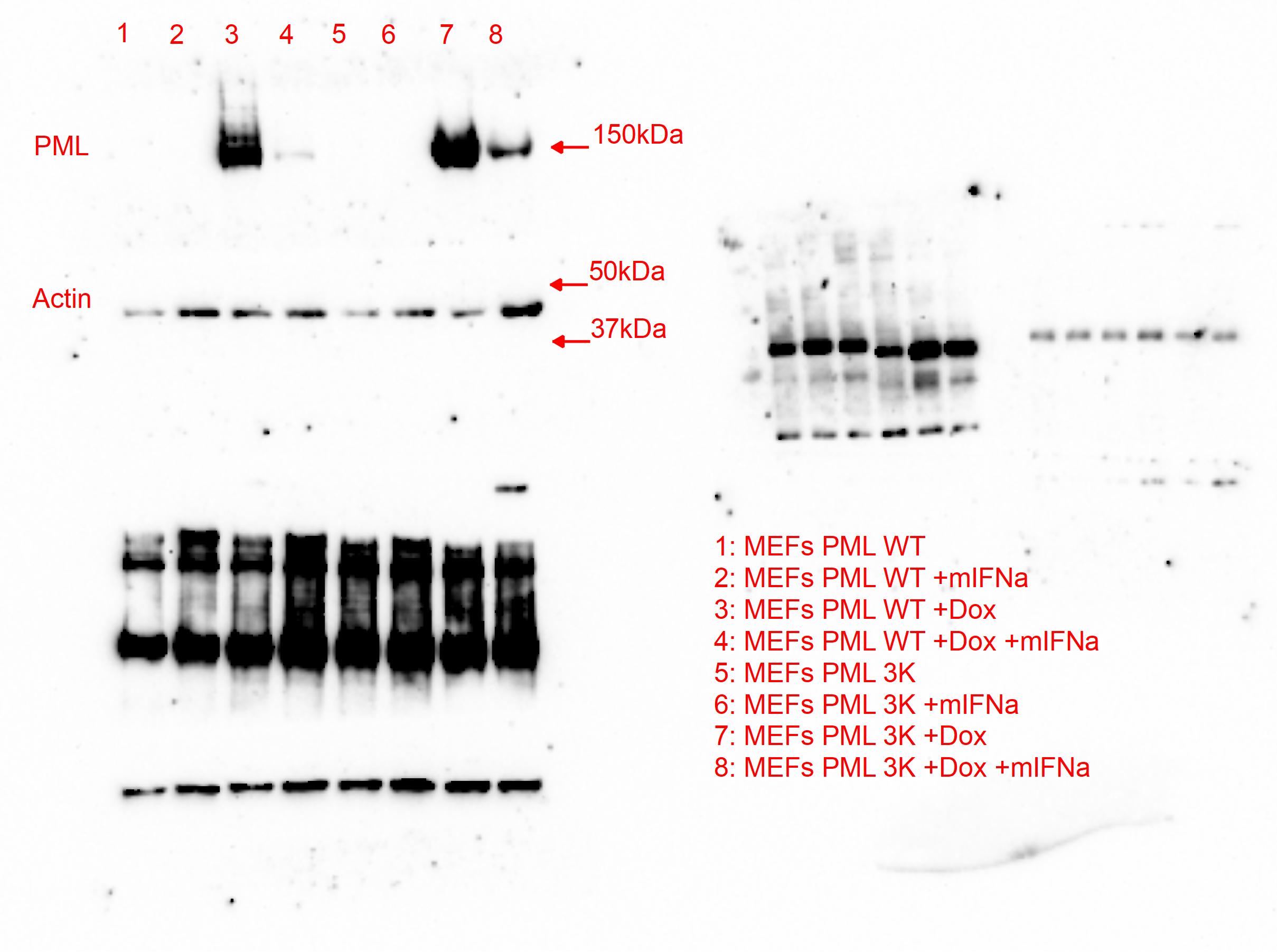

Supplement: Figure 3—source data 2. [file elife-80156-fig3-data2.tif]

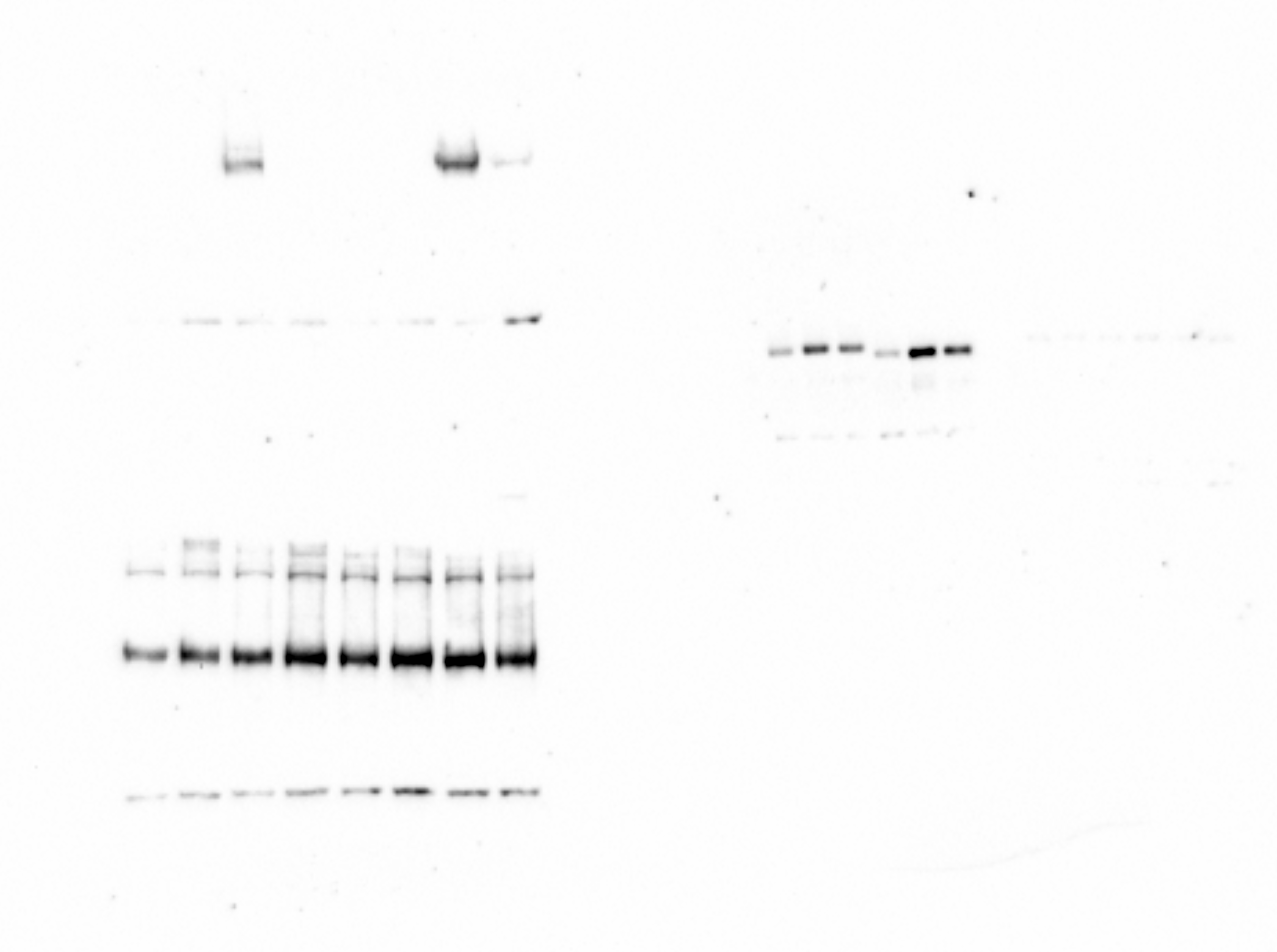

Supplement: Figure 3—source data 3. [file elife-80156-fig3-data3.tif]

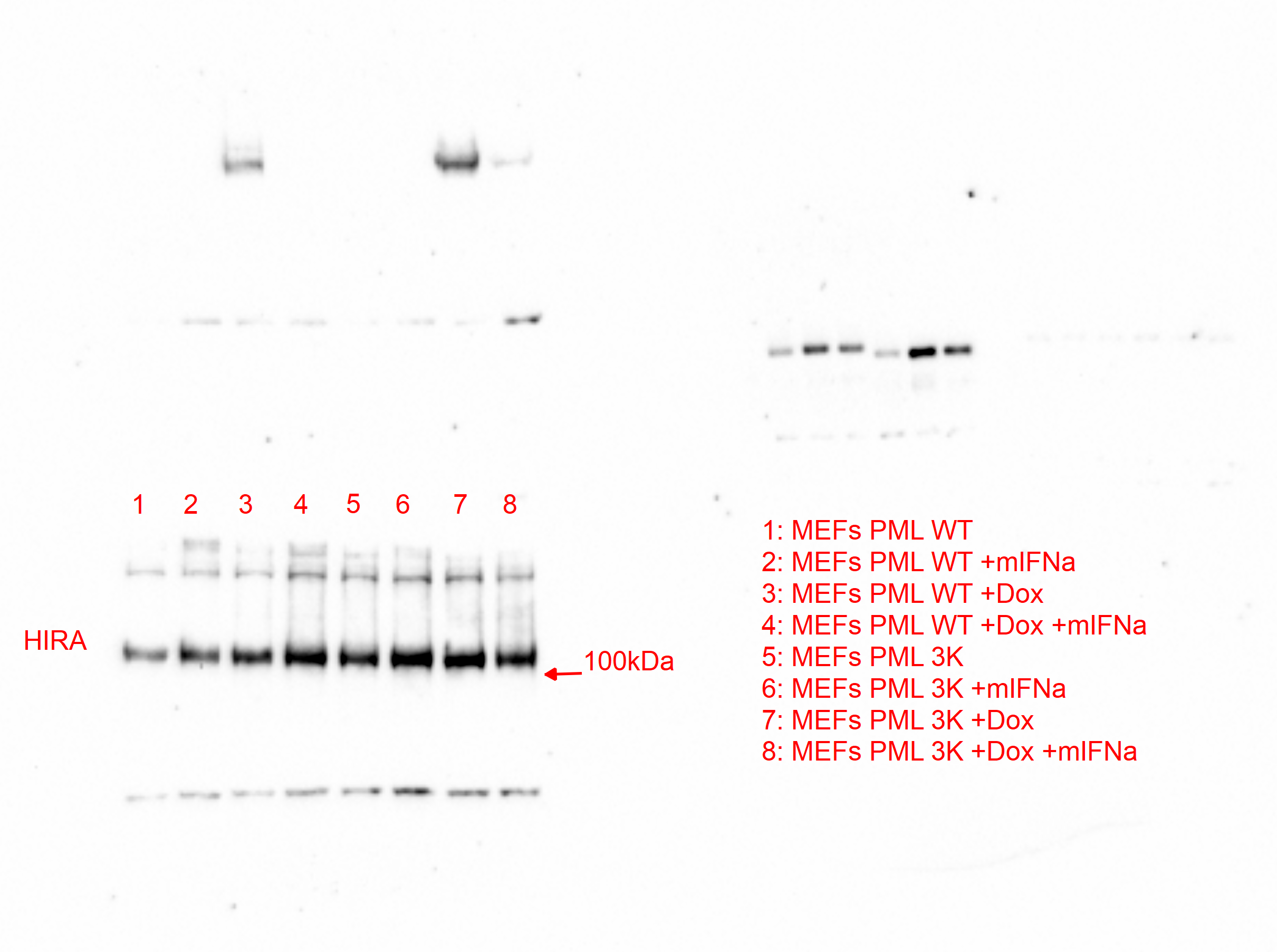

Supplement: Figure 3—source data 4. [file elife-80156-fig3-data4.tif]

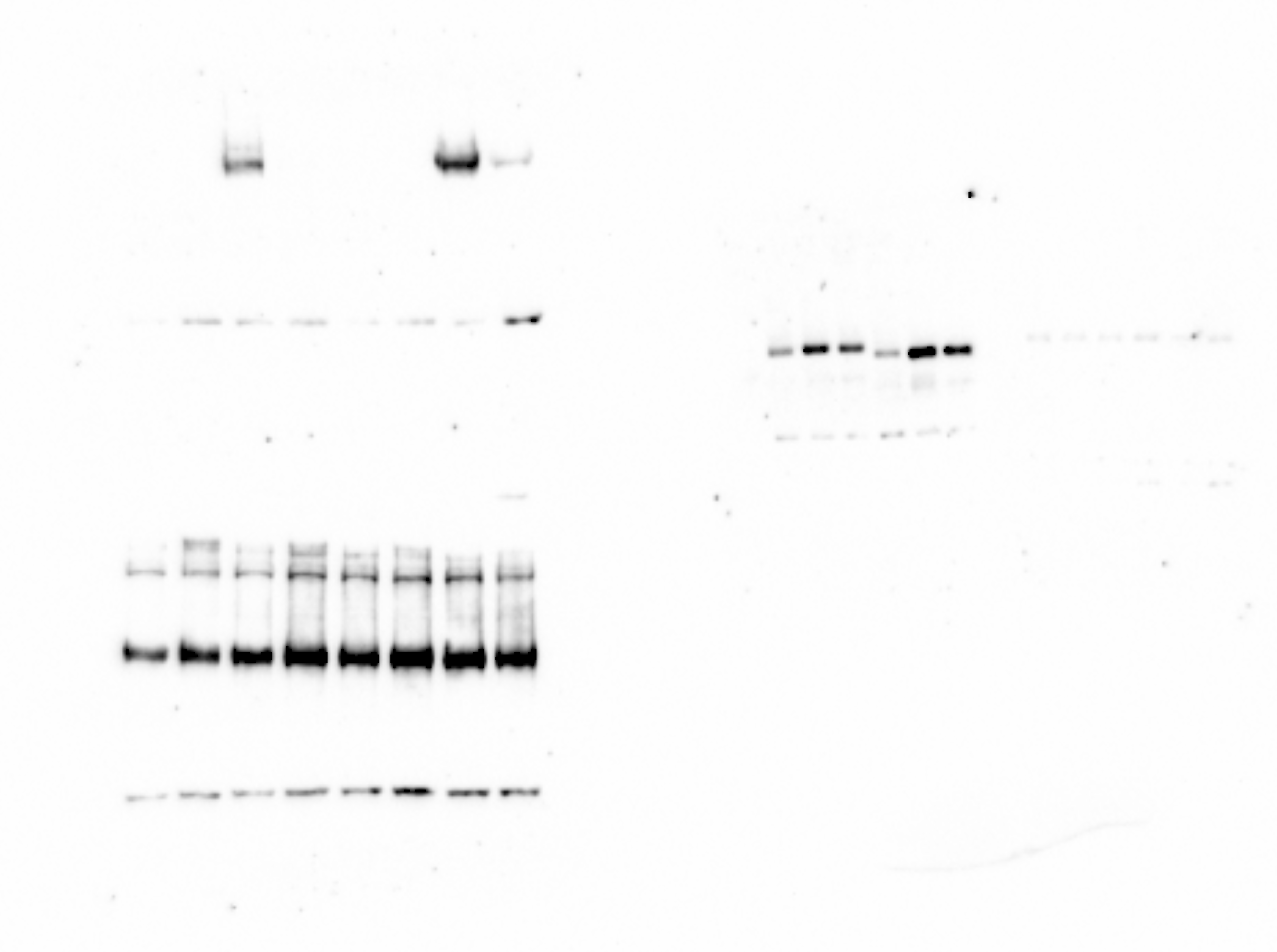

Supplement: Figure 3—source data 5. [file elife-80156-fig3-data5.tif]

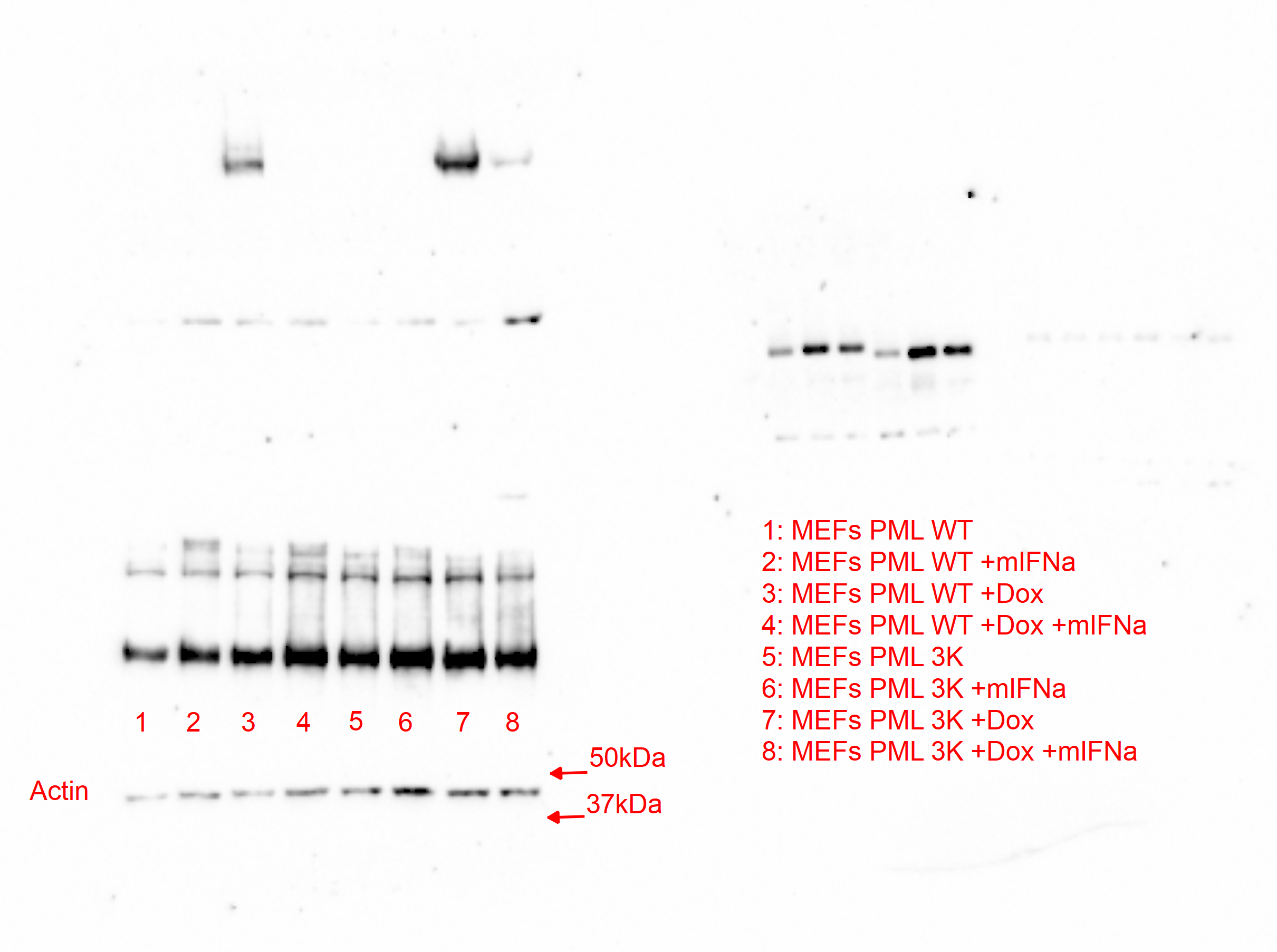

Supplement: Figure 3—source data 6. [file elife-80156-fig3-data6.tif]

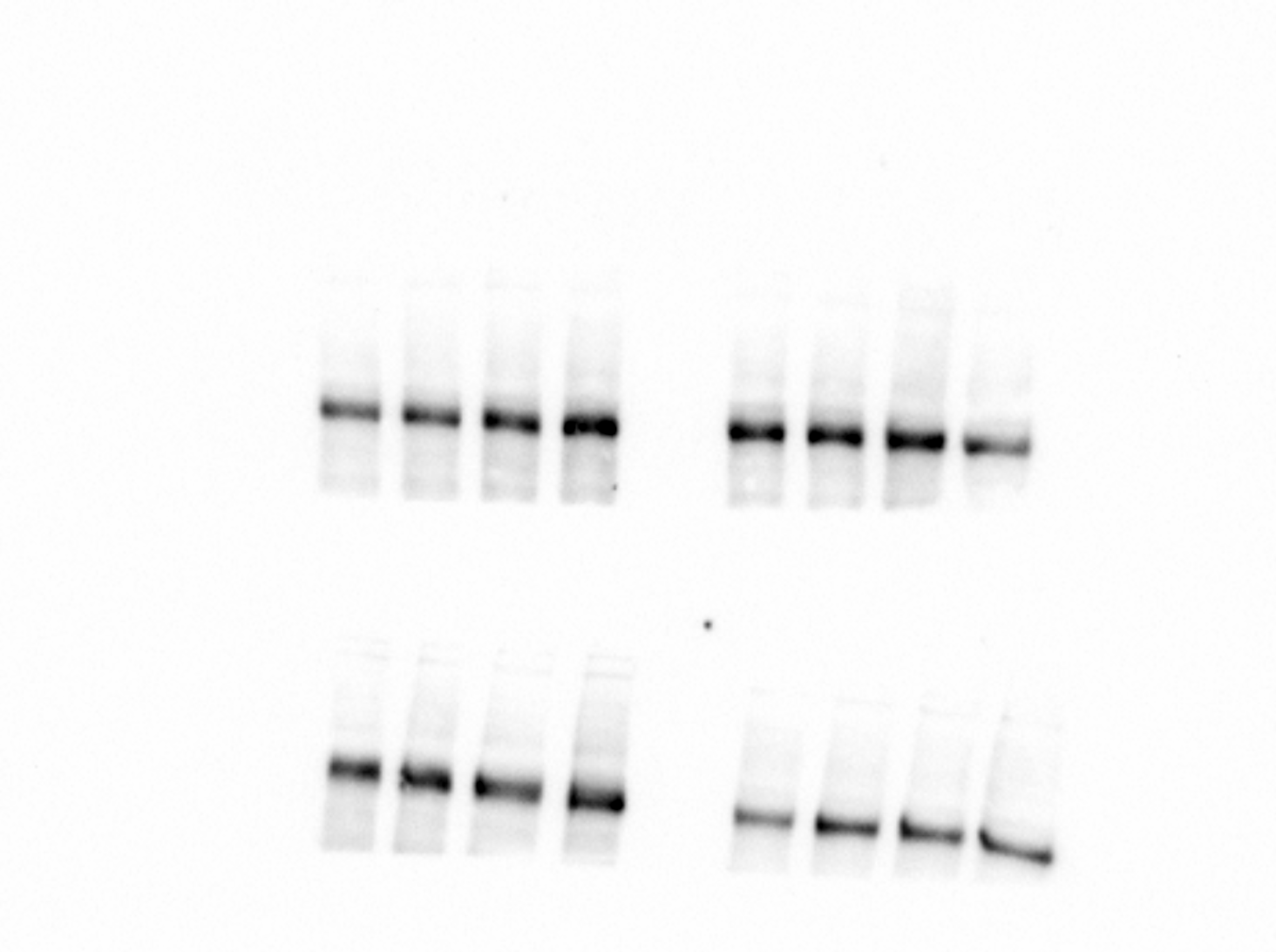

Supplement: Figure 3—figure supplement 1—source data 1. [file elife-80156-fig3-figsupp1-data1.tif]

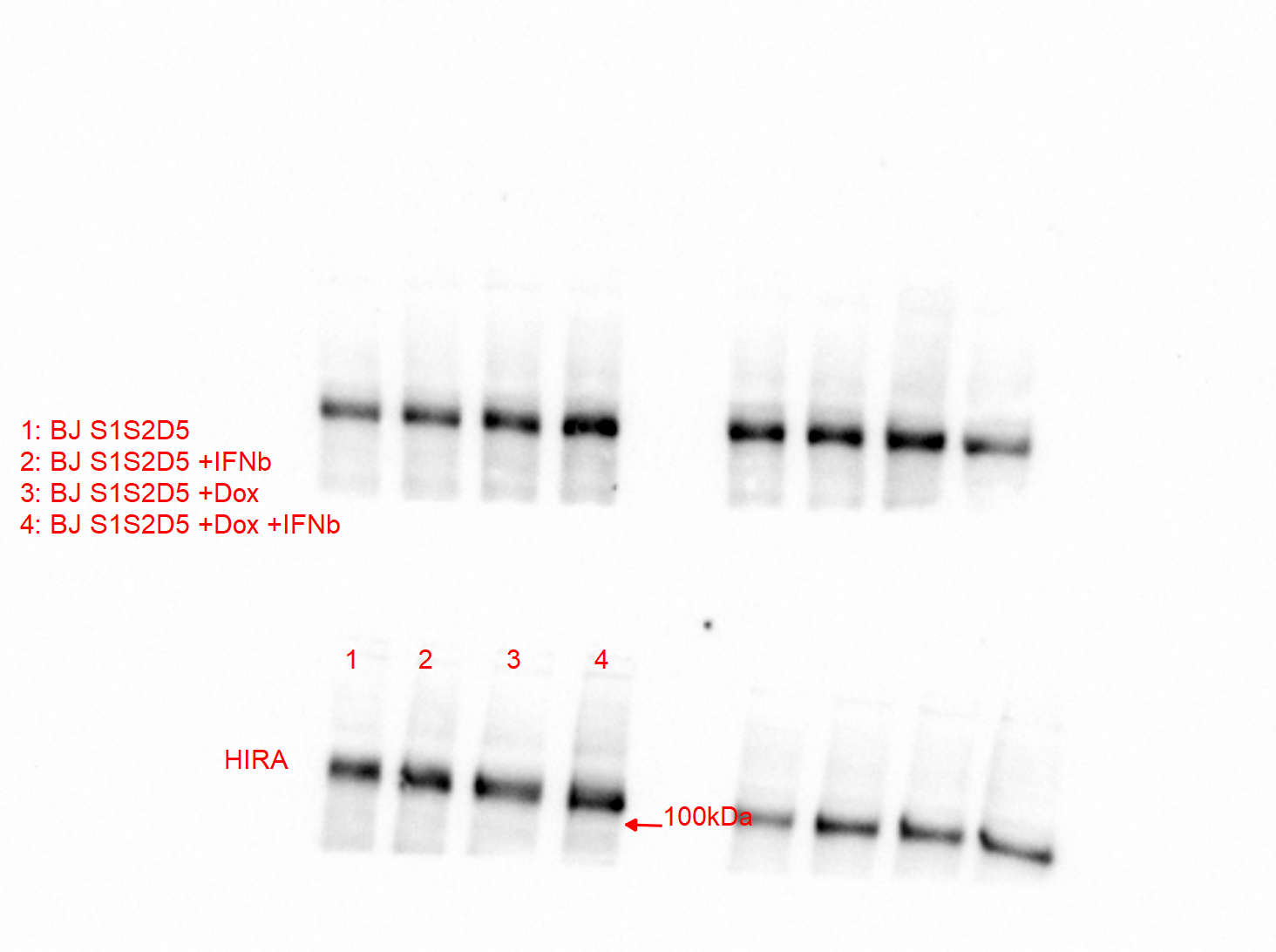

Supplement: Figure 3—figure supplement 1—source data 2. [file elife-80156-fig3-figsupp1-data2.tif]

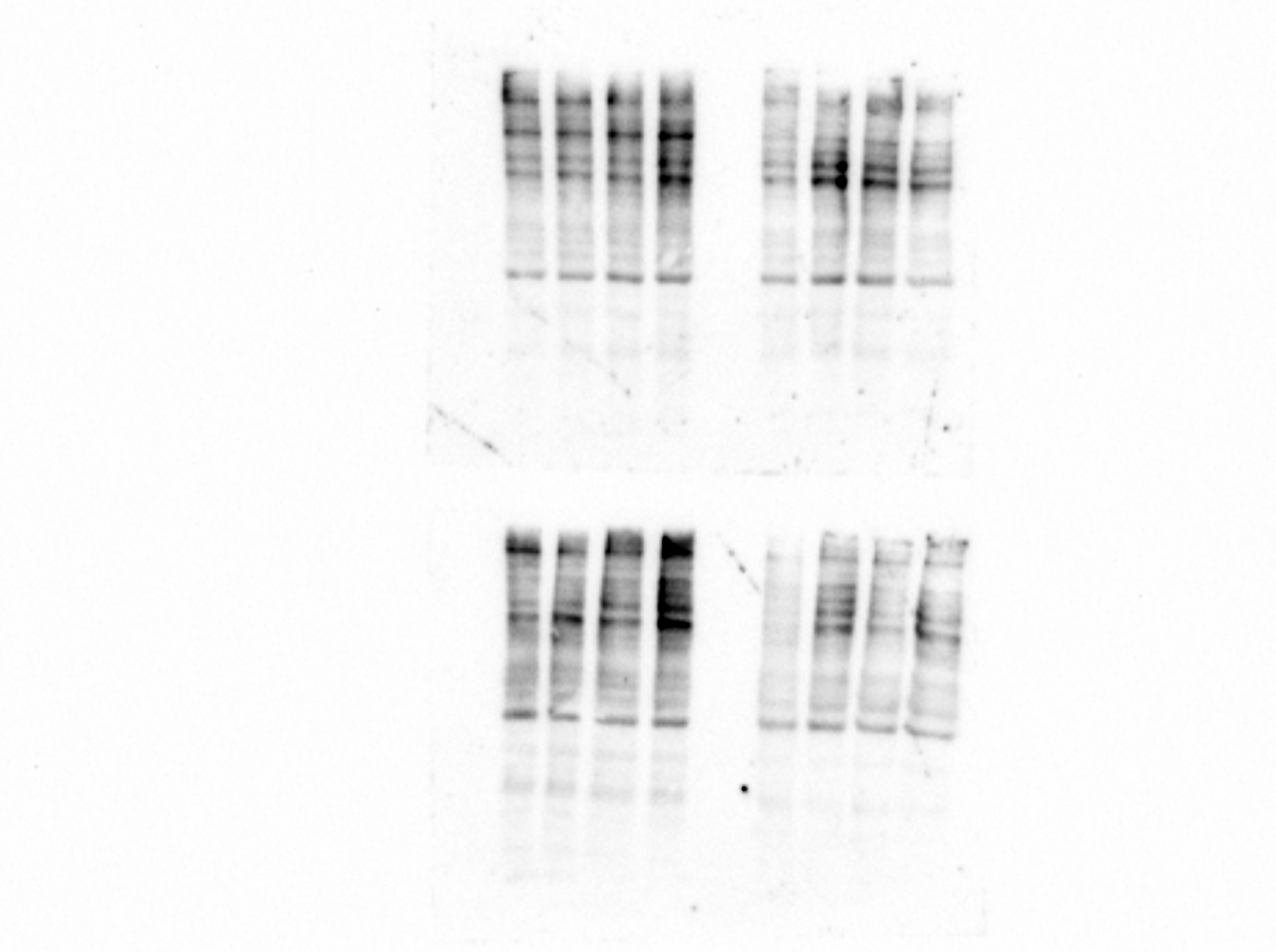

Supplement: Figure 3—figure supplement 1—source data 3. [file elife-80156-fig3-figsupp1-data3.tif]

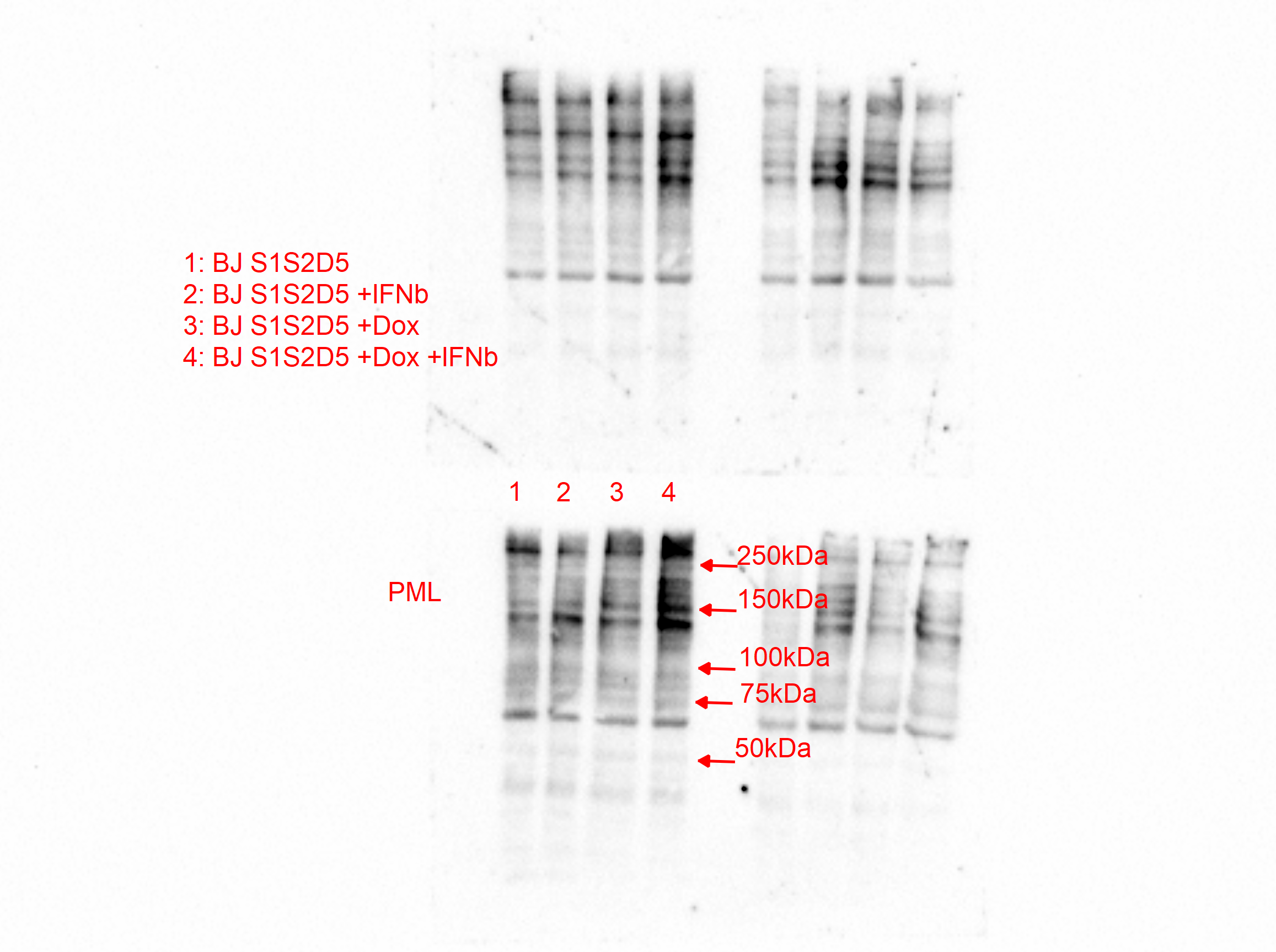

Supplement: Figure 3—figure supplement 1—source data 4. [file elife-80156-fig3-figsupp1-data4.tif]

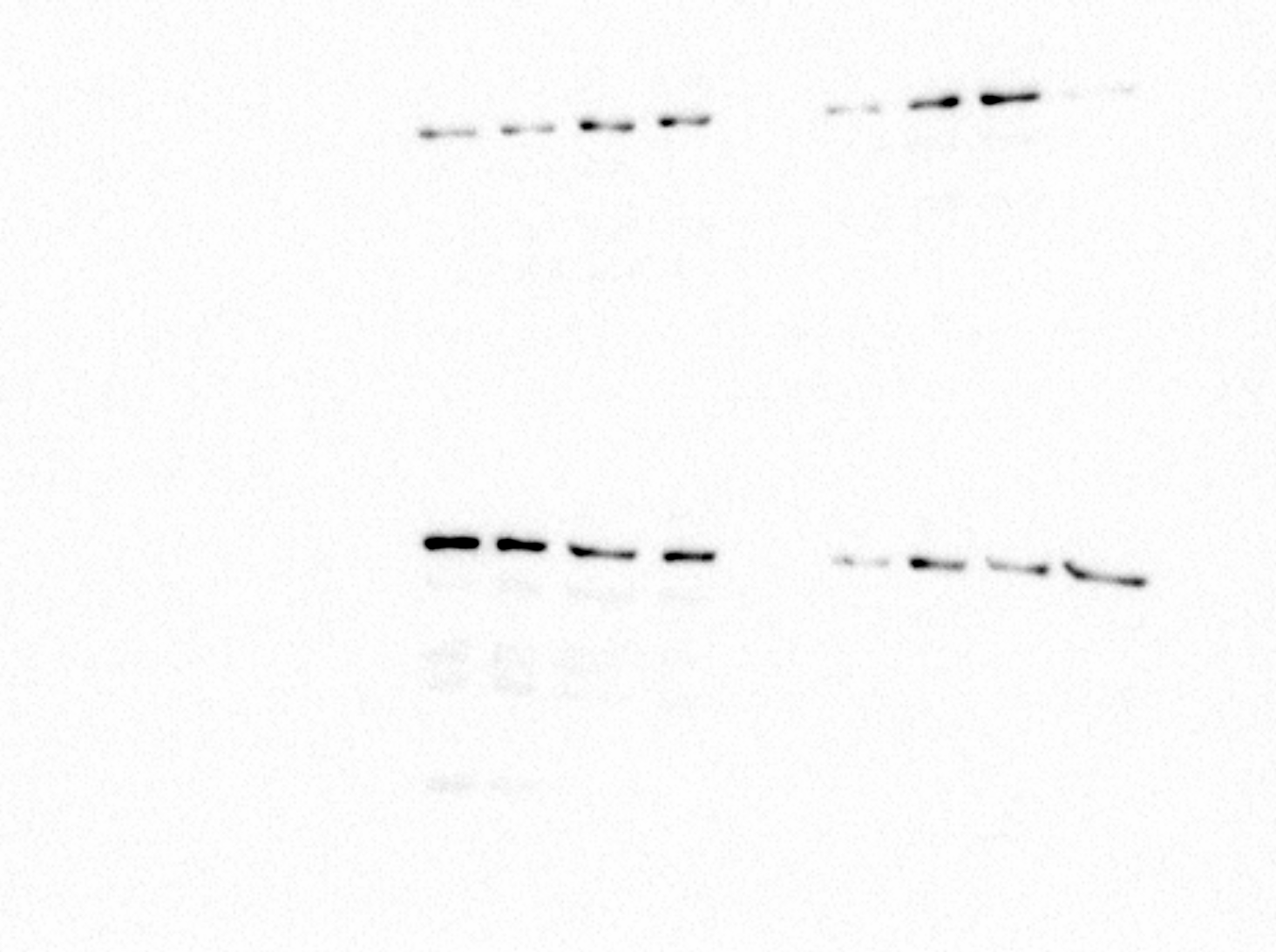

Supplement: Figure 3—figure supplement 1—source data 5. [file elife-80156-fig3-figsupp1-data5.tif]

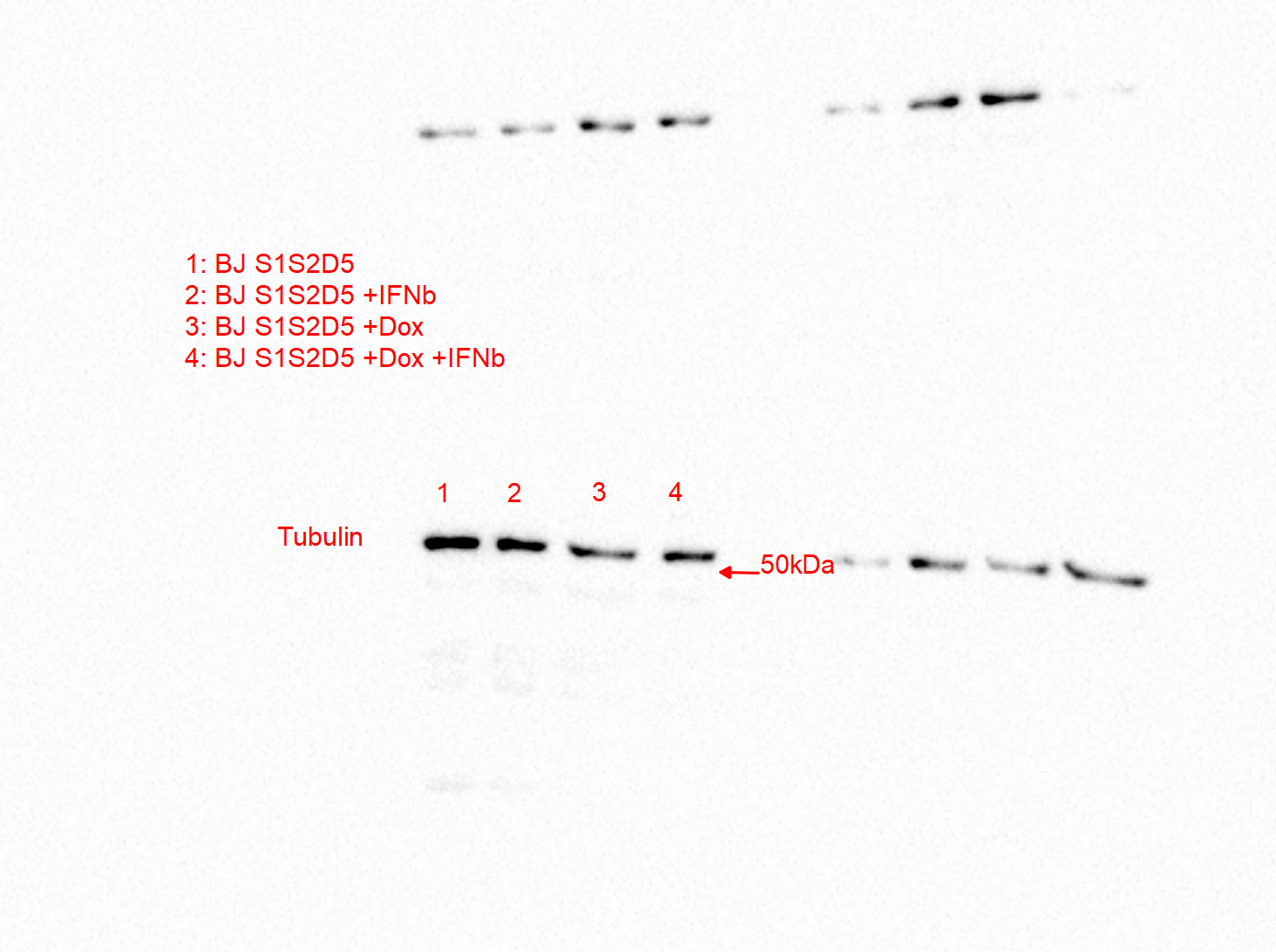

Supplement: Figure 3—figure supplement 1—source data 6. [file elife-80156-fig3-figsupp1-data6.tif]

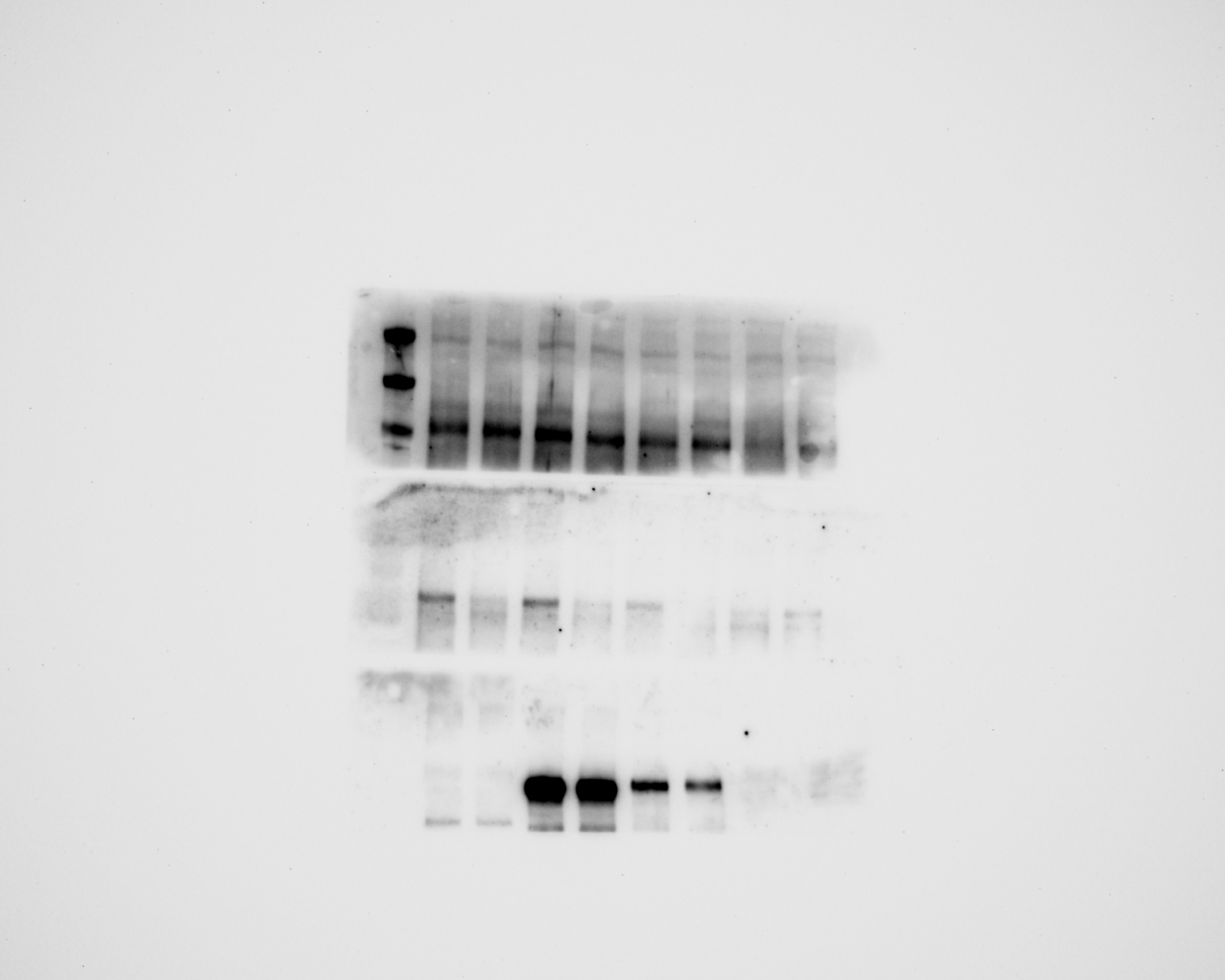

Supplement: Figure 3—figure supplement 1—source data 7. [file elife-80156-fig3-figsupp1-data7.tif]

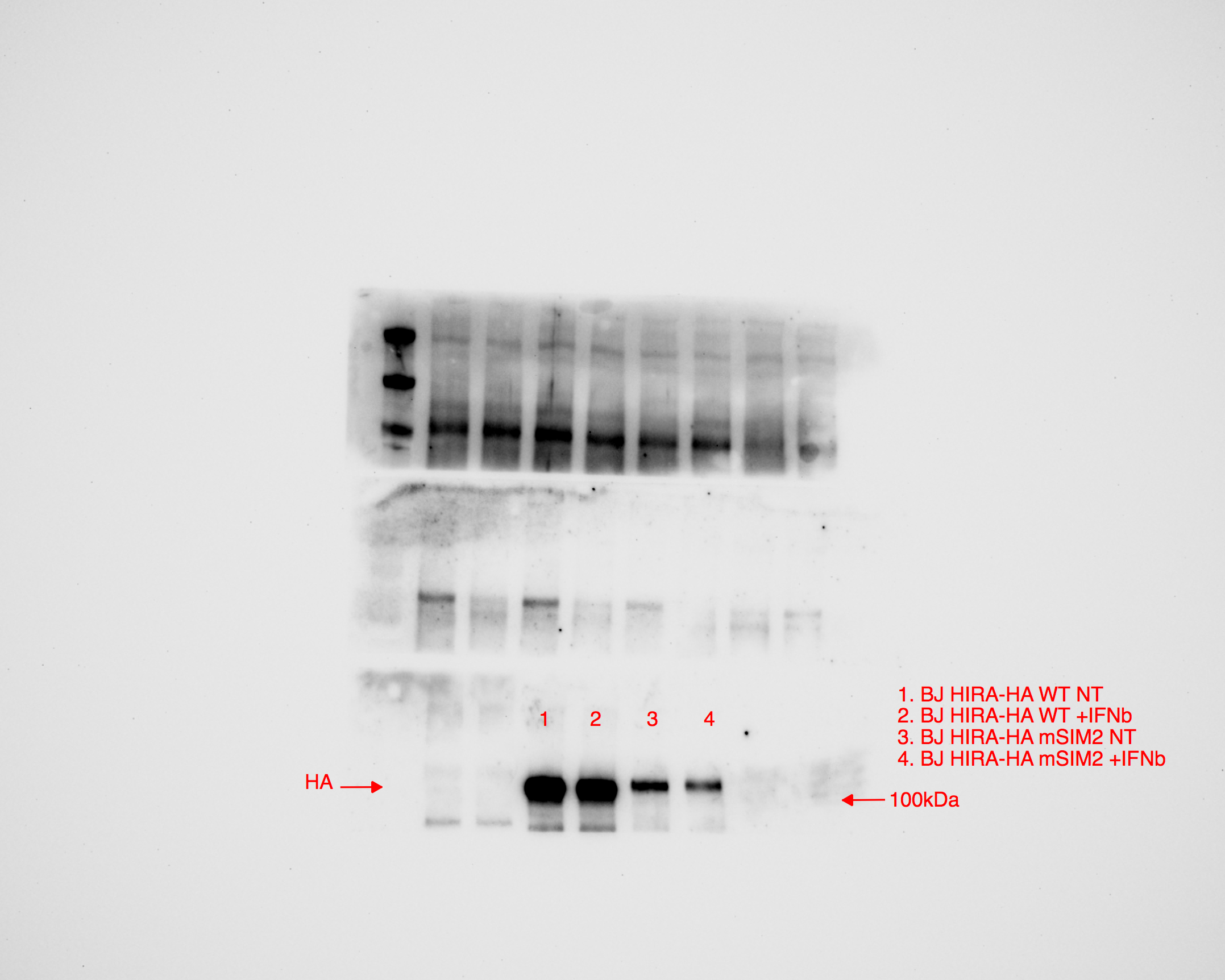

Supplement: Figure 3—figure supplement 1—source data 8. [file elife-80156-fig3-figsupp1-data8.tif]

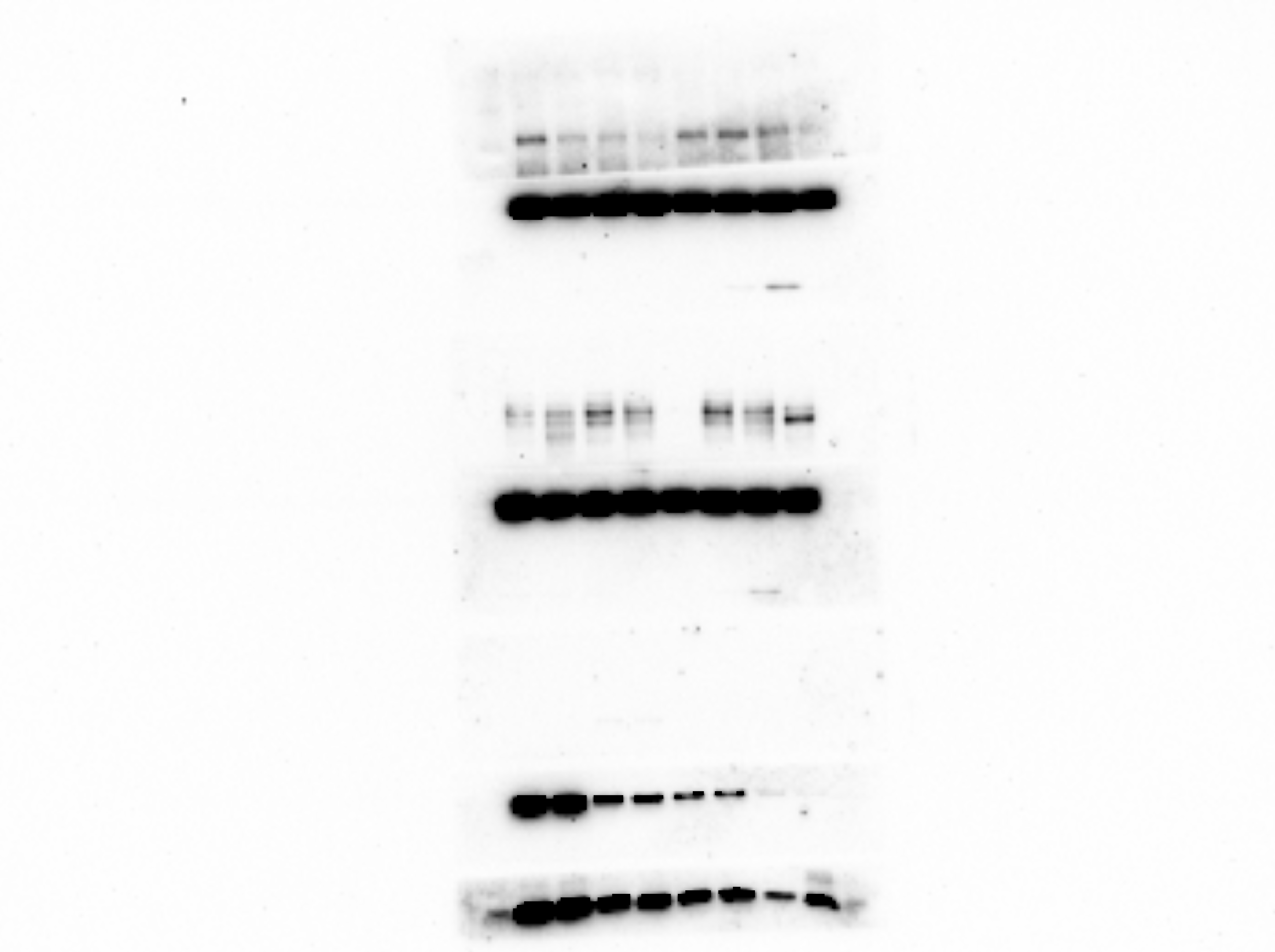

Supplement: Figure 3—figure supplement 1—source data 9. [file elife-80156-fig3-figsupp1-data9.tif]

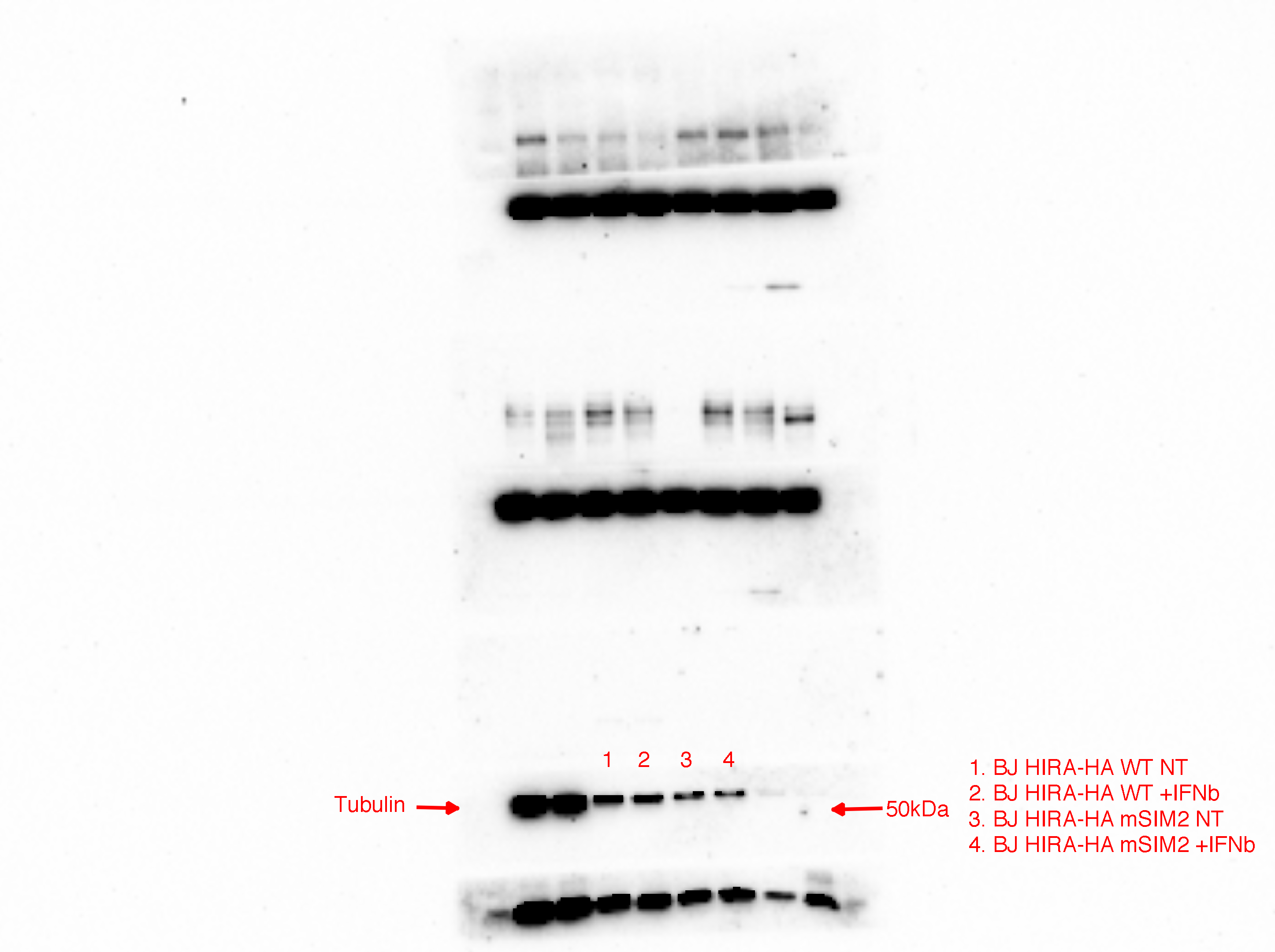

Supplement: Figure 3—figure supplement 1—source data 10. [file elife-80156-fig3-figsupp1-data10.tif]

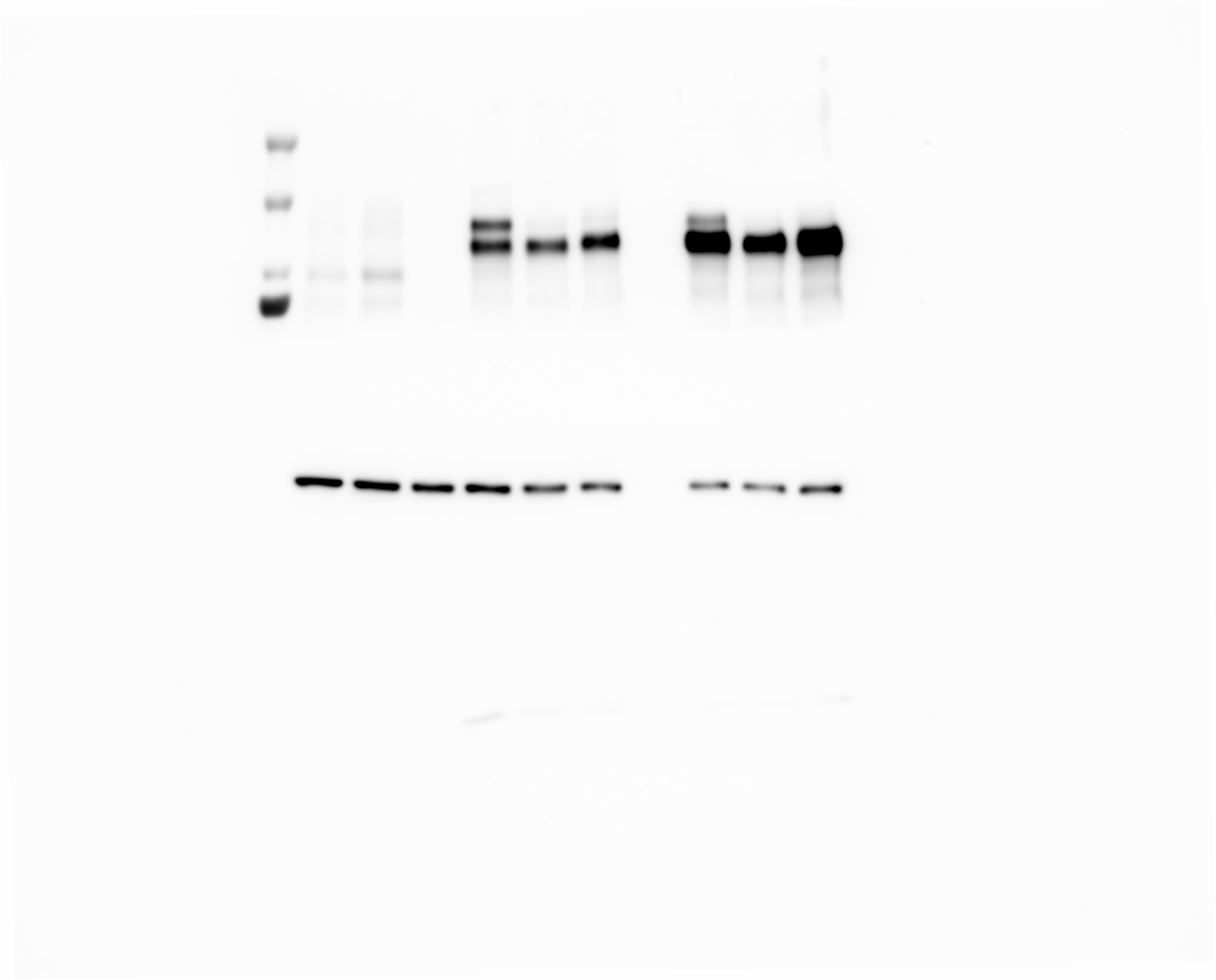

Supplement: Figure 3—figure supplement 2—source data 1. [file elife-80156-fig3-figsupp2-data1.tif]

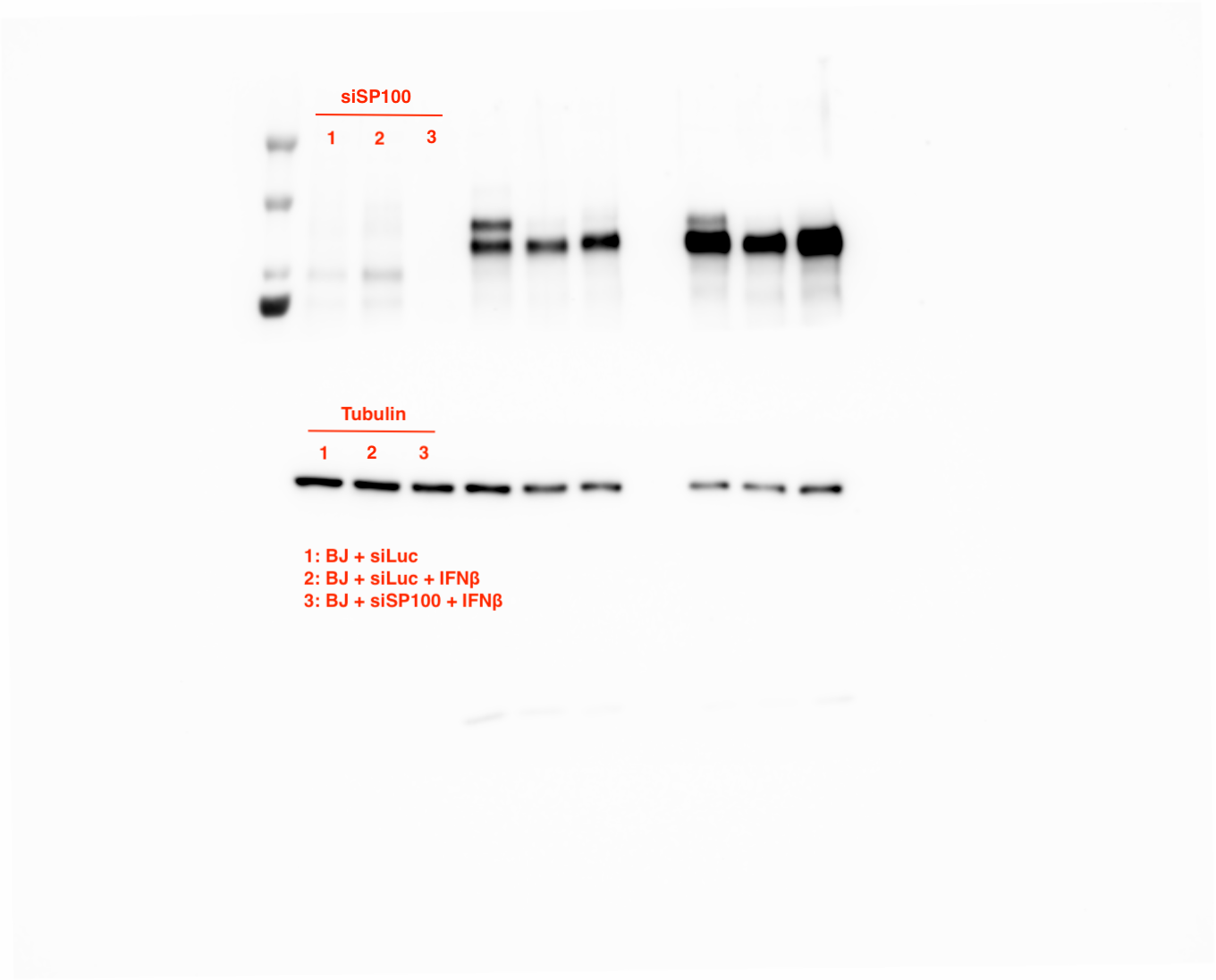

Supplement: Figure 3—figure supplement 2—source data 2. [file elife-80156-fig3-figsupp2-data2.tif]

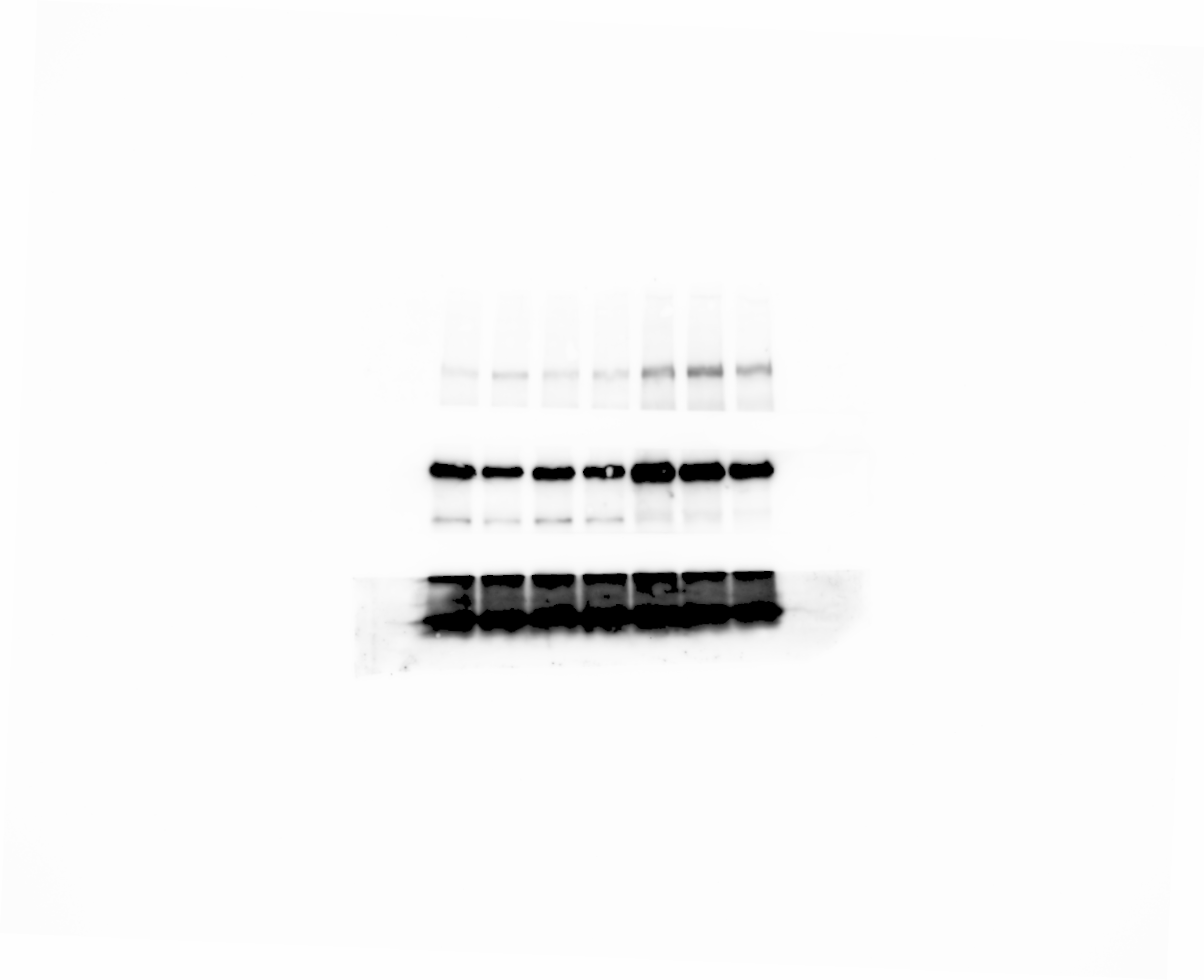

Supplement: Figure 3—figure supplement 2—source data 3. [file elife-80156-fig3-figsupp2-data3.tif]

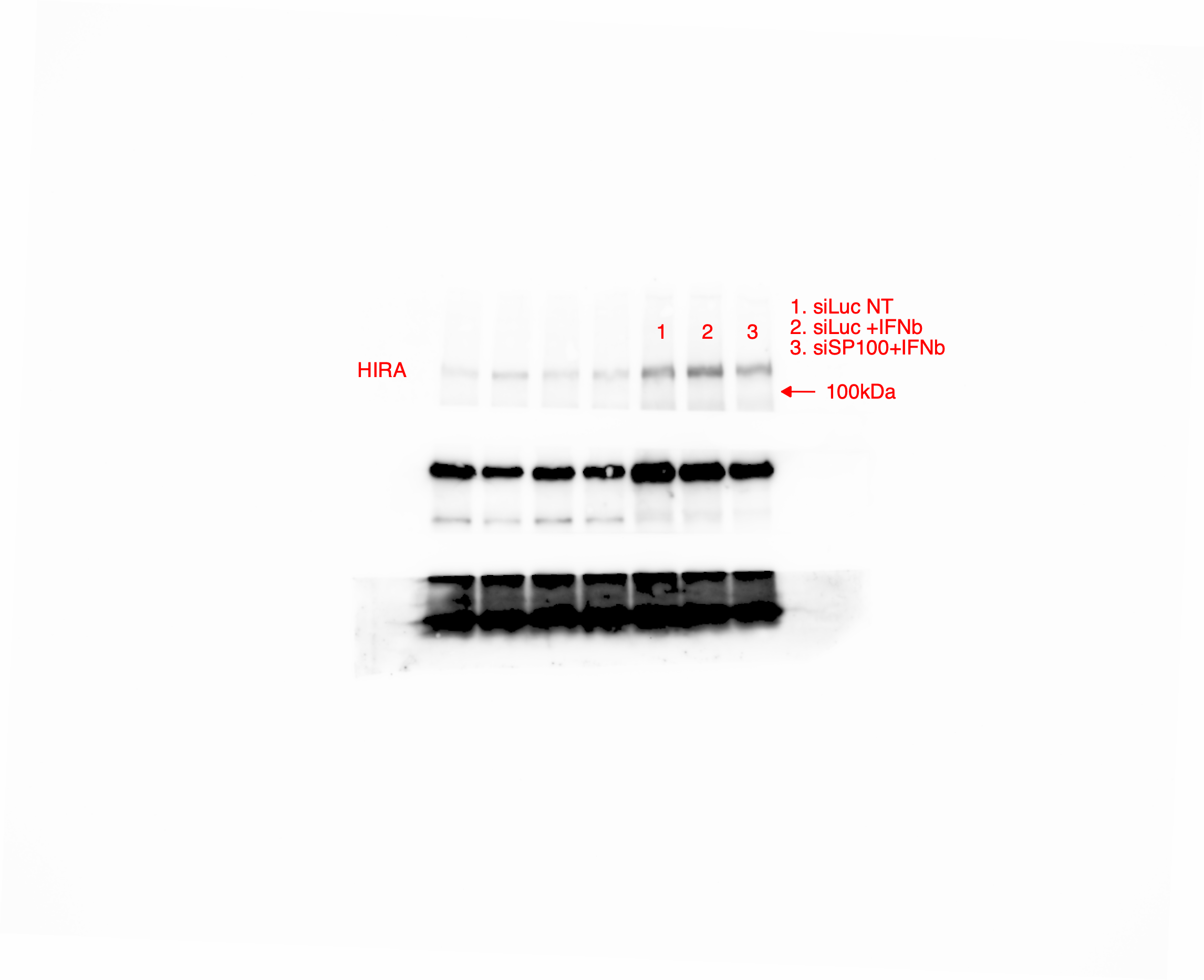

Supplement: Figure 3—figure supplement 2—source data 4. [file elife-80156-fig3-figsupp2-data4.tif]

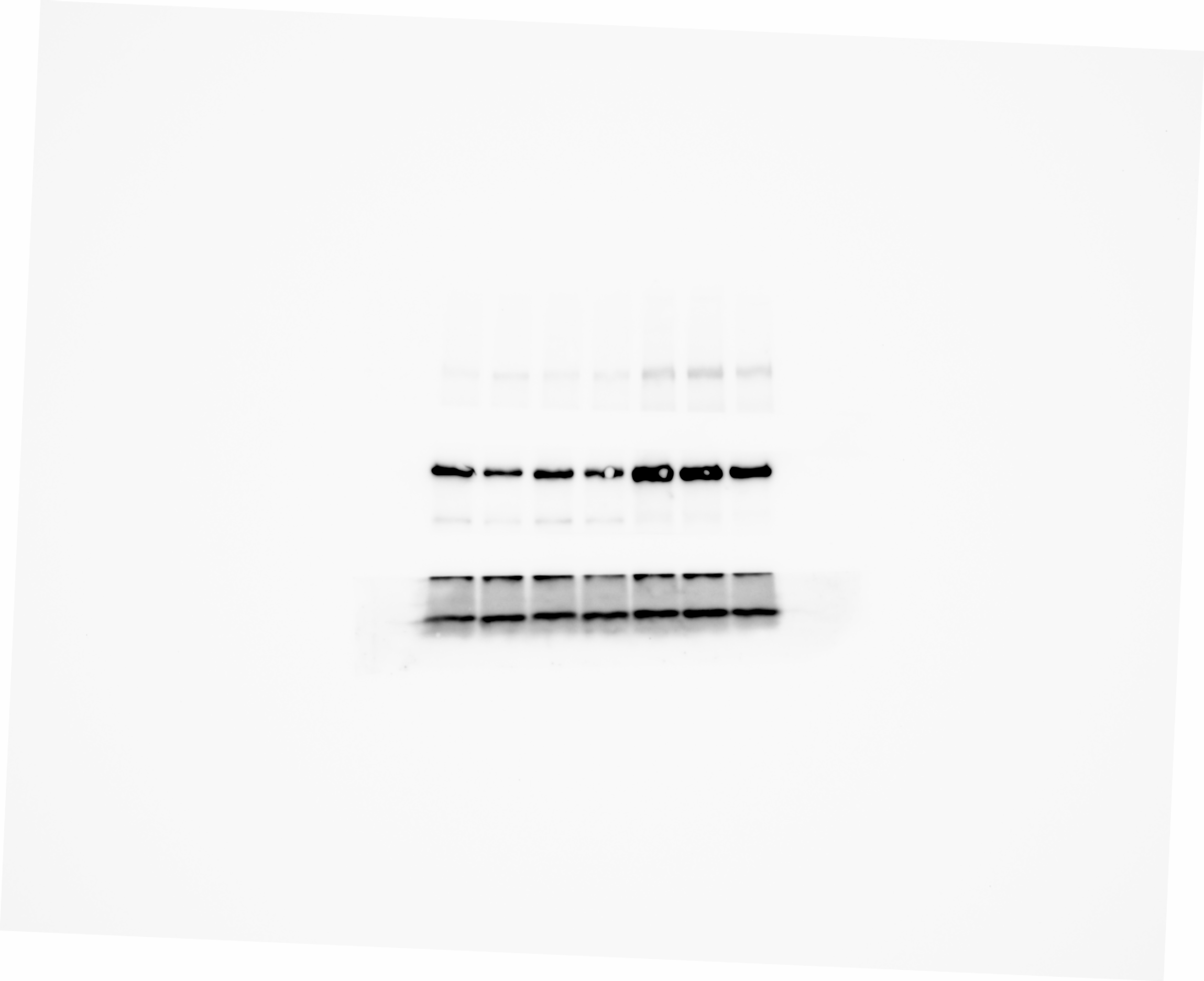

Supplement: Figure 3—figure supplement 2—source data 5. [file elife-80156-fig3-figsupp2-data5.tif]

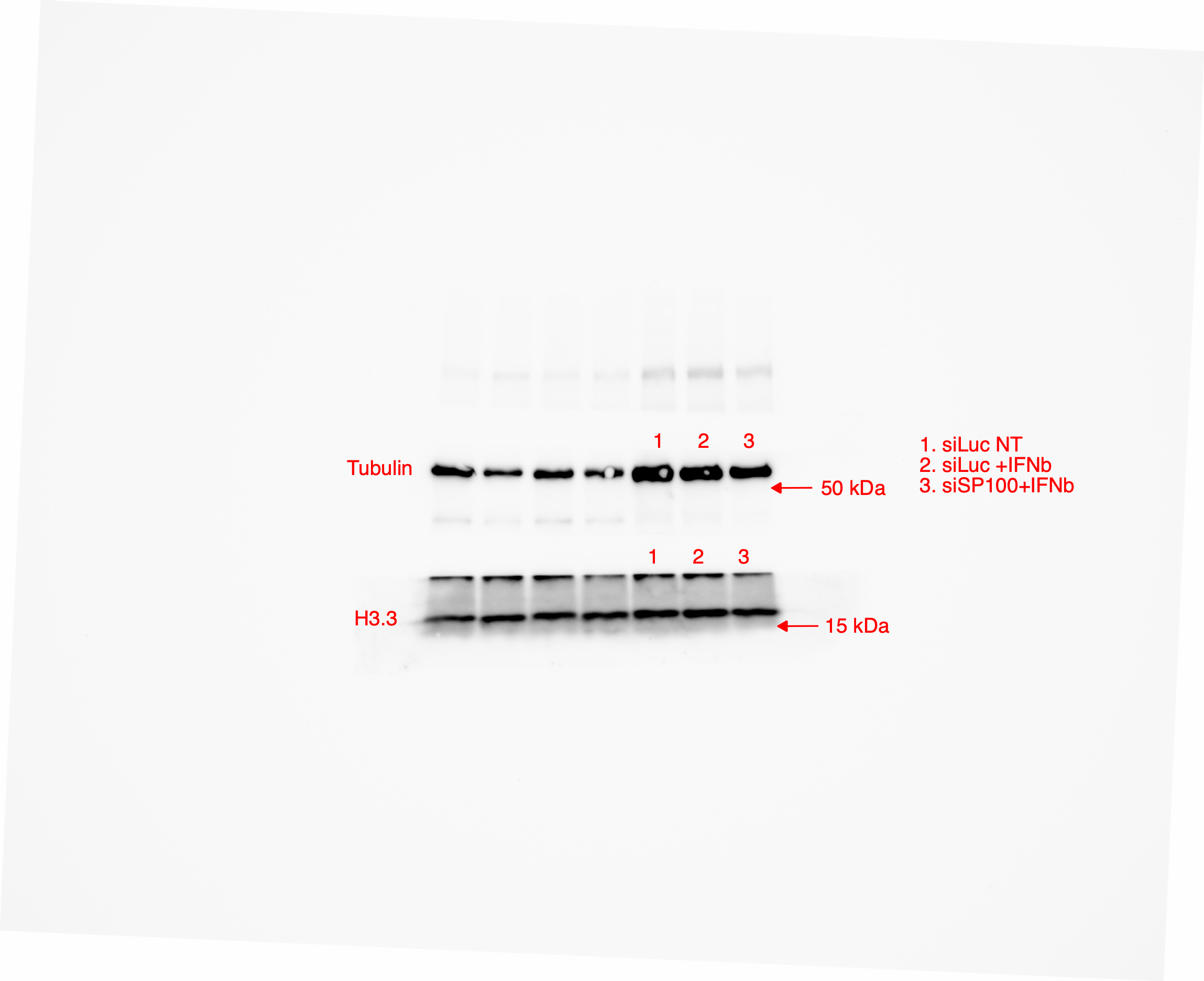

Supplement: Figure 3—figure supplement 2—source data 6. [file elife-80156-fig3-figsupp2-data6.tif]

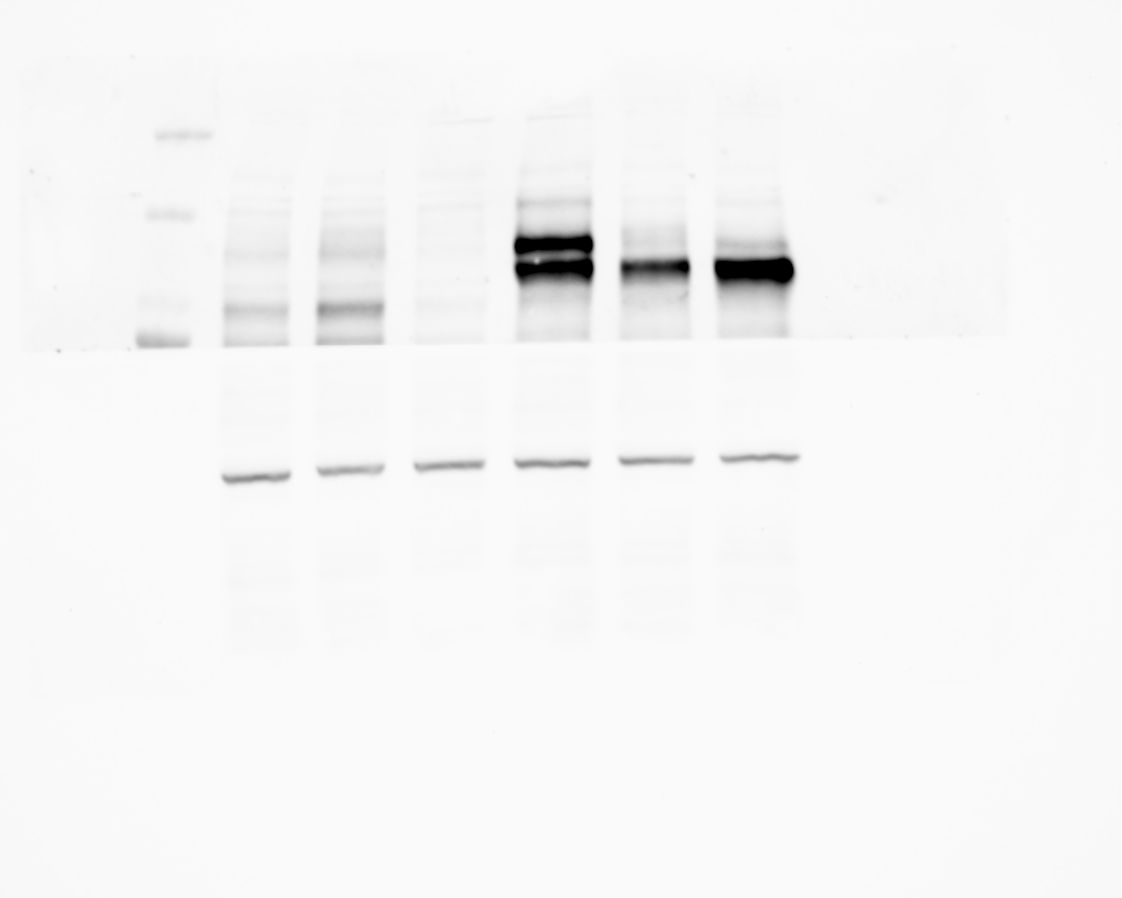

Supplement: Figure 3—figure supplement 2—source data 7. [file elife-80156-fig3-figsupp2-data7.tif]

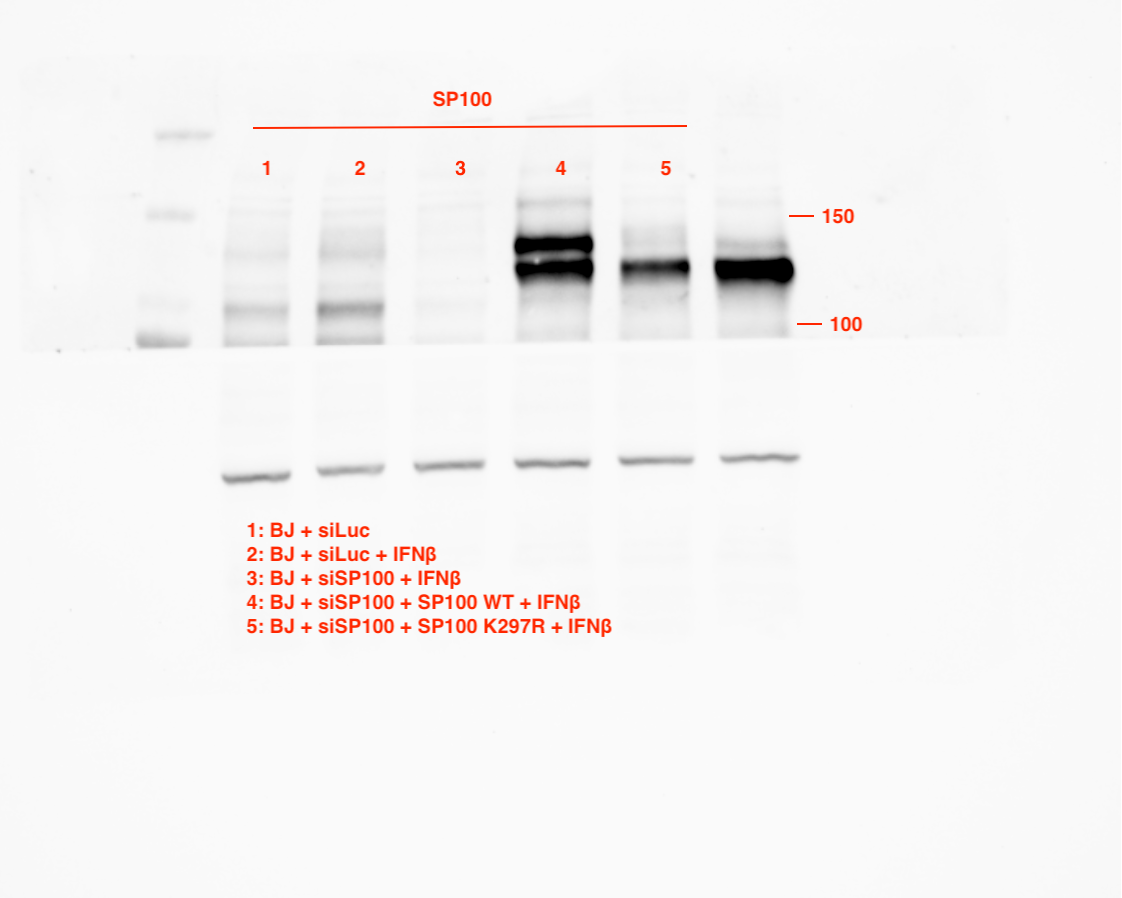

Supplement: Figure 3—figure supplement 2—source data 8. [file elife-80156-fig3-figsupp2-data8.tif]

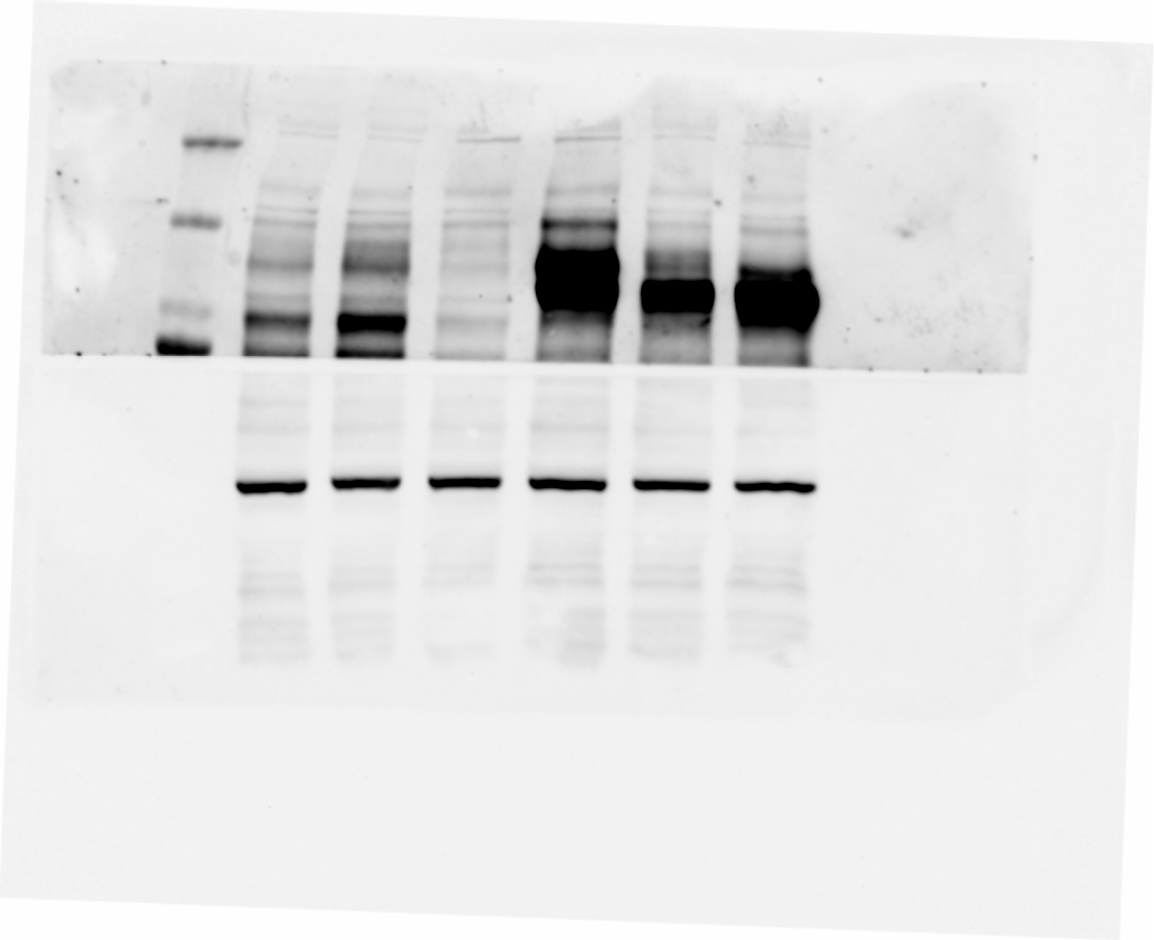

Supplement: Figure 3—figure supplement 2—source data 9. [file elife-80156-fig3-figsupp2-data9.tif]

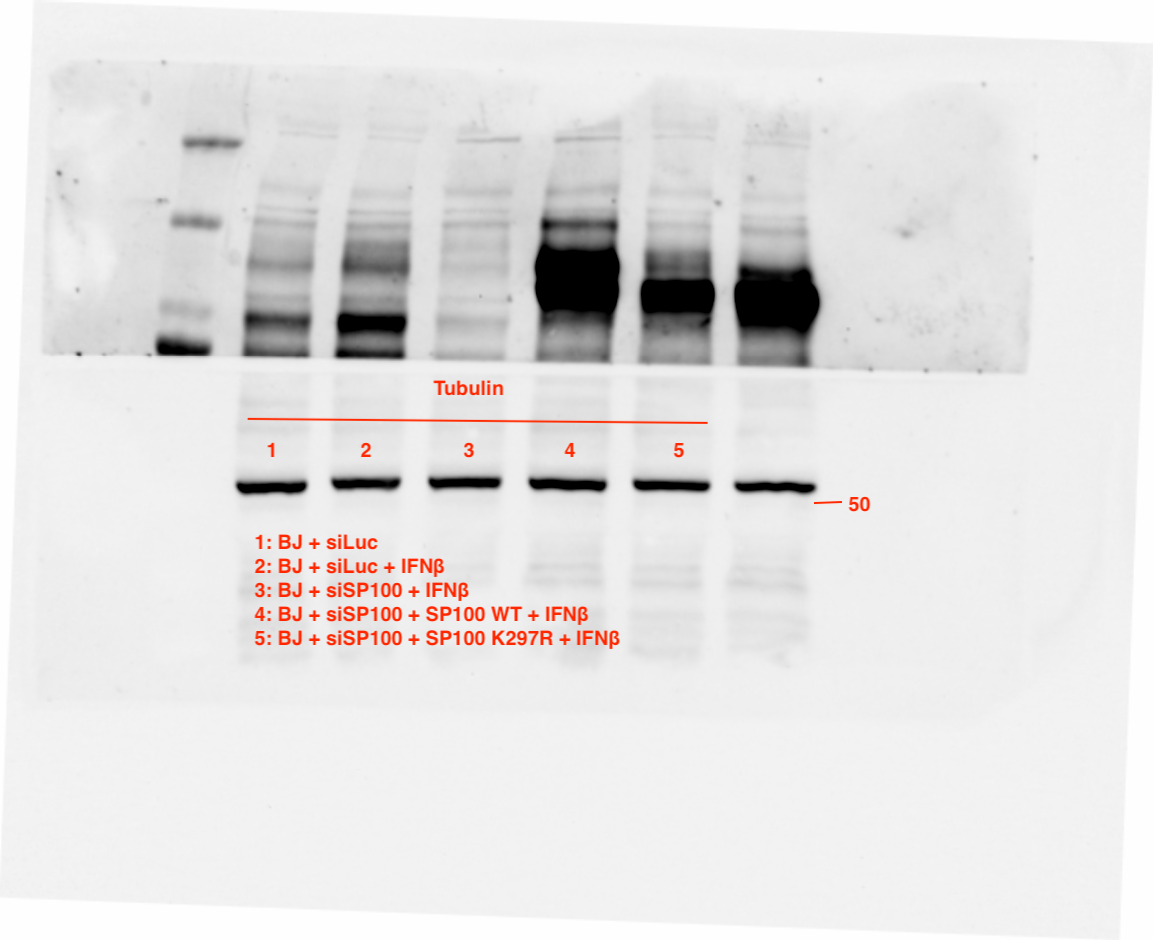

Supplement: Figure 3—figure supplement 2—source data 10. [file elife-80156-fig3-figsupp2-data10.tif]

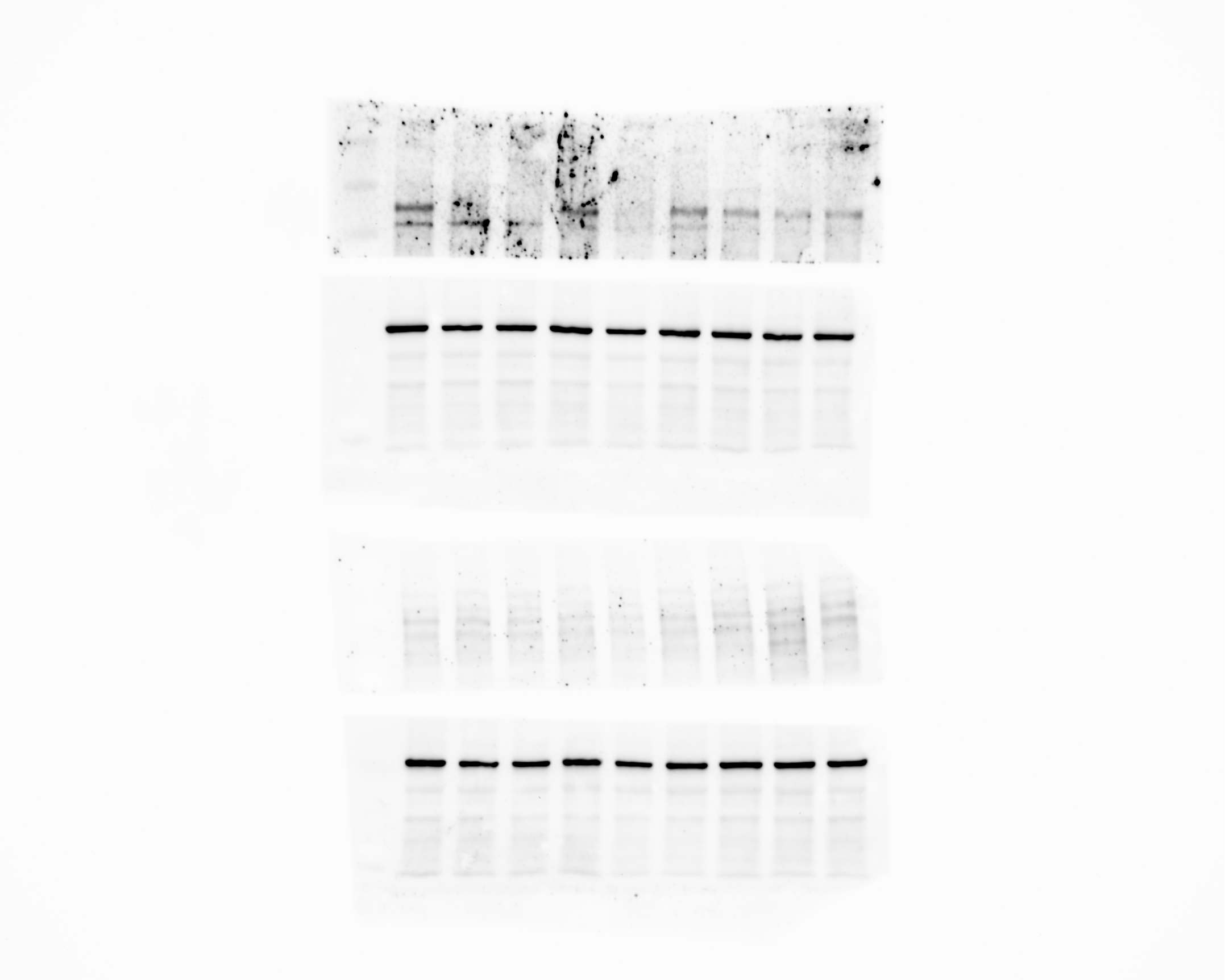

Supplement: Figure 4—figure supplement 1—source data 1. [file elife-80156-fig4-figsupp1-data1.tif]

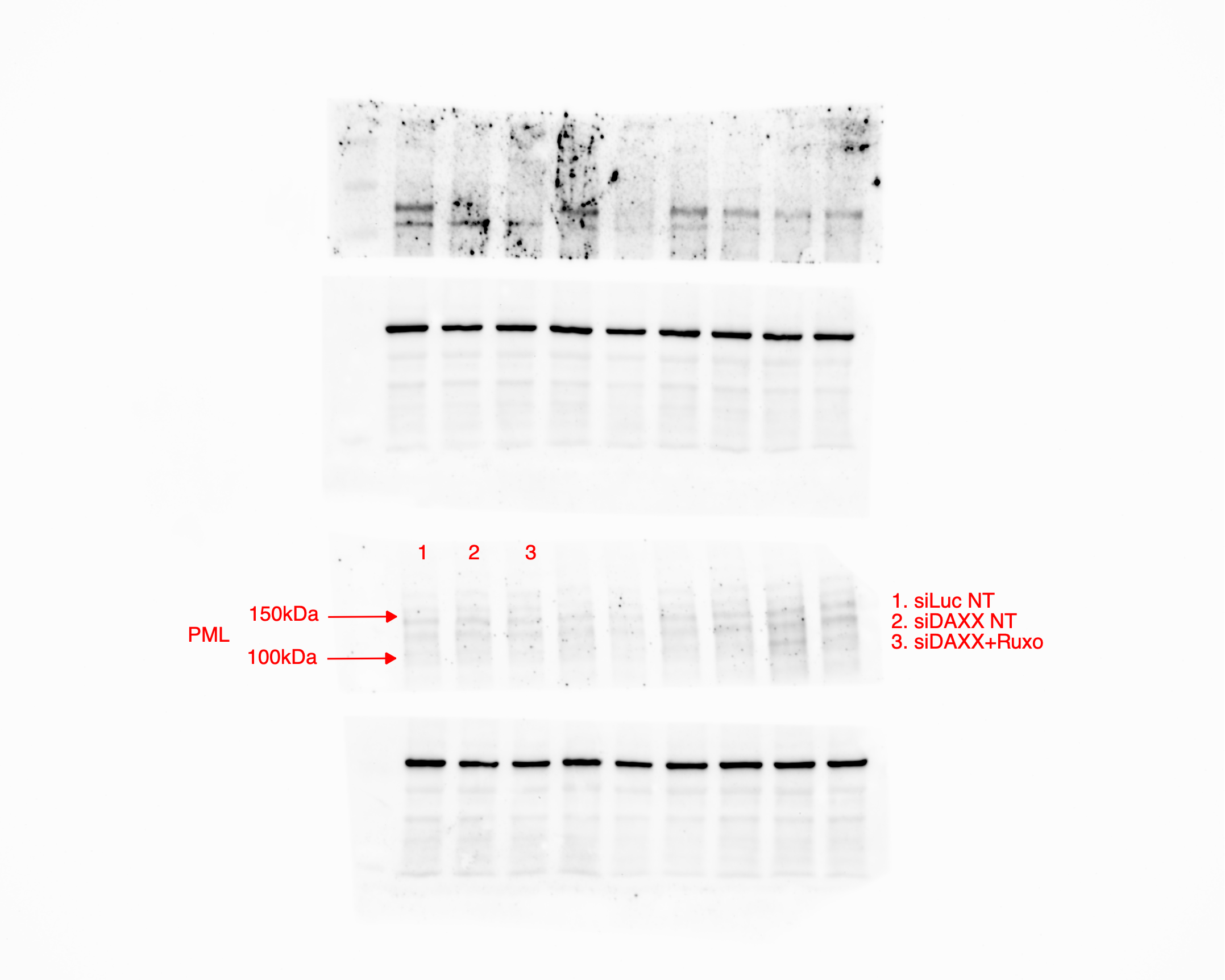

Supplement: Figure 4—figure supplement 1—source data 2. [file elife-80156-fig4-figsupp1-data2.tif]

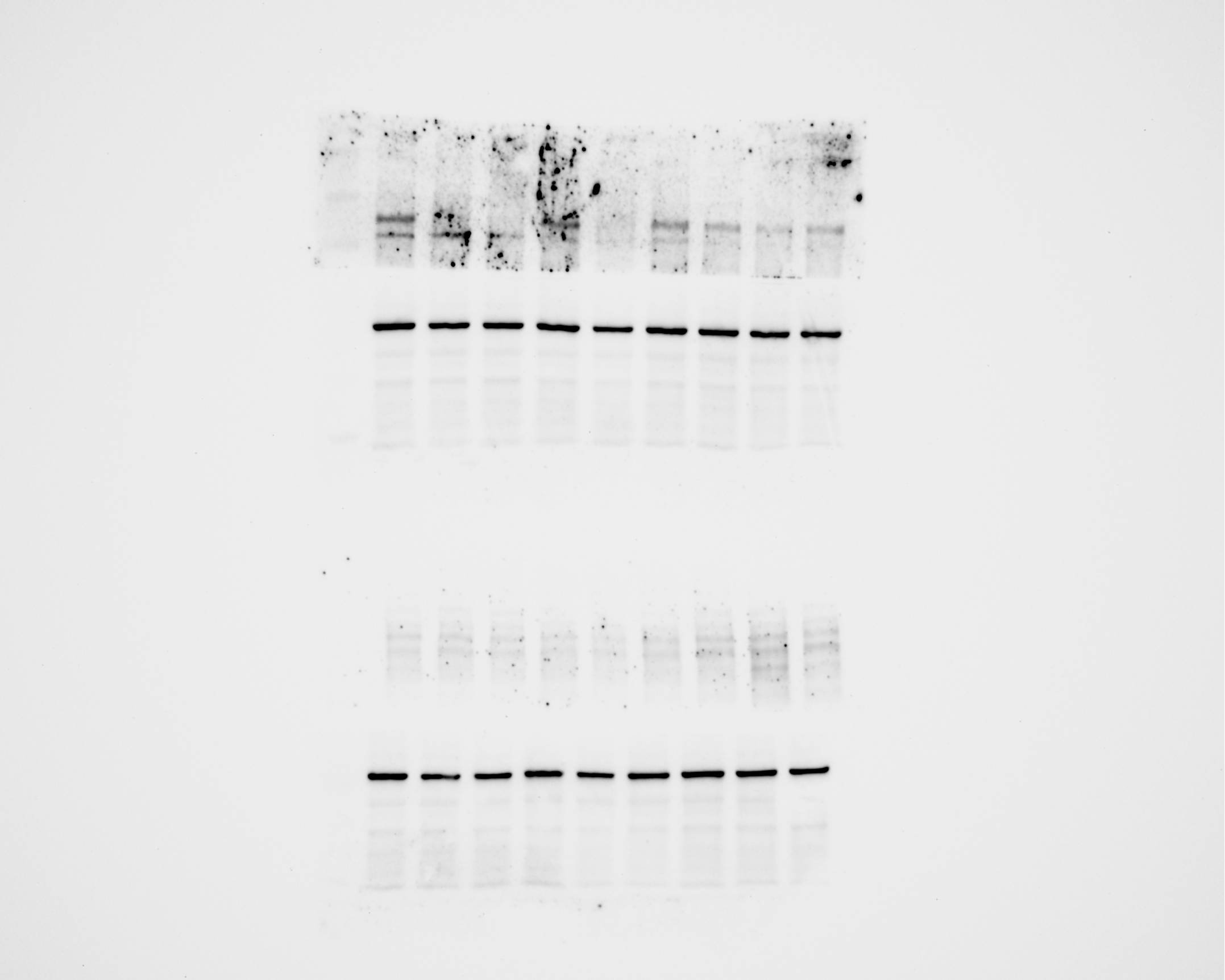

Supplement: Figure 4—figure supplement 1—source data 3. [file elife-80156-fig4-figsupp1-data3.tif]

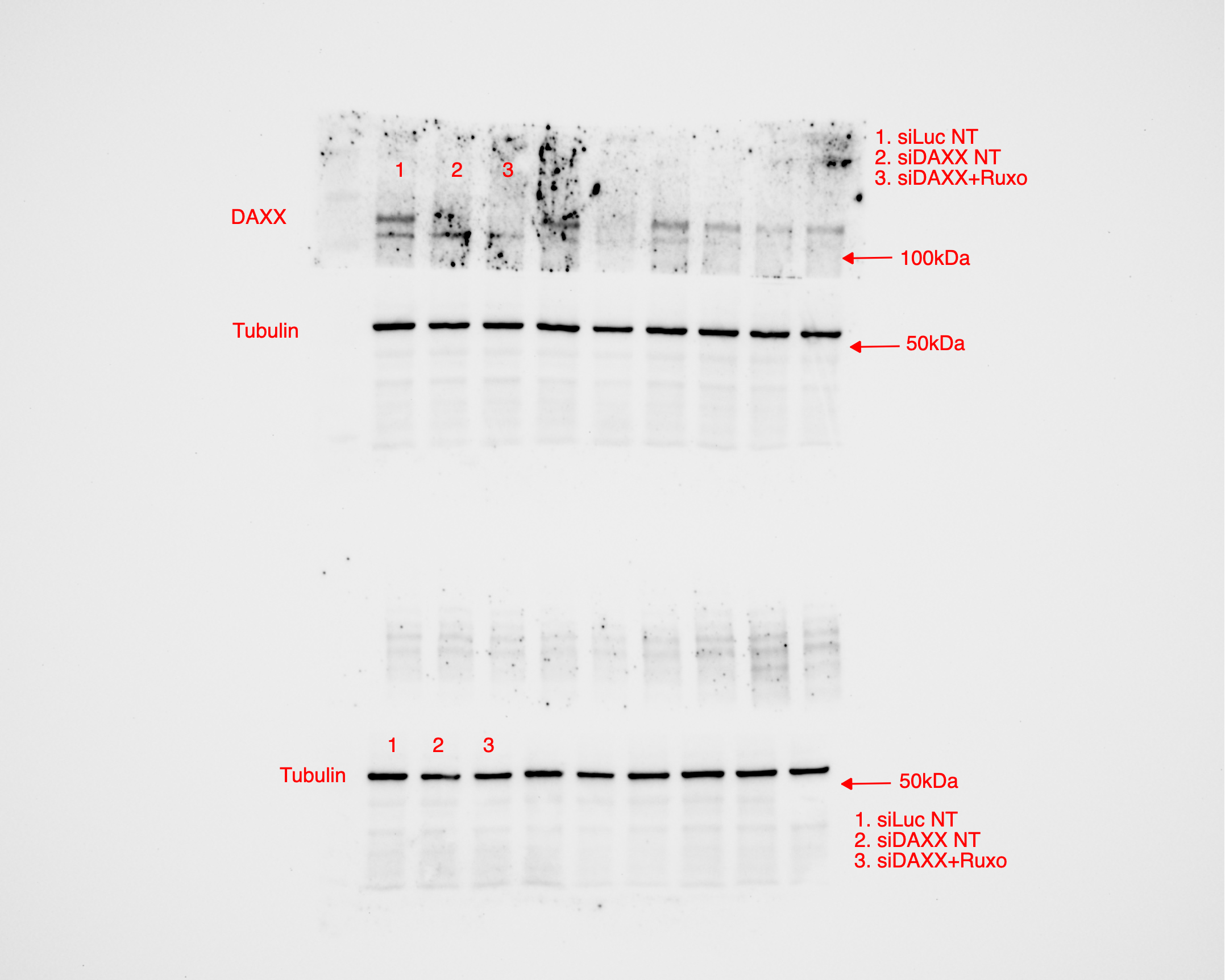

Supplement: Figure 4—figure supplement 1—source data 4. [file elife-80156-fig4-figsupp1-data4.tif]

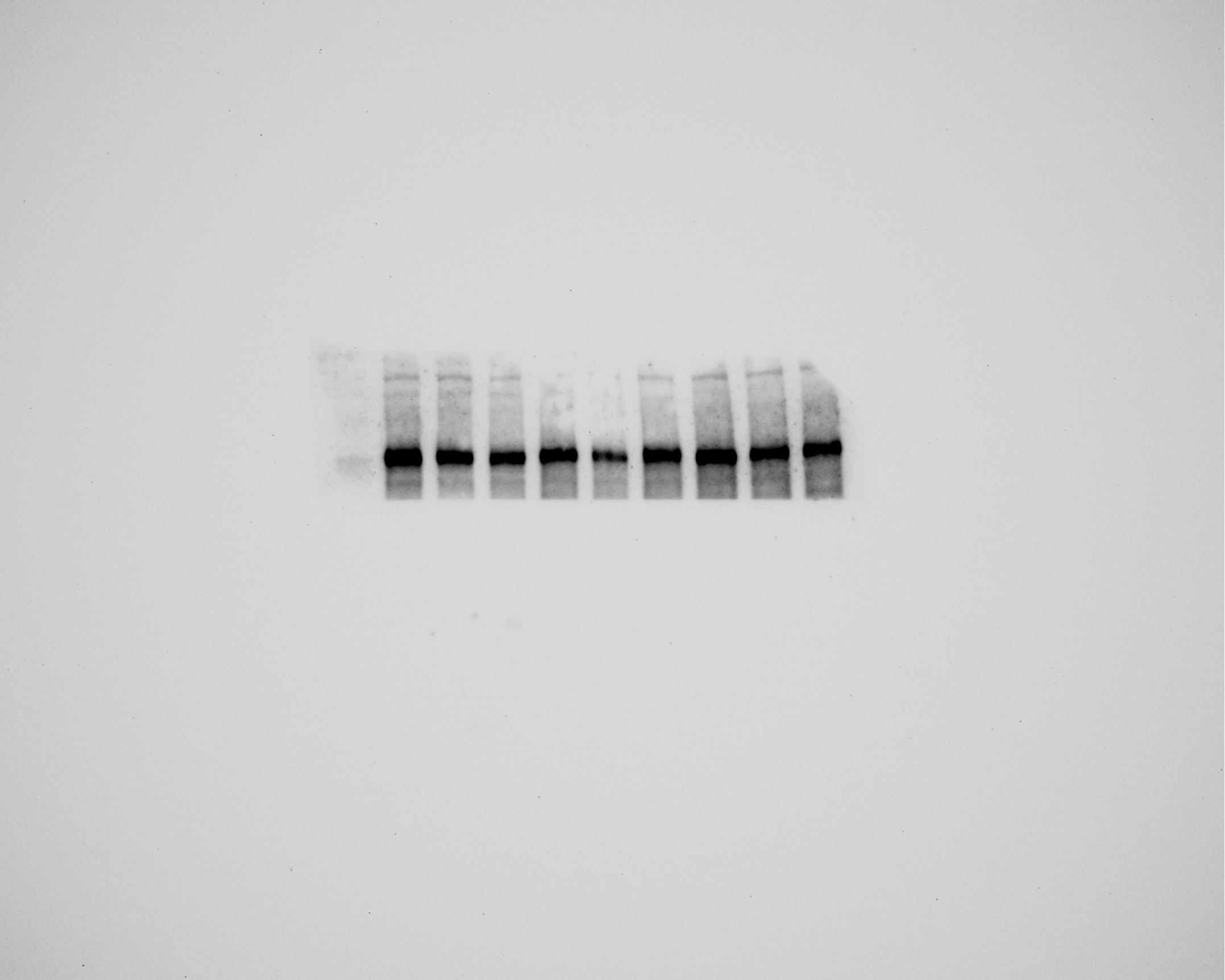

Supplement: Figure 4—figure supplement 1—source data 5. [file elife-80156-fig4-figsupp1-data5.tif]

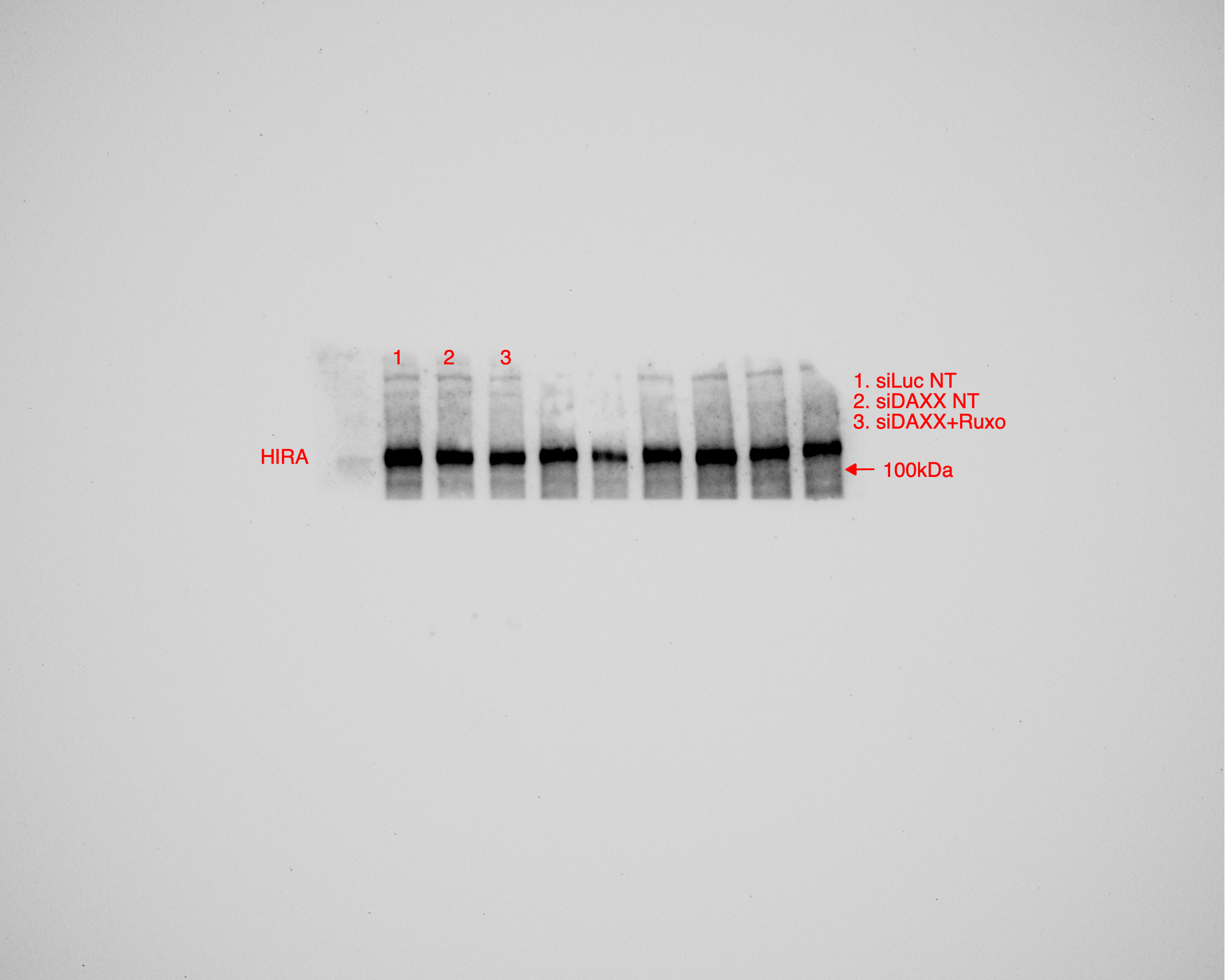

Supplement: Figure 4—figure supplement 1—source data 6. [file elife-80156-fig4-figsupp1-data6.tif]

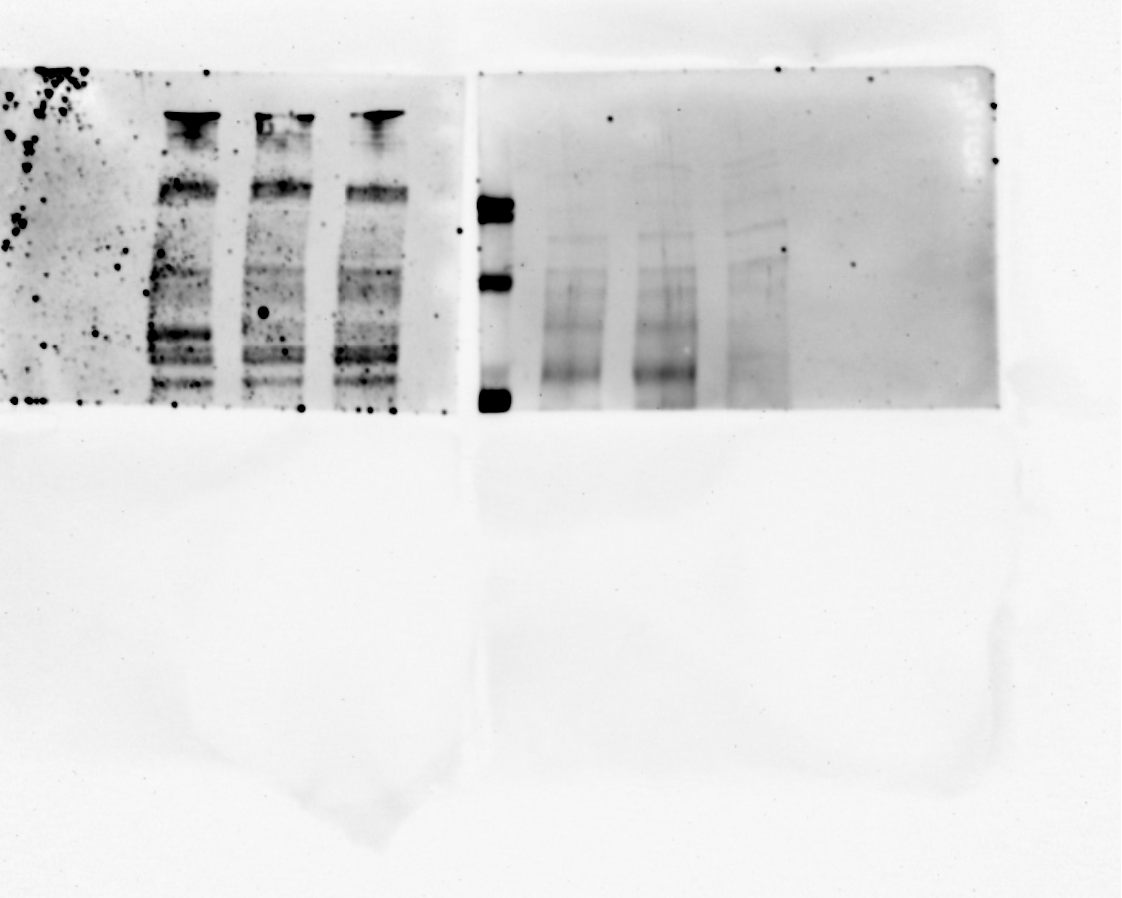

Supplement: Figure 4—figure supplement 1—source data 7. [file elife-80156-fig4-figsupp1-data7.tif]

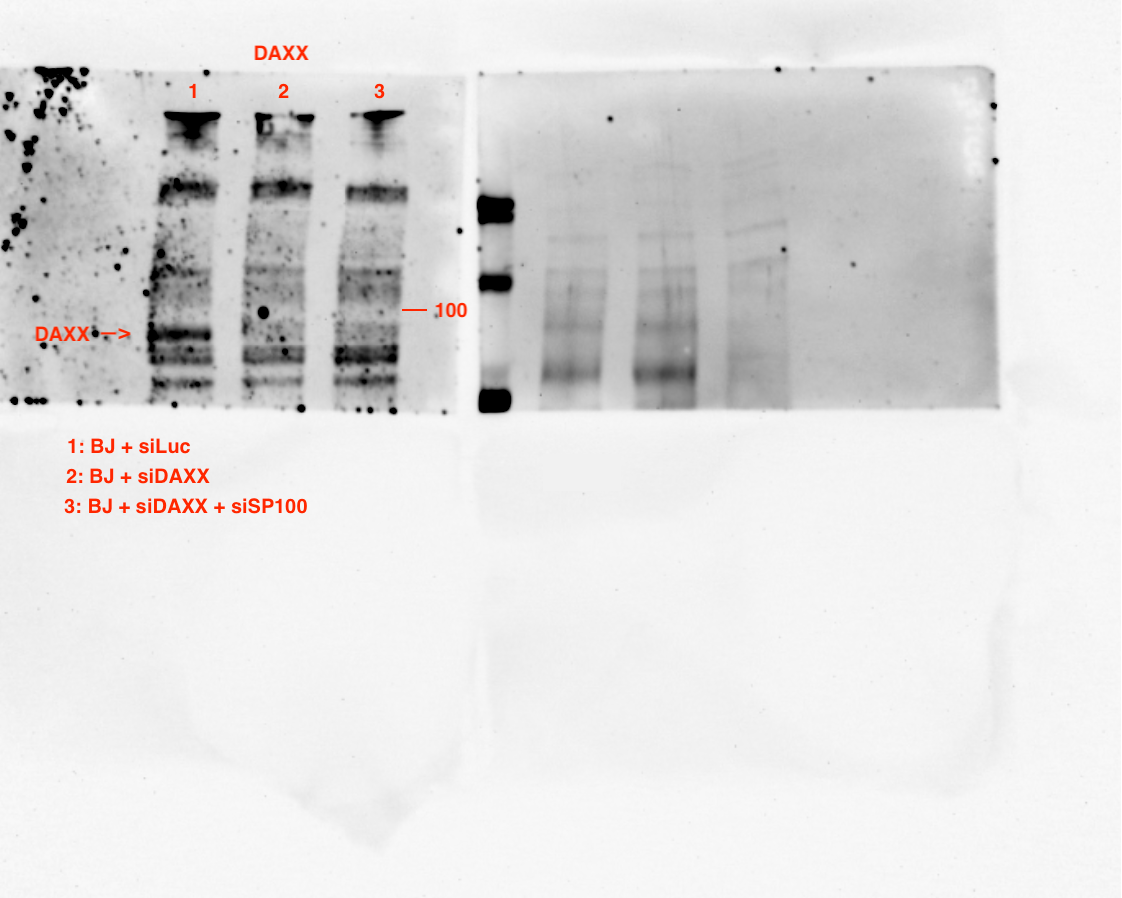

Supplement: Figure 4—figure supplement 1—source data 8. [file elife-80156-fig4-figsupp1-data8.tif]

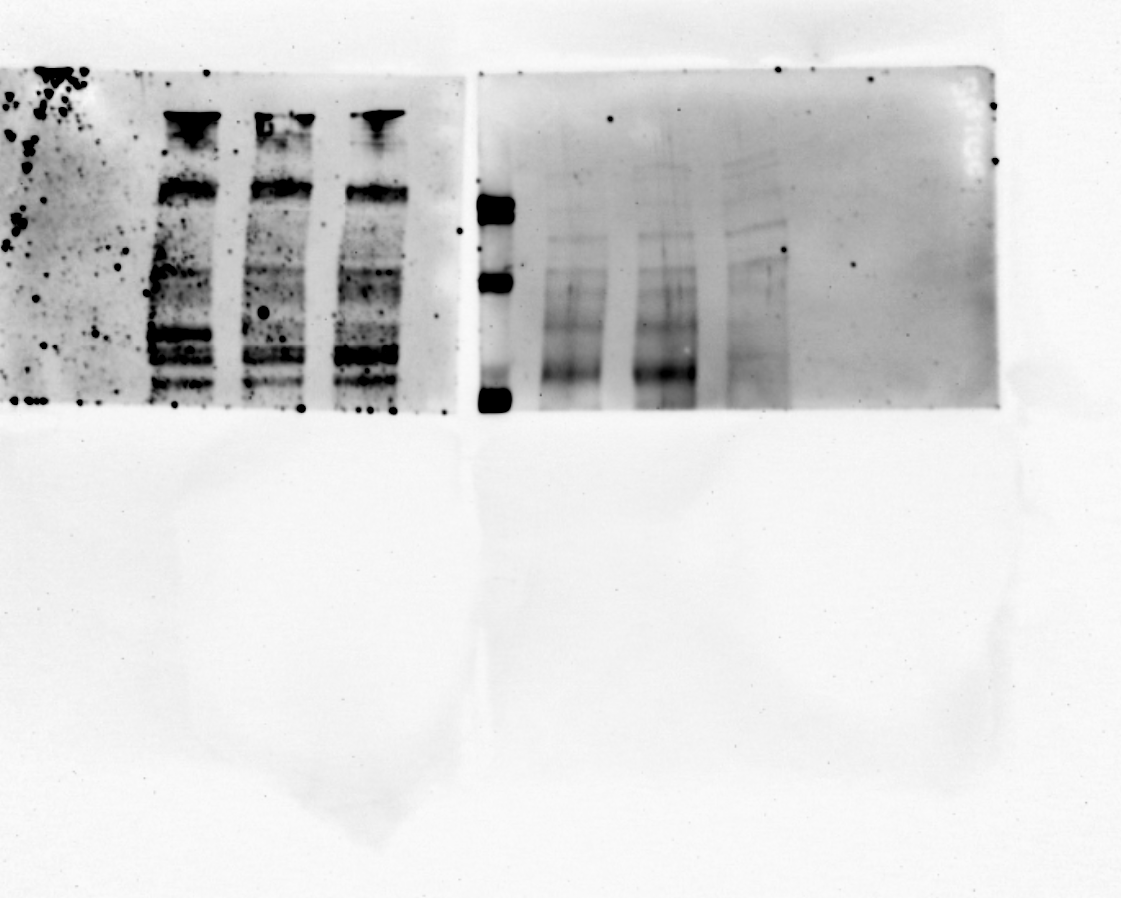

Supplement: Figure 4—figure supplement 1—source data 9. [file elife-80156-fig4-figsupp1-data9.tif]

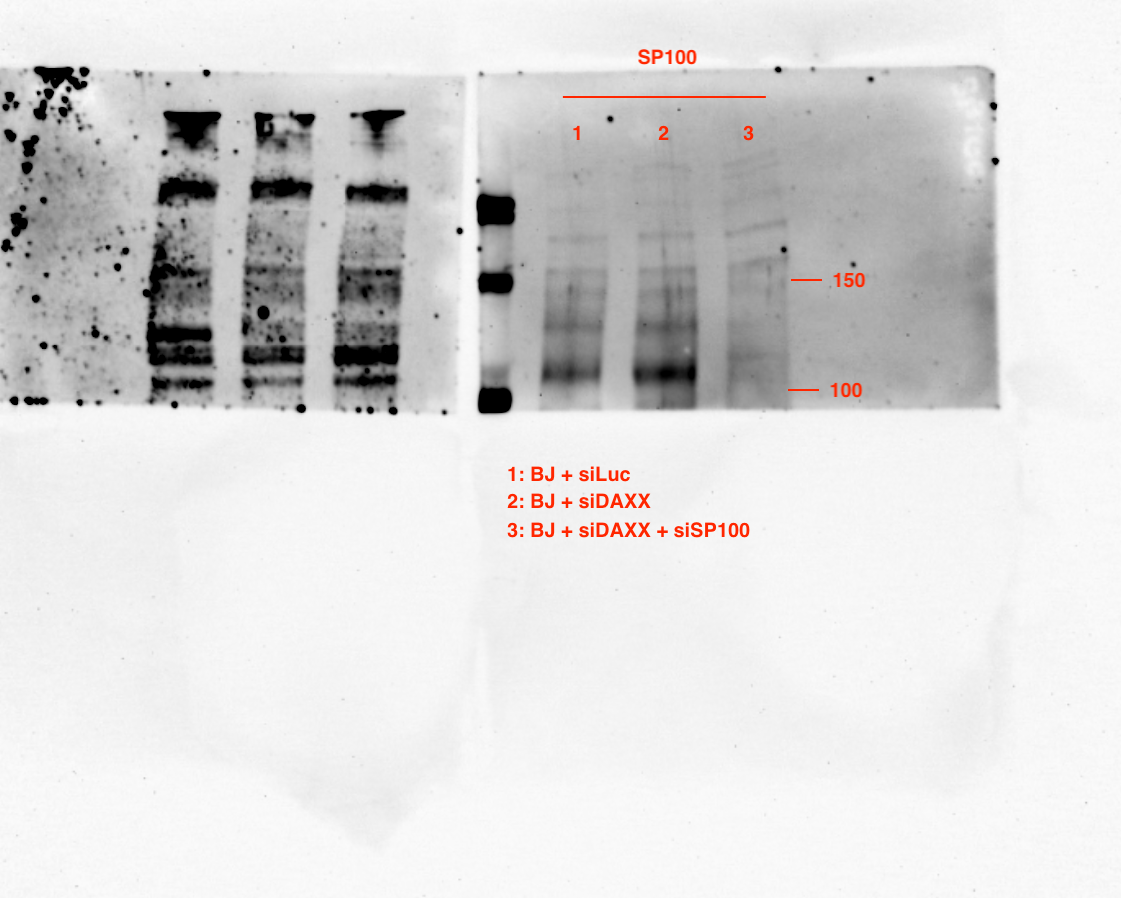

Supplement: Figure 4—figure supplement 1—source data 10. [file elife-80156-fig4-figsupp1-data10.tif]

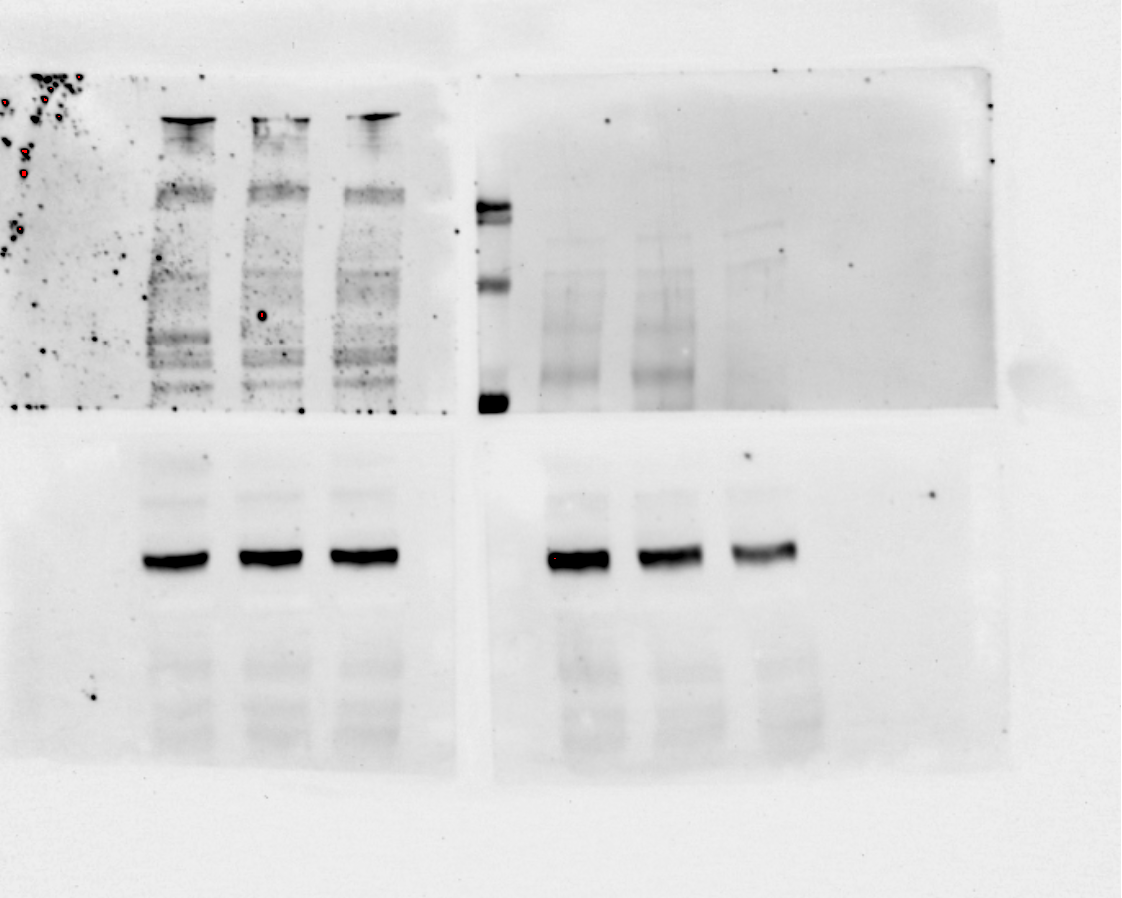

Supplement: Figure 4—figure supplement 1—source data 11. [file elife-80156-fig4-figsupp1-data11.tif]

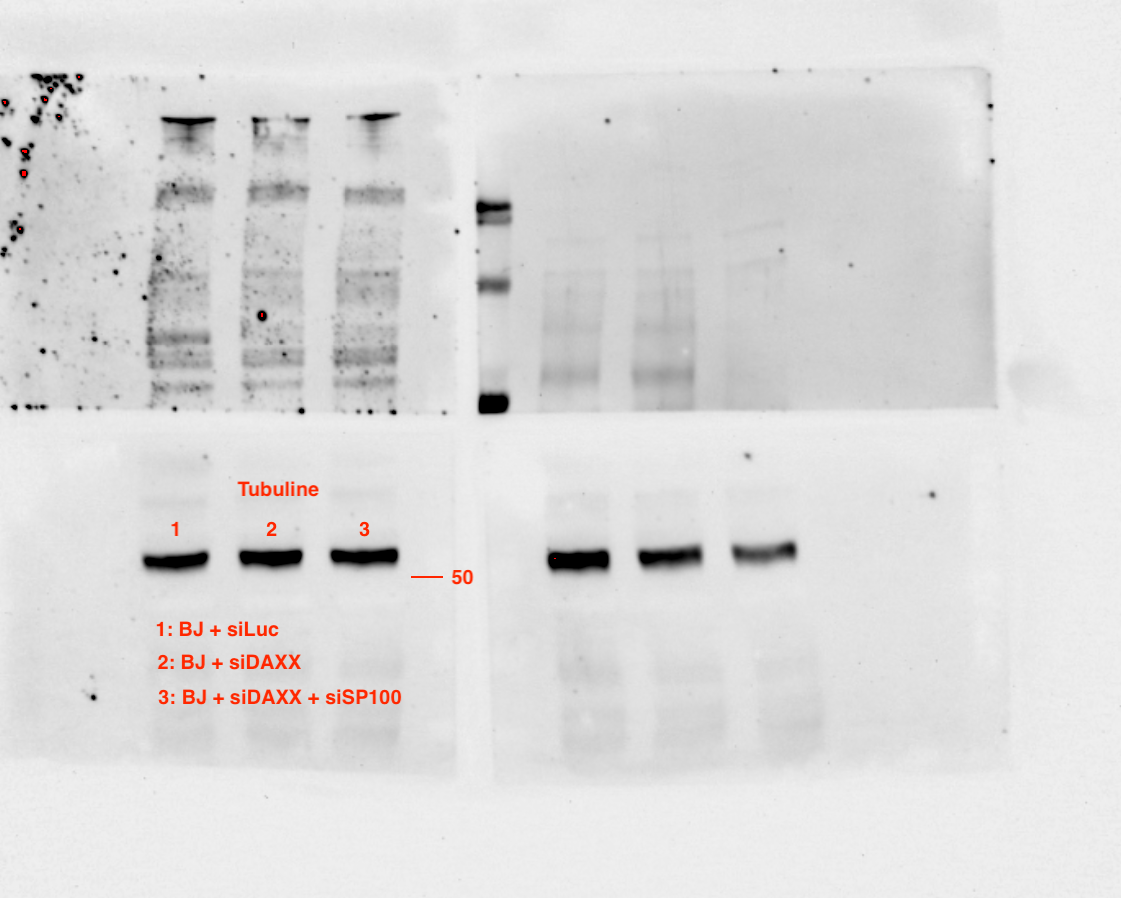

Supplement: Figure 4—figure supplement 1—source data 12. [file elife-80156-fig4-figsupp1-data12.tif]

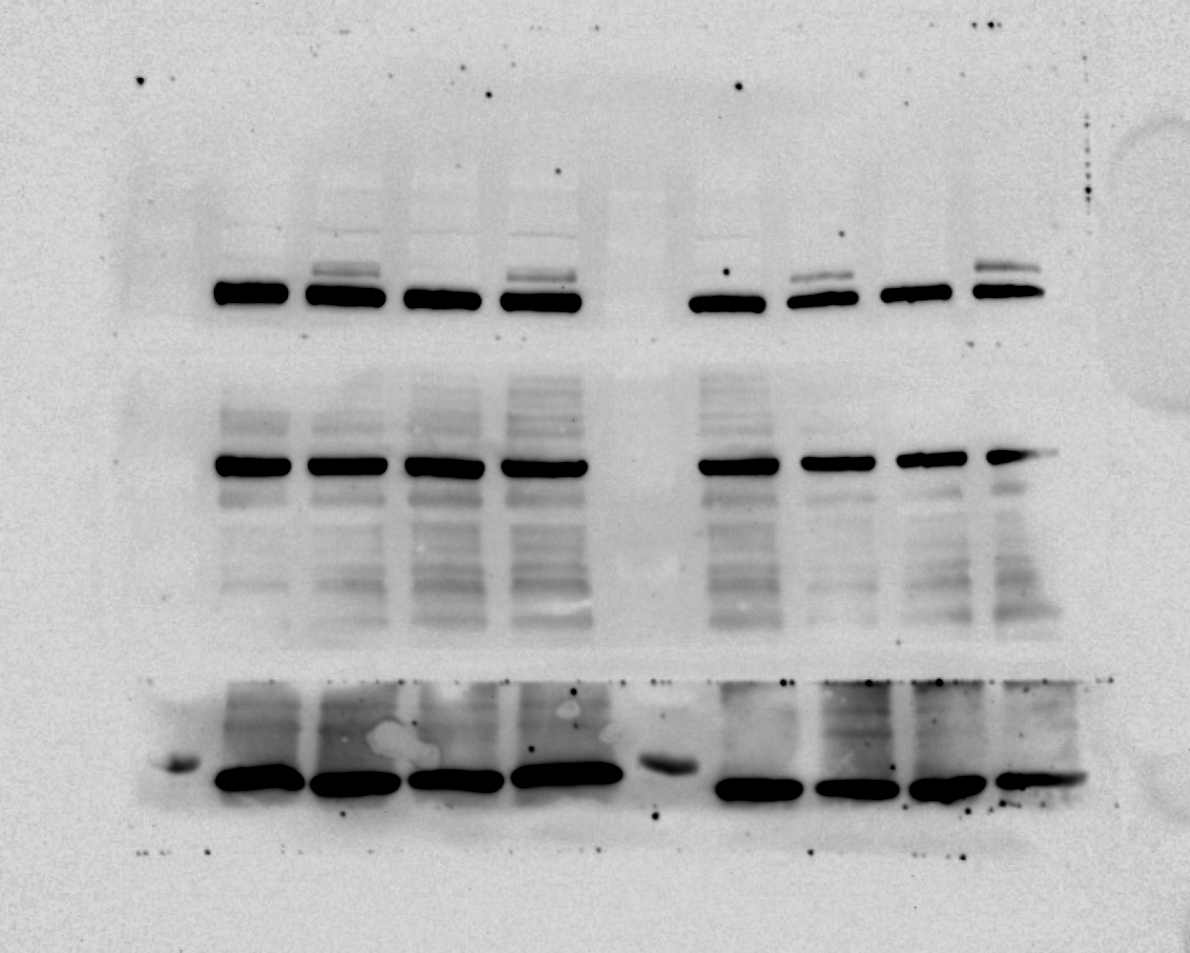

Supplement: Figure 4—figure supplement 1—source data 13. [file elife-80156-fig4-figsupp1-data13.tif]

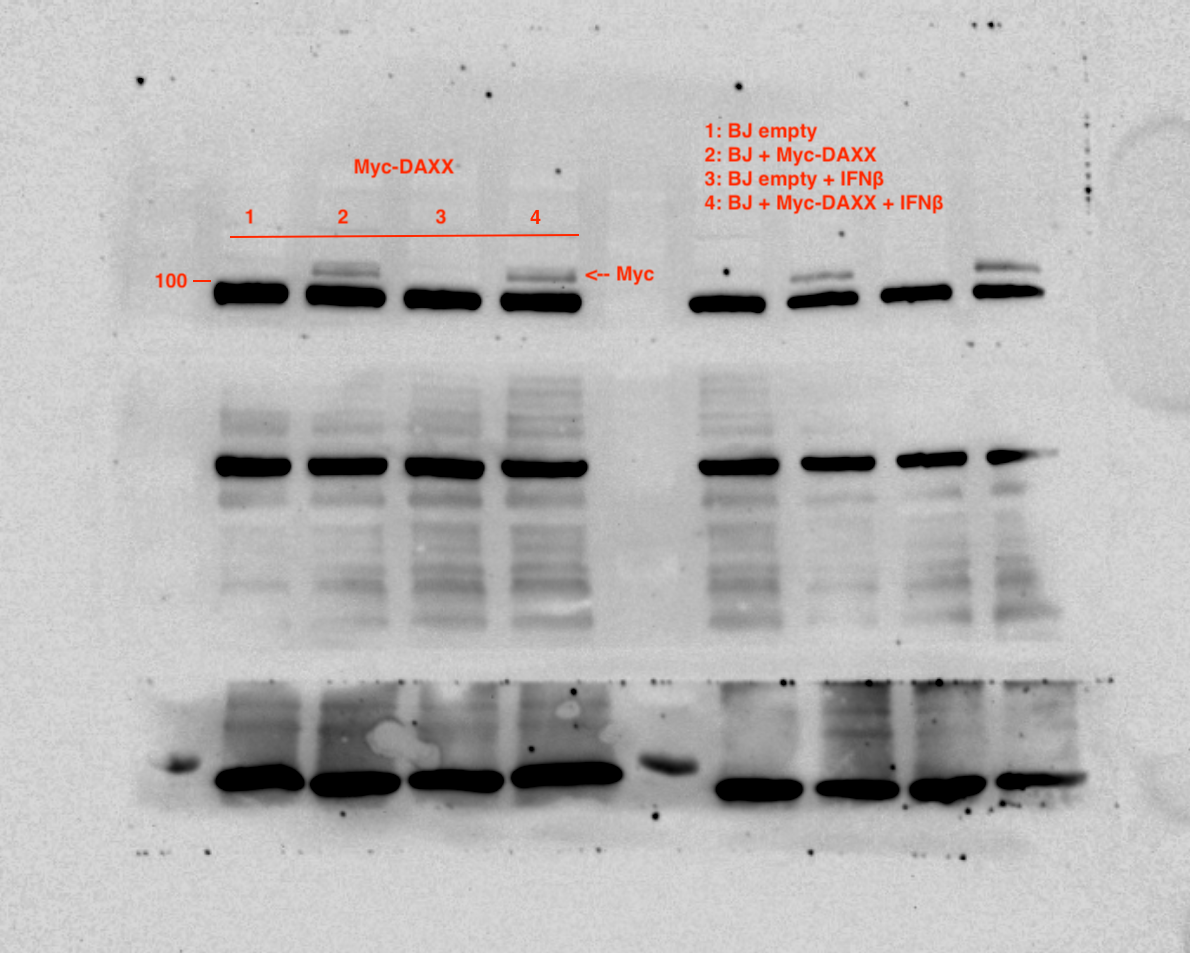

Supplement: Figure 4—figure supplement 1—source data 14. [file elife-80156-fig4-figsupp1-data14.tif]

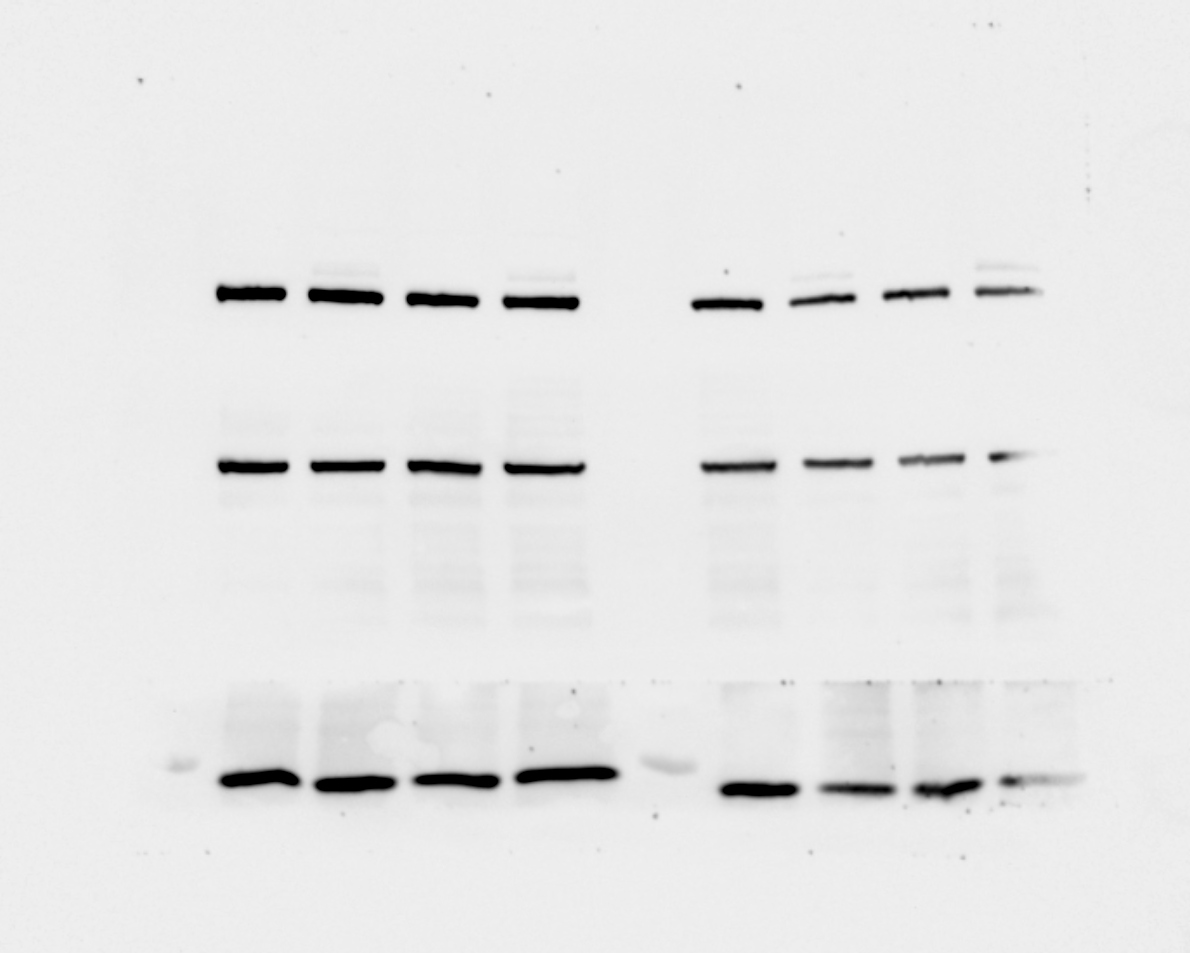

Supplement: Figure 4—figure supplement 1—source data 15. [file elife-80156-fig4-figsupp1-data15.tif]

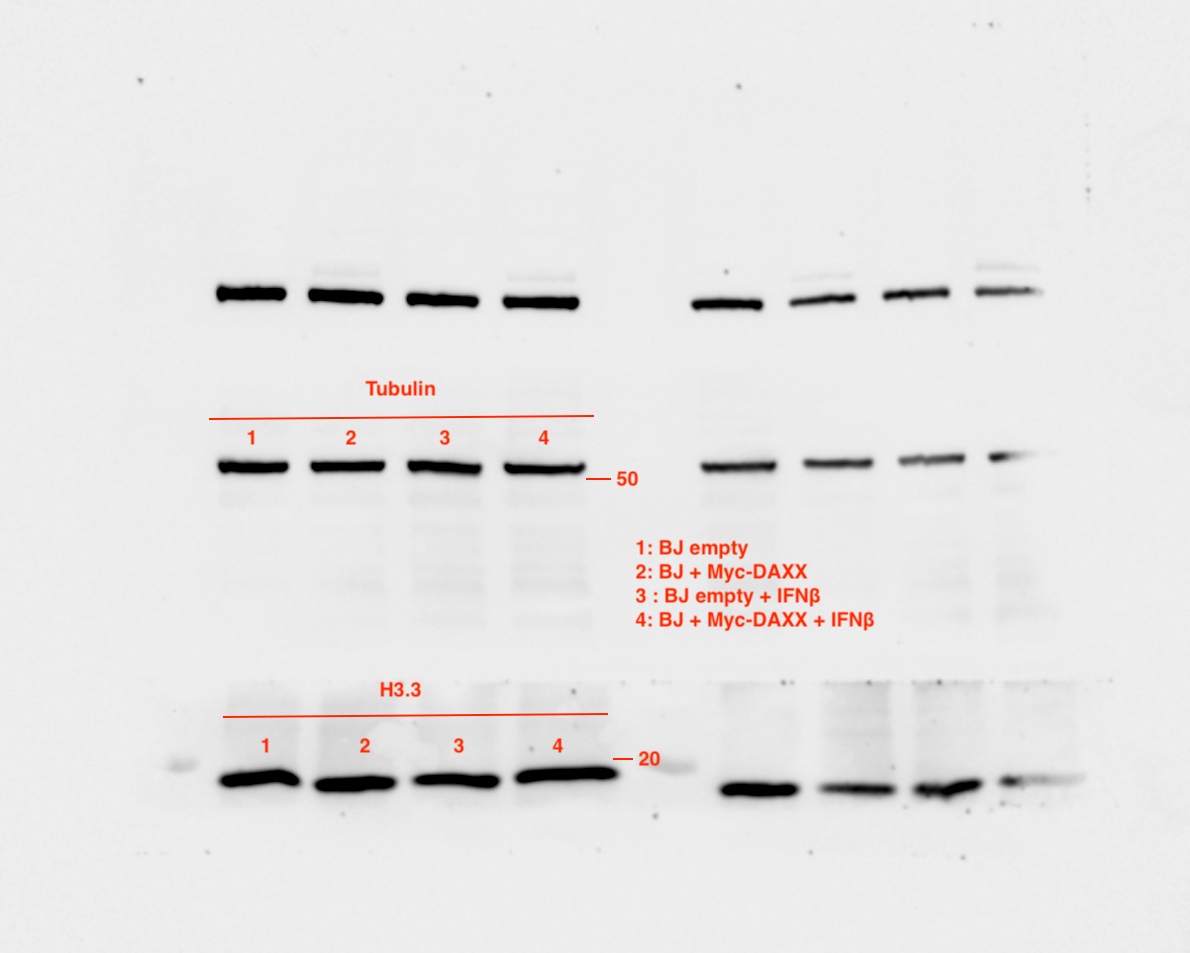

Supplement: Figure 4—figure supplement 1—source data 16. [file elife-80156-fig4-figsupp1-data16.tif]

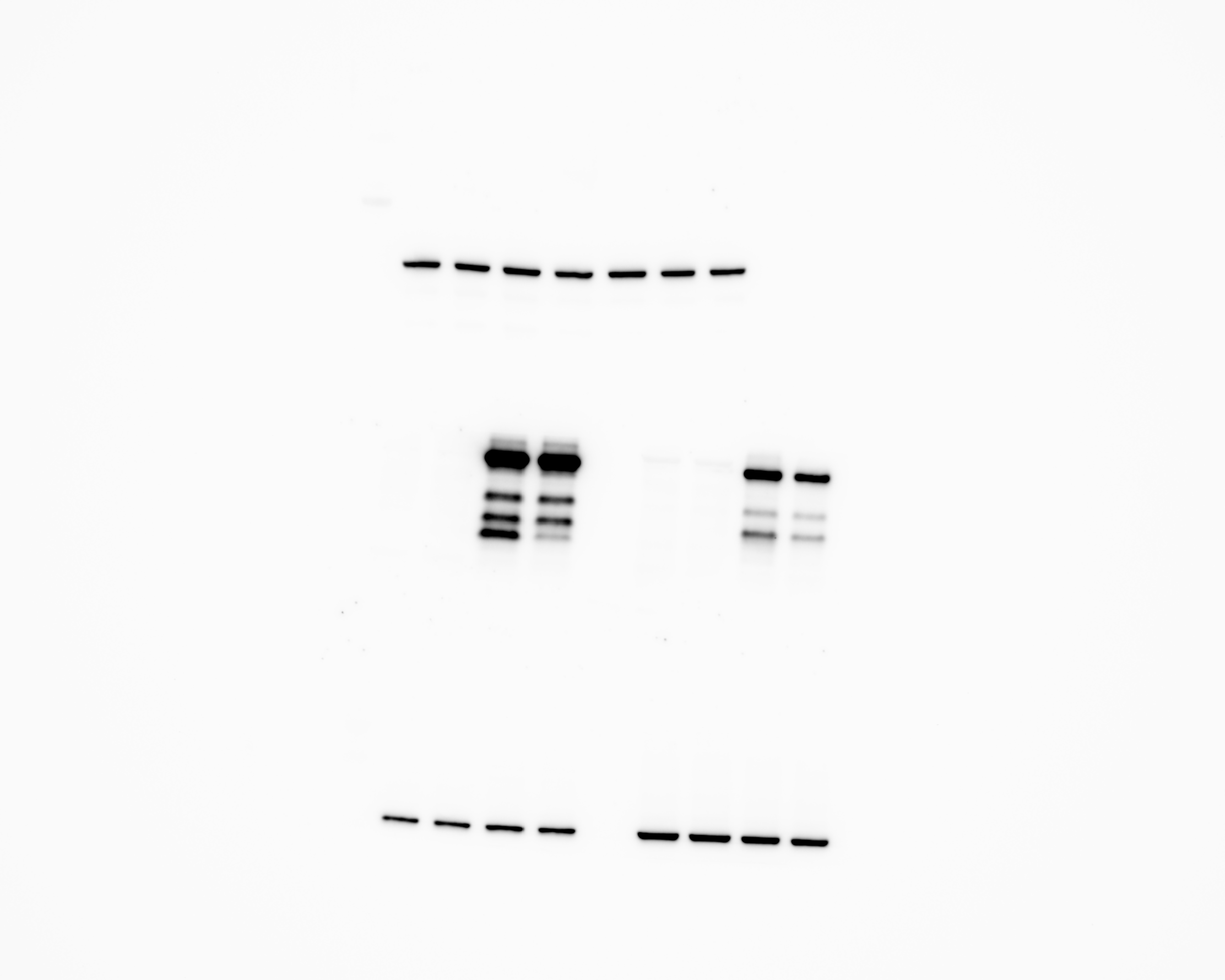

Supplement: Figure 4—figure supplement 2—source data 1. [file elife-80156-fig4-figsupp2-data1.tif]

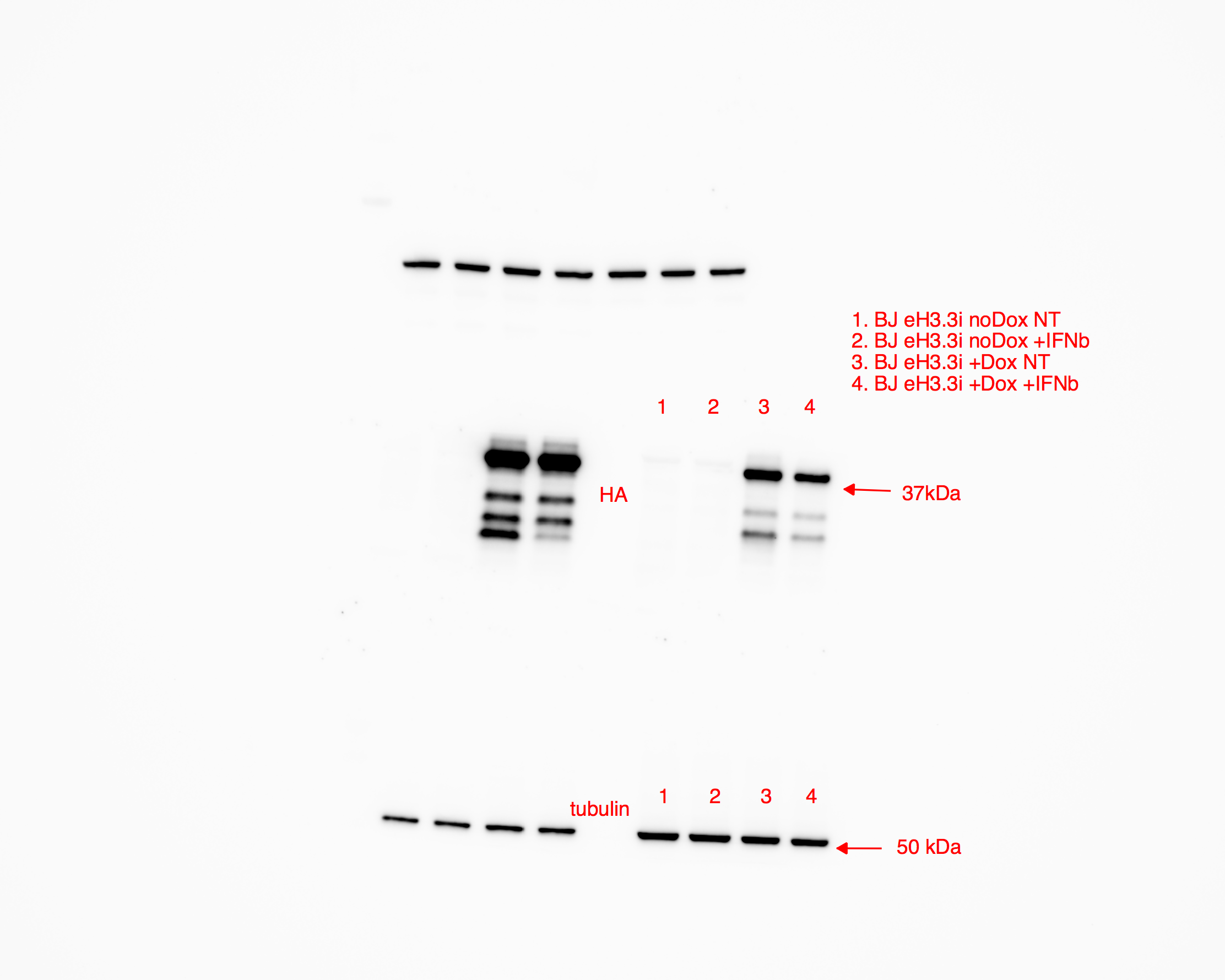

Supplement: Figure 4—figure supplement 2—source data 2. [file elife-80156-fig4-figsupp2-data2.tif]

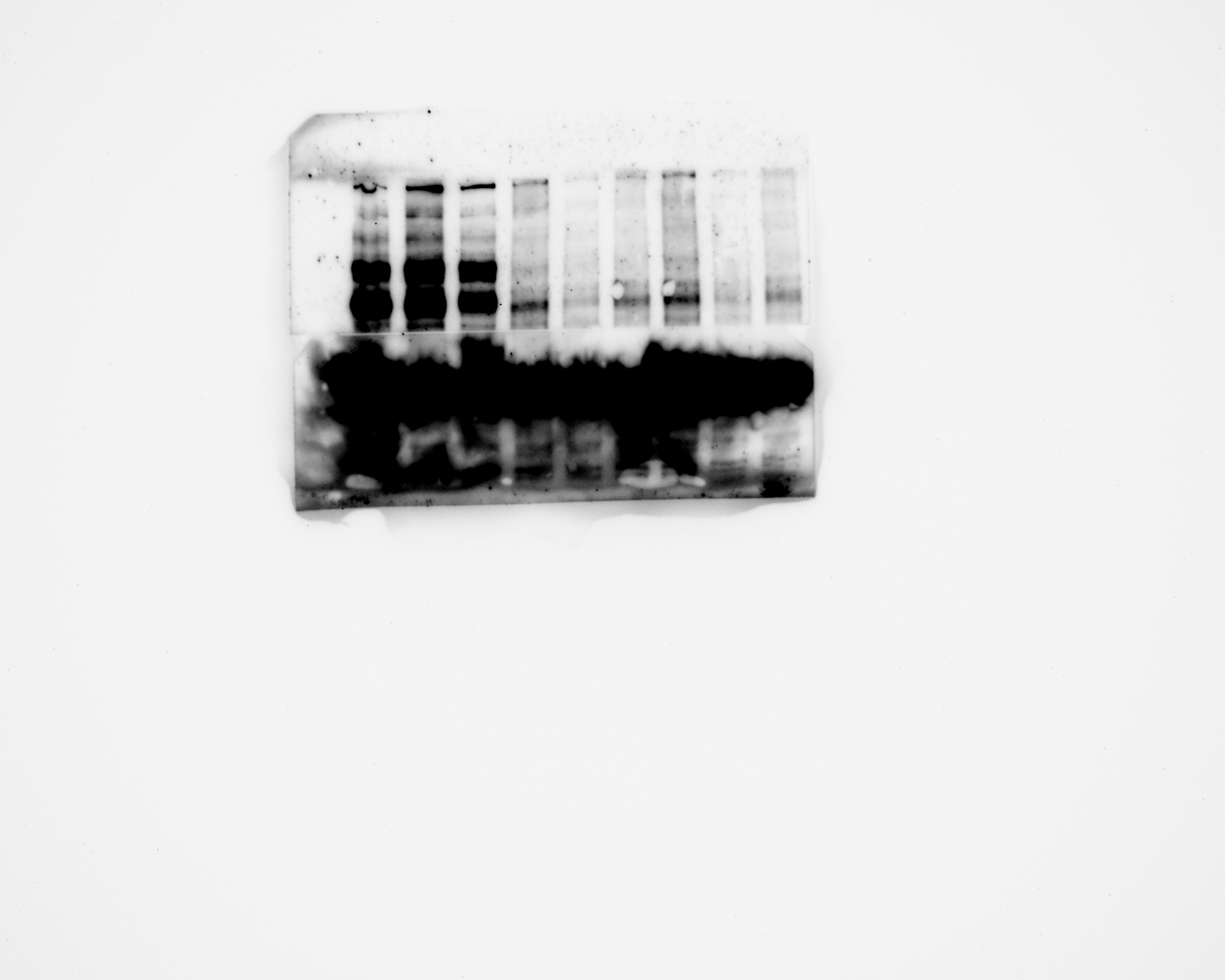

Supplement: Figure 5—figure supplement 1—source data 1. [file elife-80156-fig5-figsupp1-data1.tif]

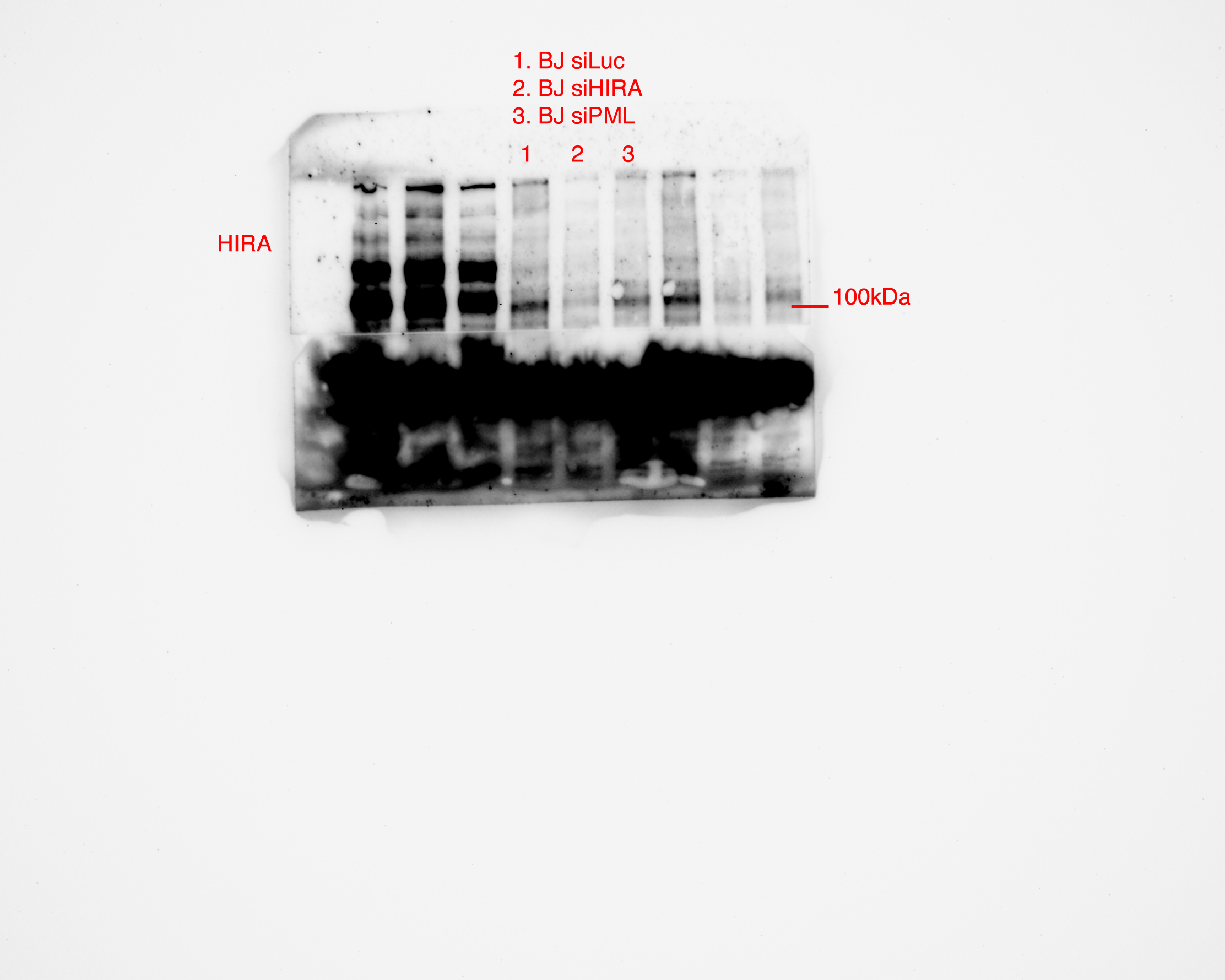

Supplement: Figure 5—figure supplement 1—source data 2. [file elife-80156-fig5-figsupp1-data2.tif]

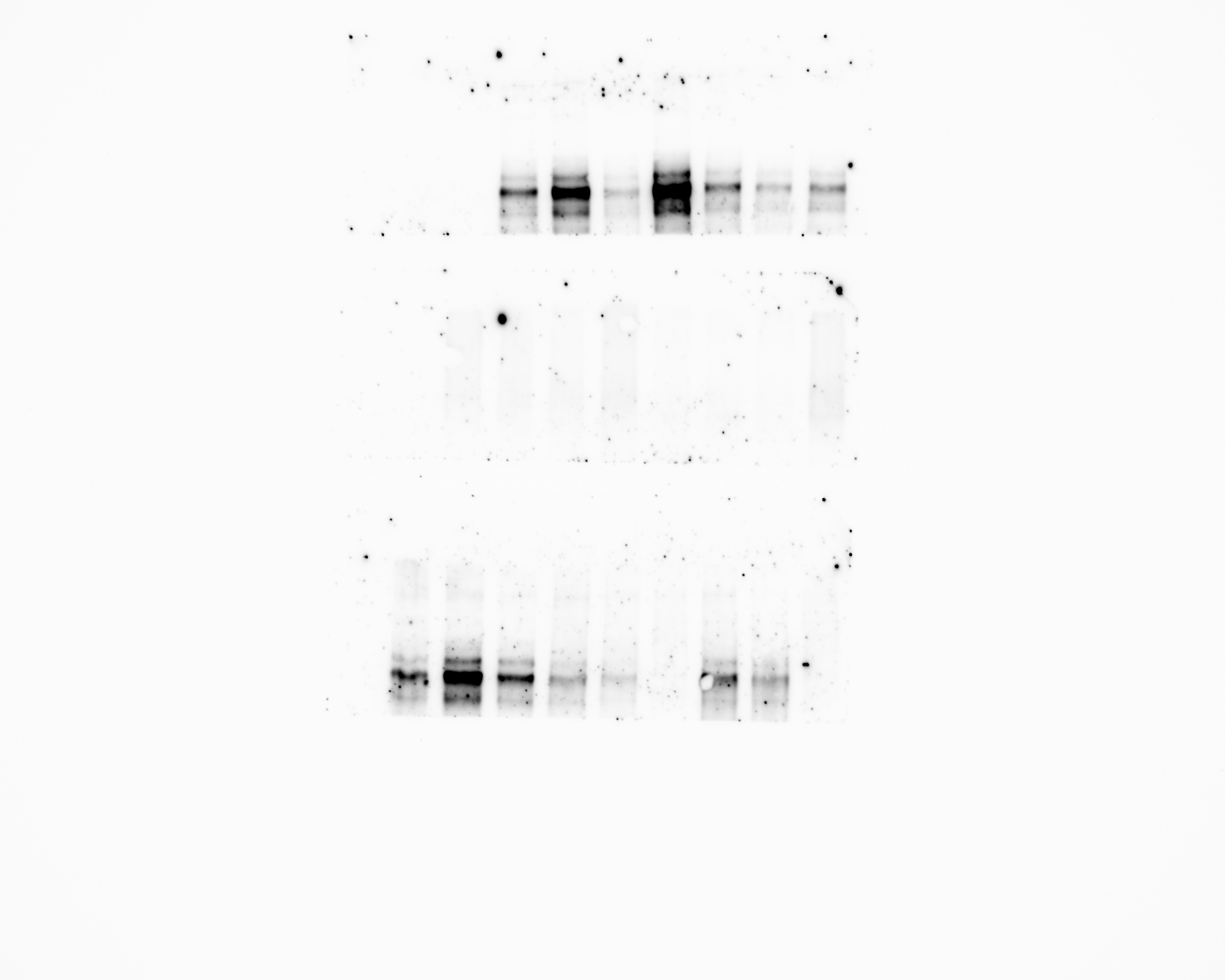

Supplement: Figure 5—figure supplement 1—source data 3. [file elife-80156-fig5-figsupp1-data3.tif]

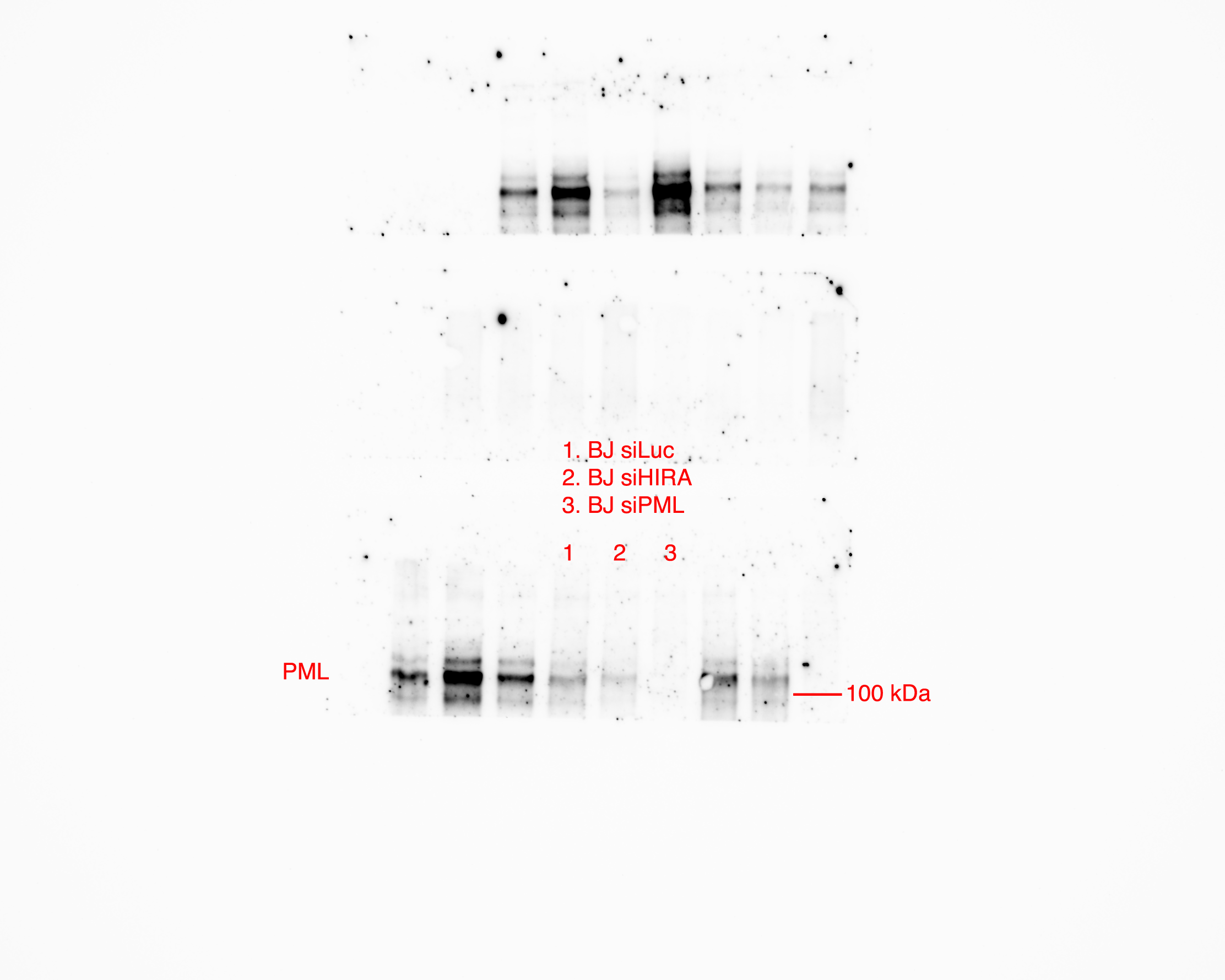

Supplement: Figure 5—figure supplement 1—source data 4. [file elife-80156-fig5-figsupp1-data4.tif]

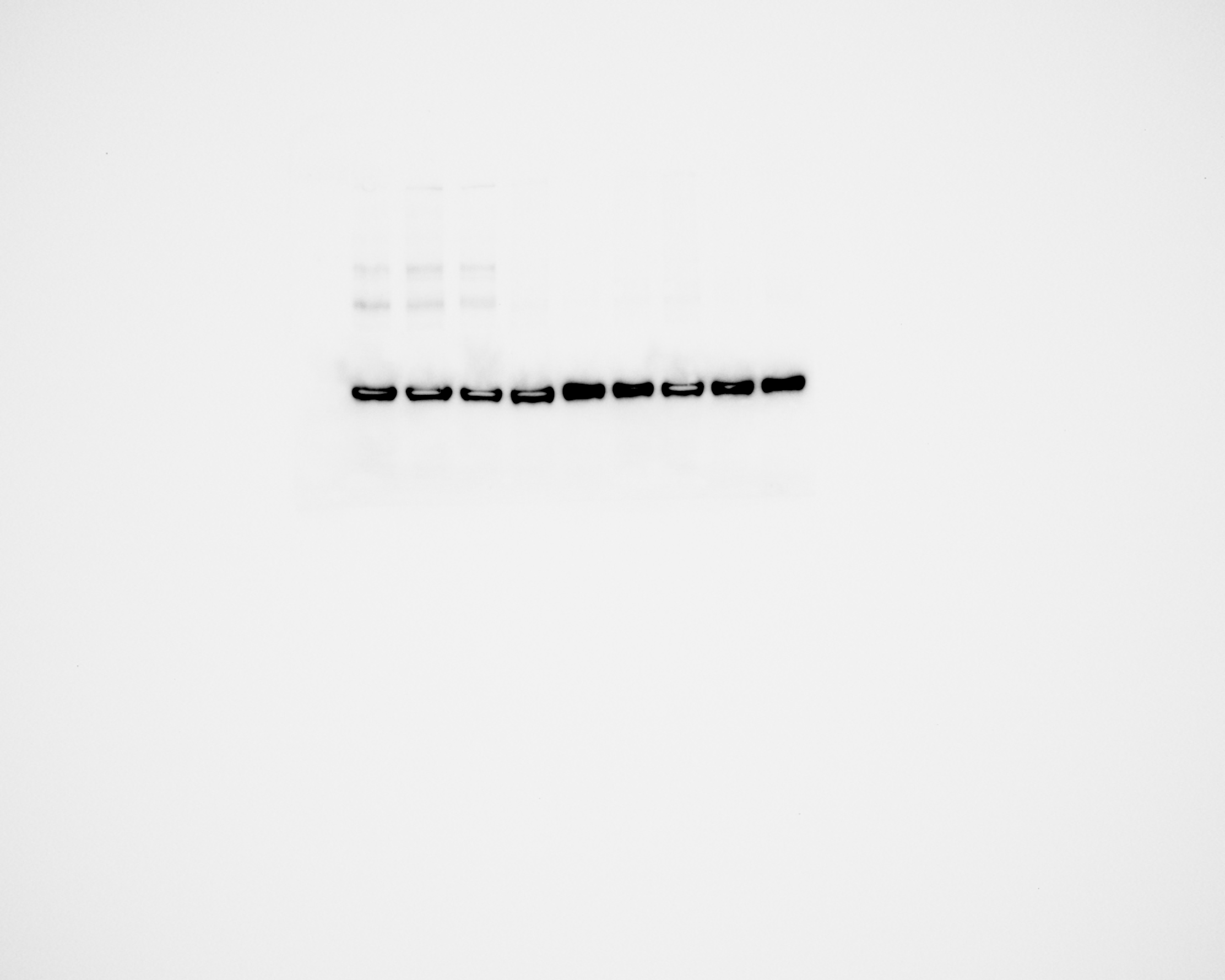

Supplement: Figure 5—figure supplement 1—source data 5. [file elife-80156-fig5-figsupp1-data5.tif]

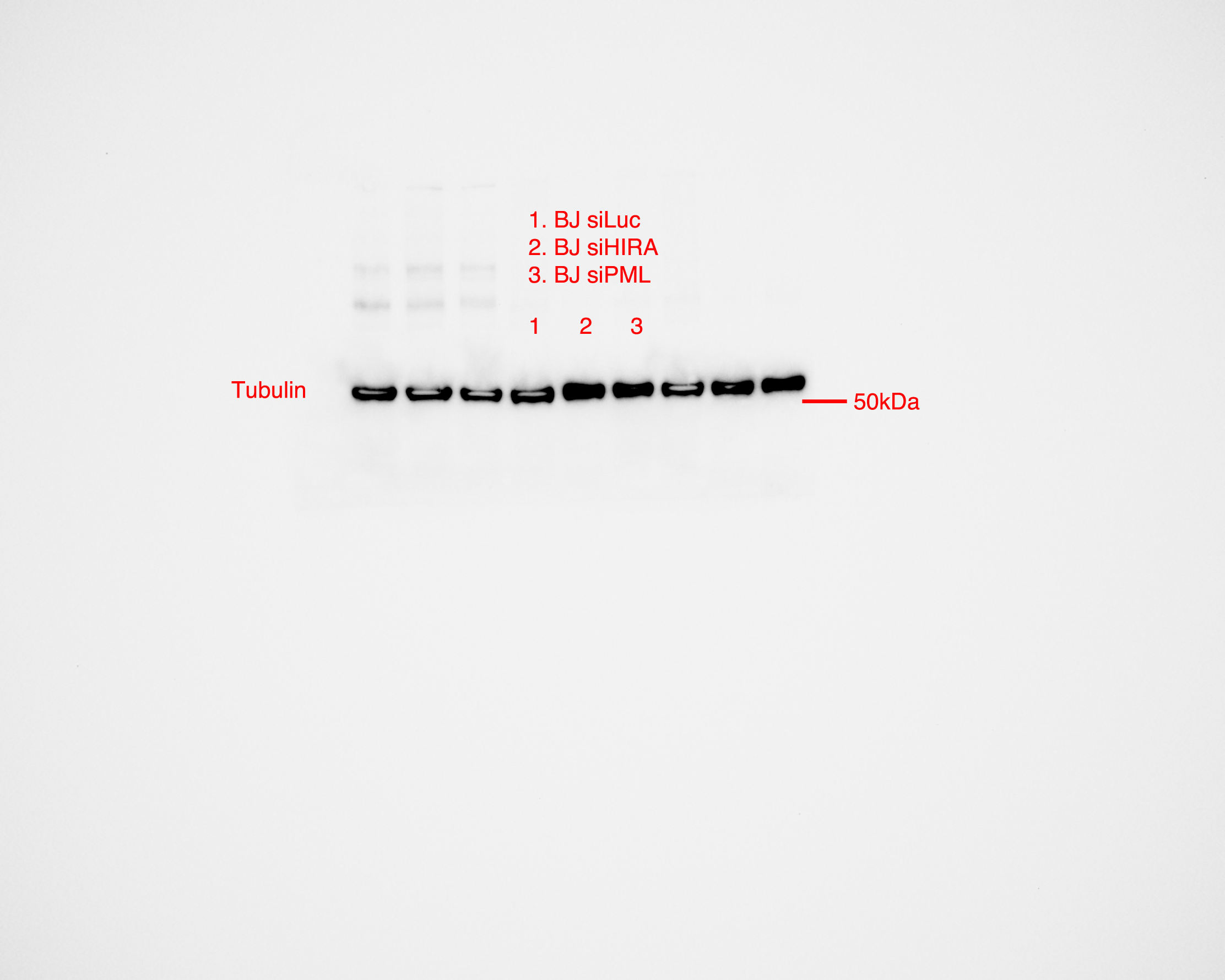

Supplement: Figure 5—figure supplement 1—source data 6. [file elife-80156-fig5-figsupp1-data6.tif]

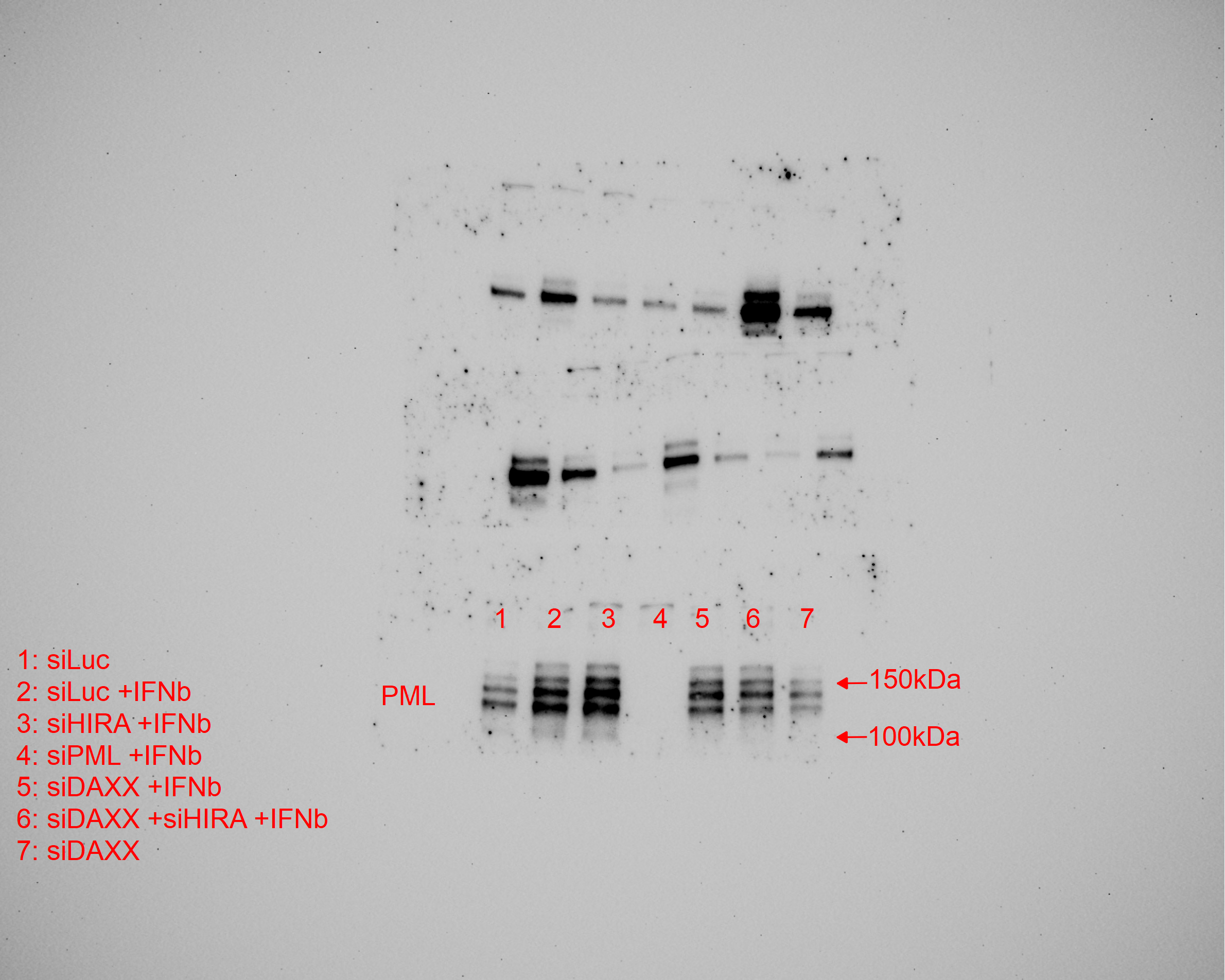

Supplement: Figure 6—source data 2. [file elife-80156-fig6-data2.tif]

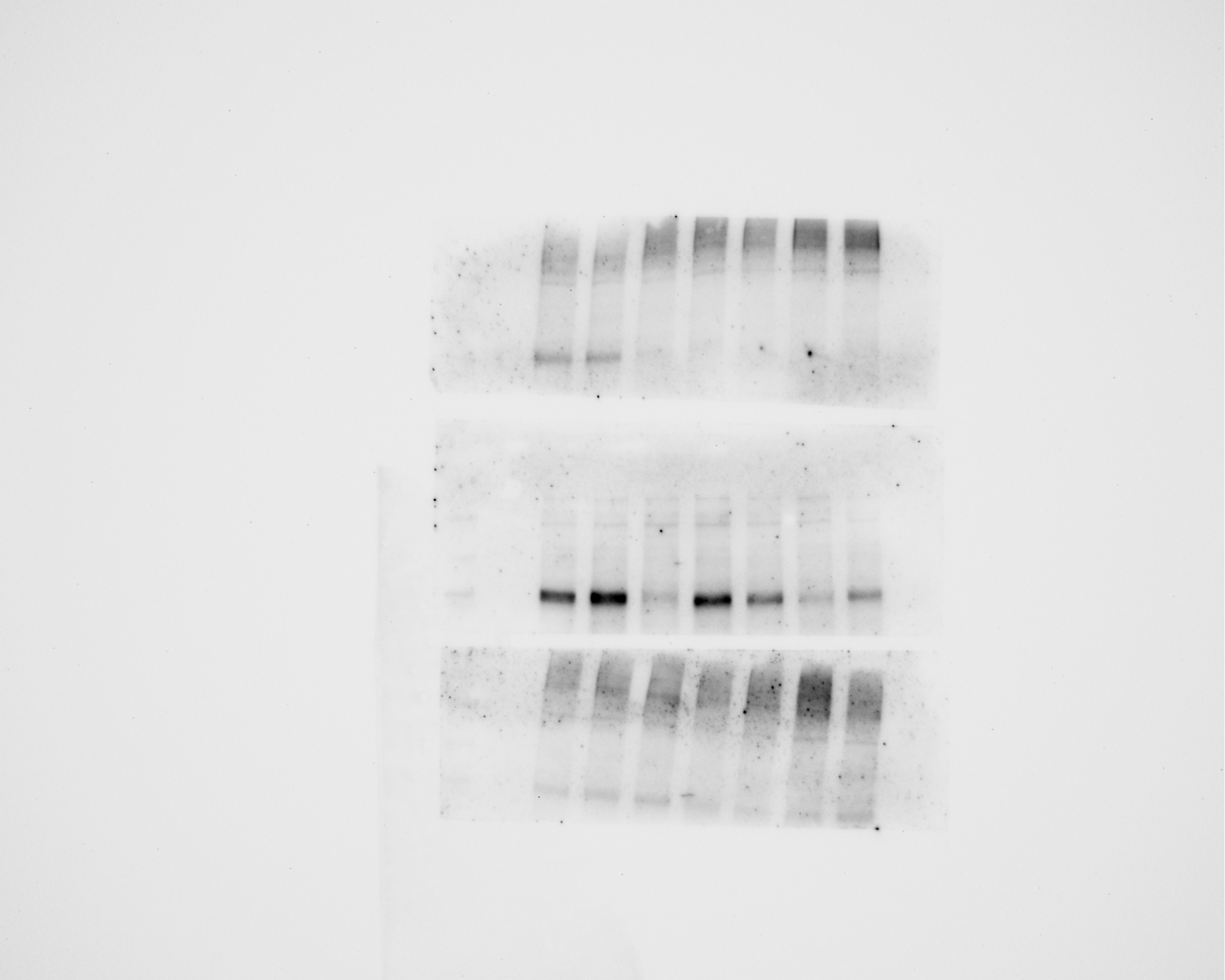

Supplement: Figure 6—source data 3. [file elife-80156-fig6-data3.tif]

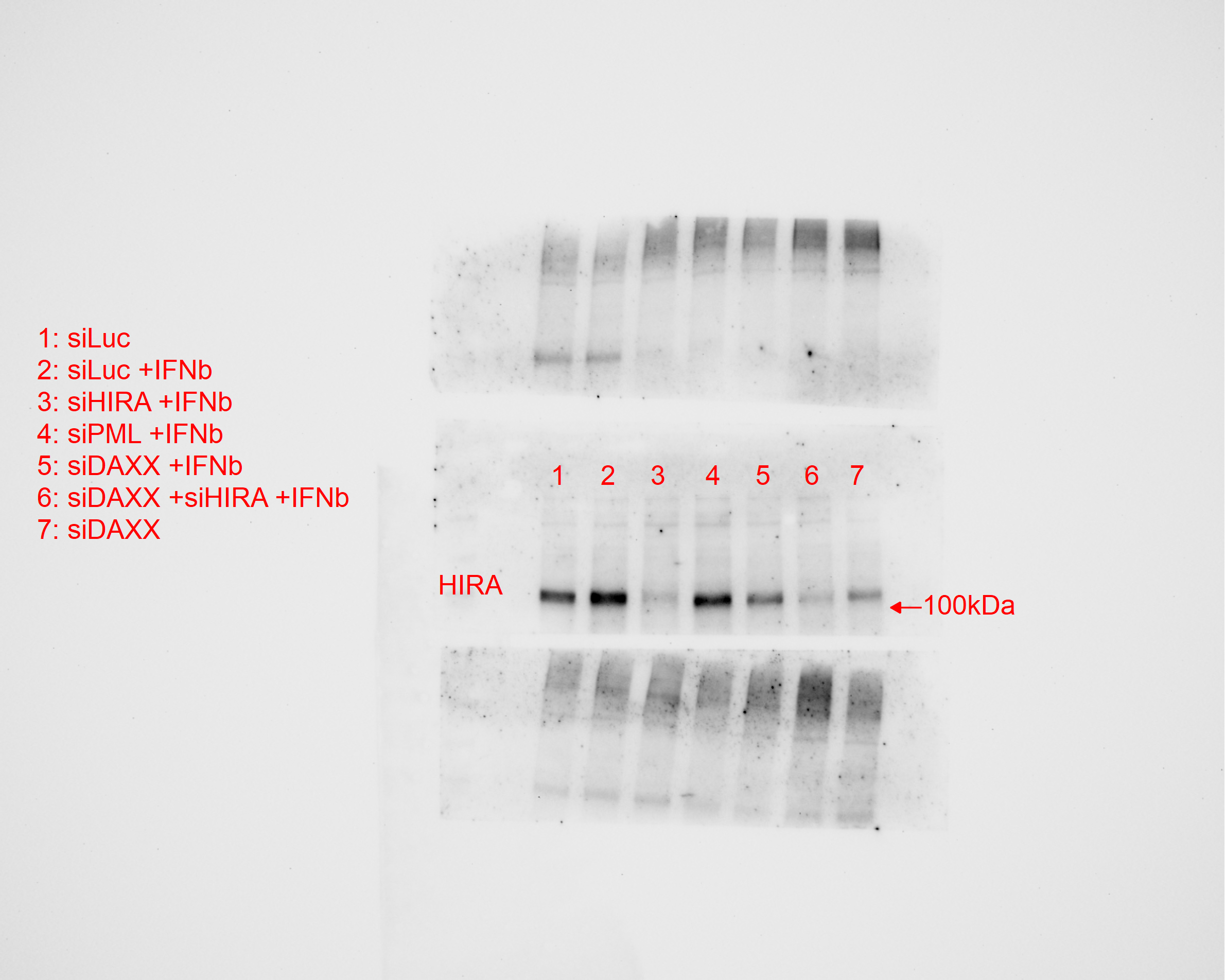

Supplement: Figure 6—source data 4. [file elife-80156-fig6-data4.tif]

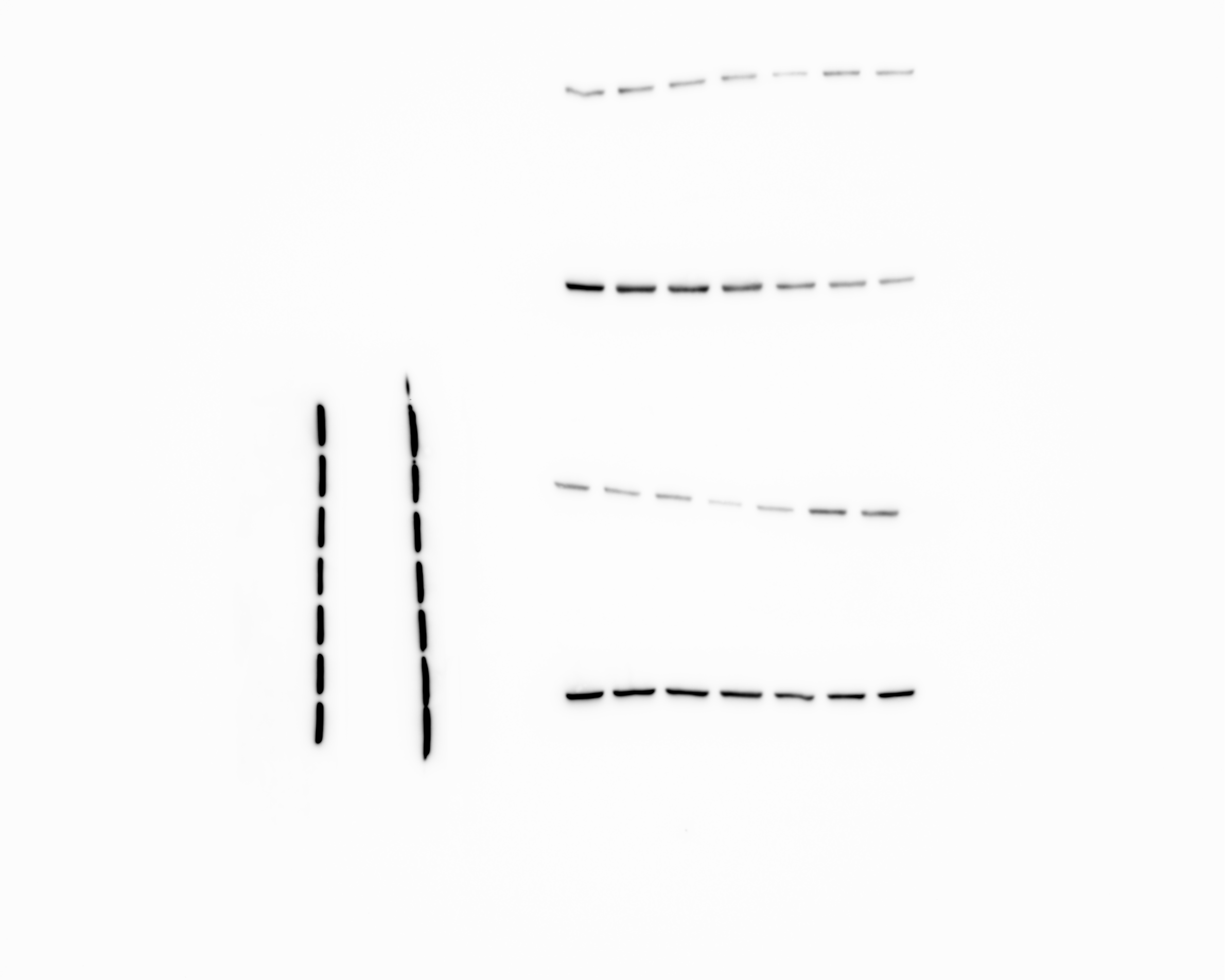

Supplement: Figure 6—source data 5. [file elife-80156-fig6-data5.tif]

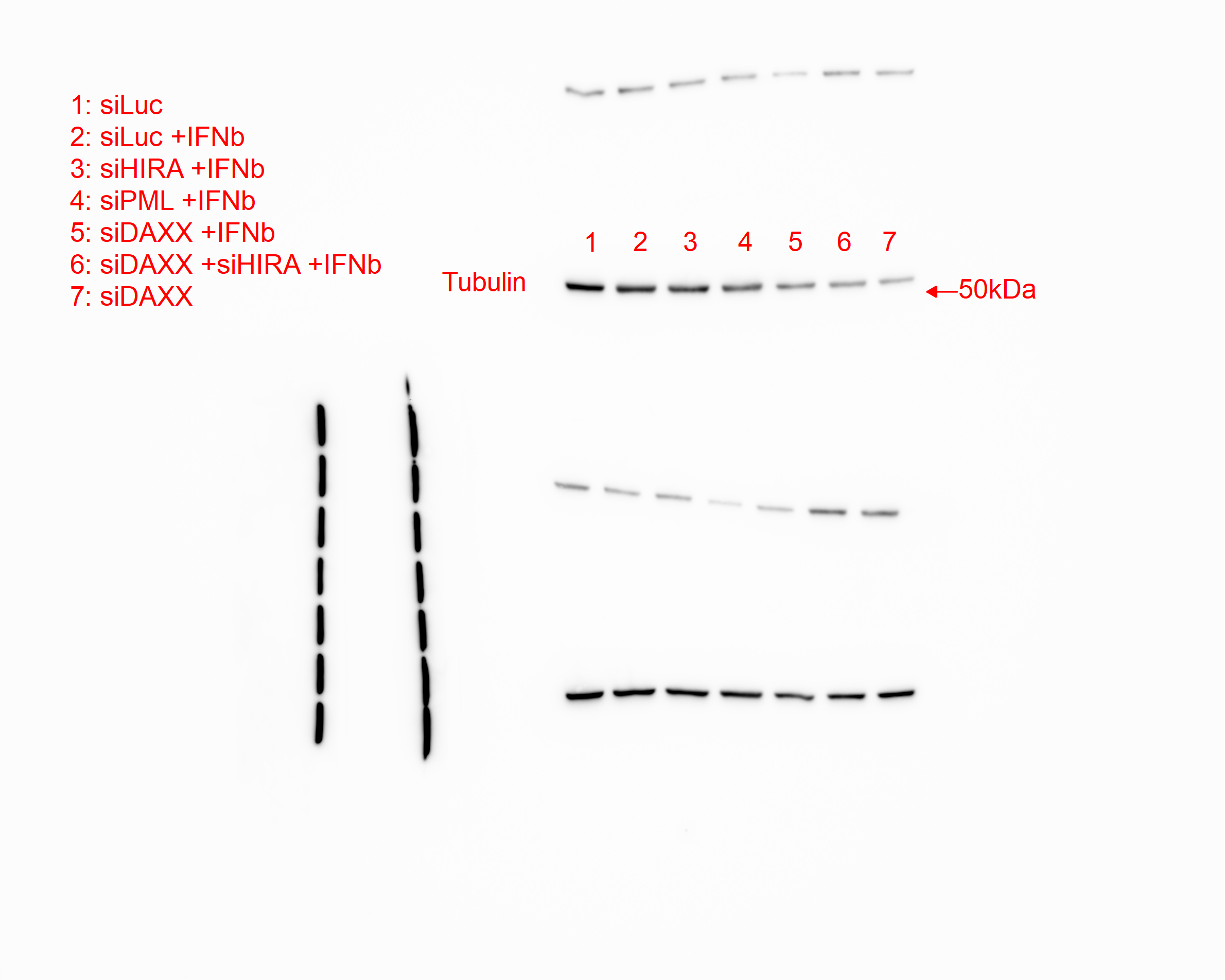

Supplement: Figure 6—source data 6. [file elife-80156-fig6-data6.tif]

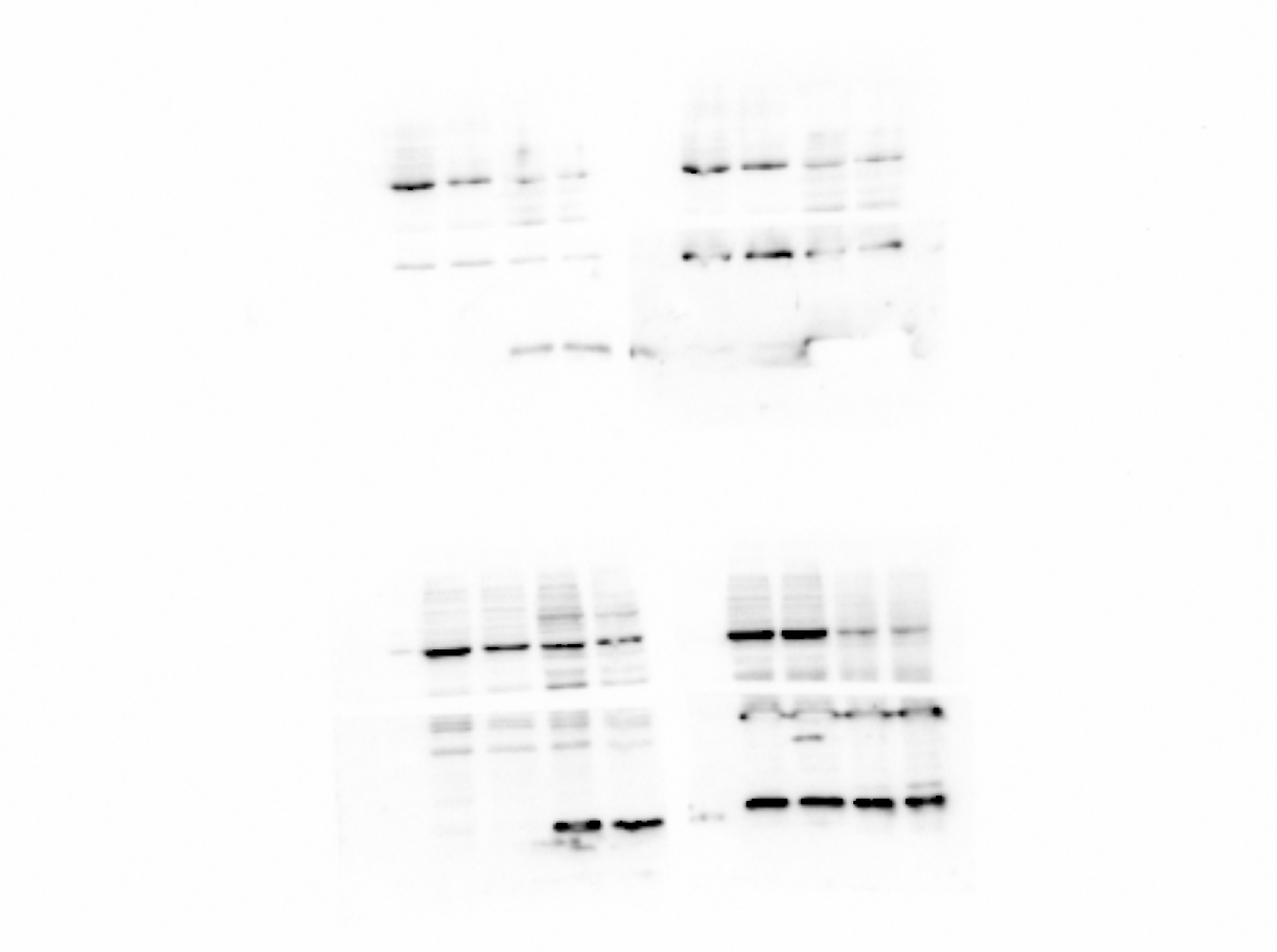

Supplement: Figure 6—figure supplement 1—source data 1. [file elife-80156-fig6-figsupp1-data1.tif]

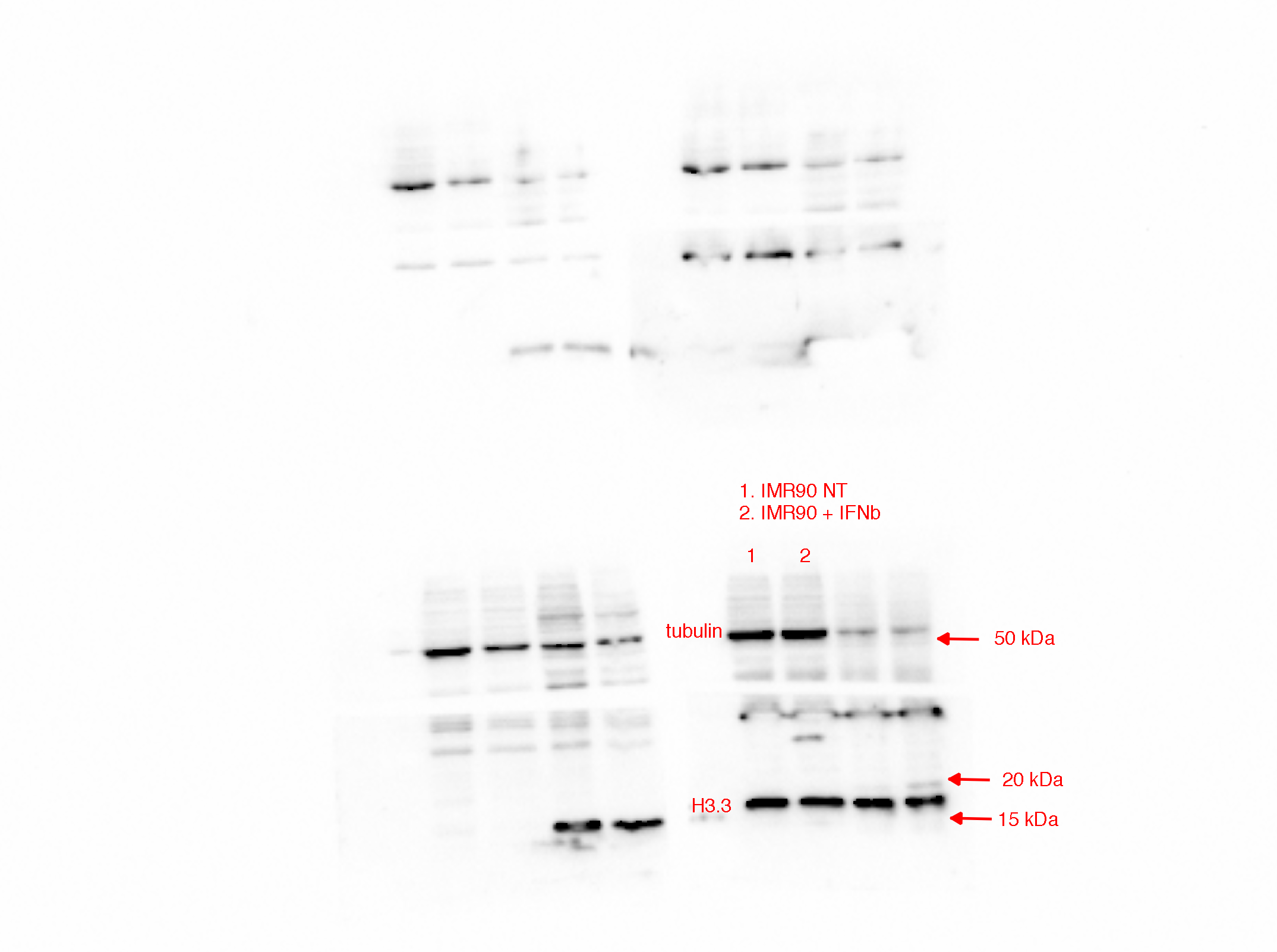

Supplement: Figure 6—figure supplement 1—source data 2. [file elife-80156-fig6-figsupp1-data2.tif]

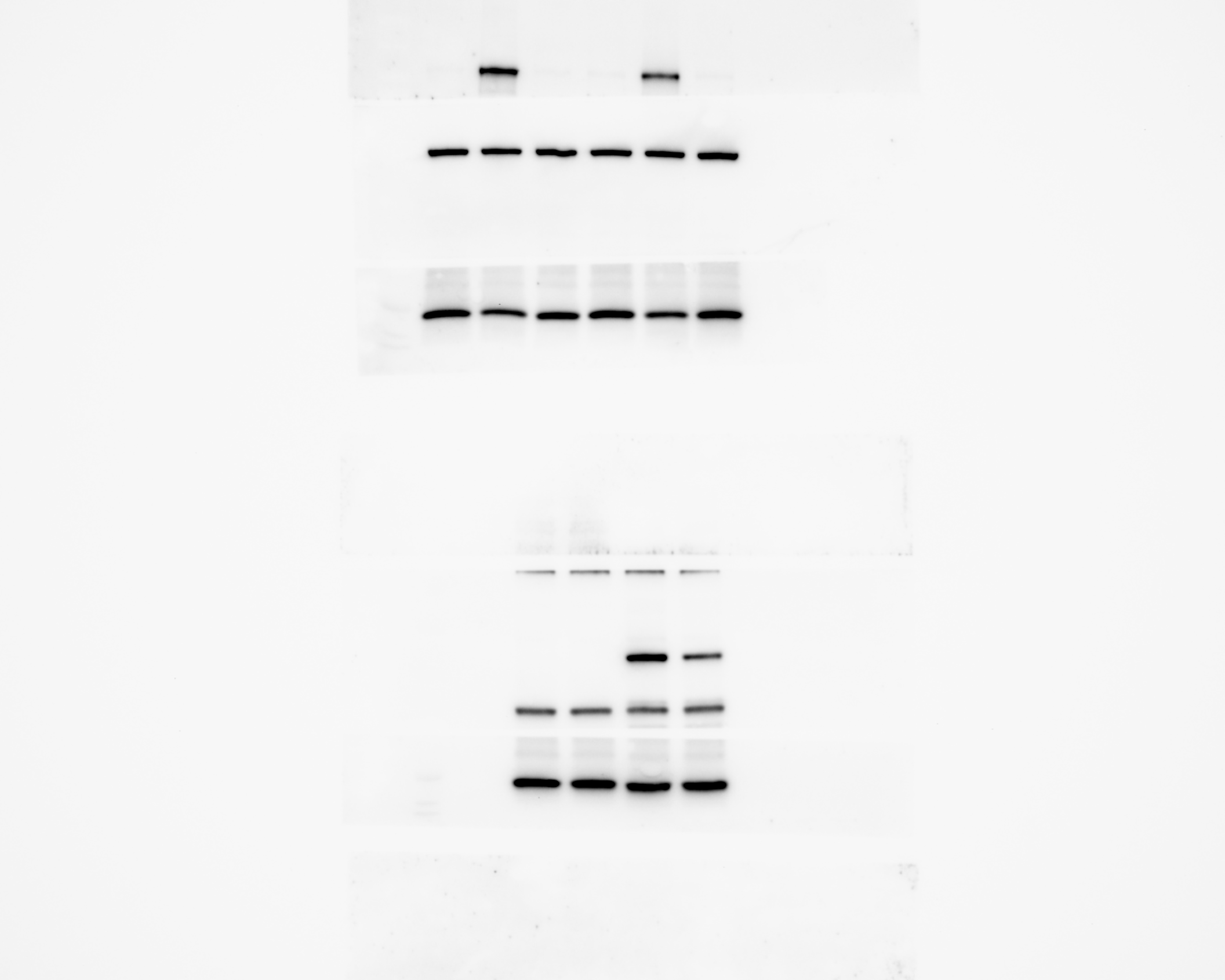

Supplement: Figure 6—figure supplement 2—source data 1. [file elife-80156-fig6-figsupp2-data1.tif]

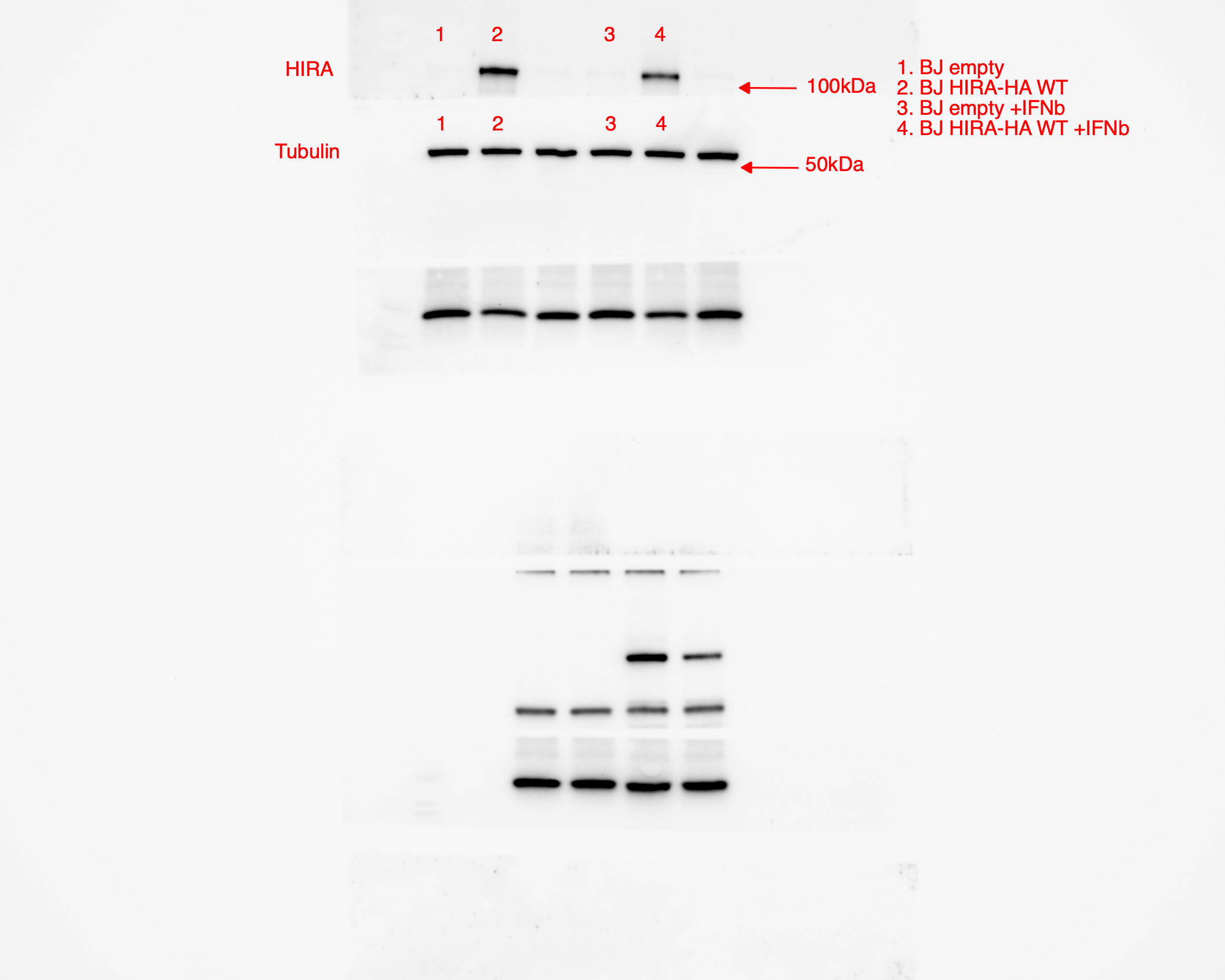

Supplement: Figure 6—figure supplement 2—source data 2. [file elife-80156-fig6-figsupp2-data2.tif]

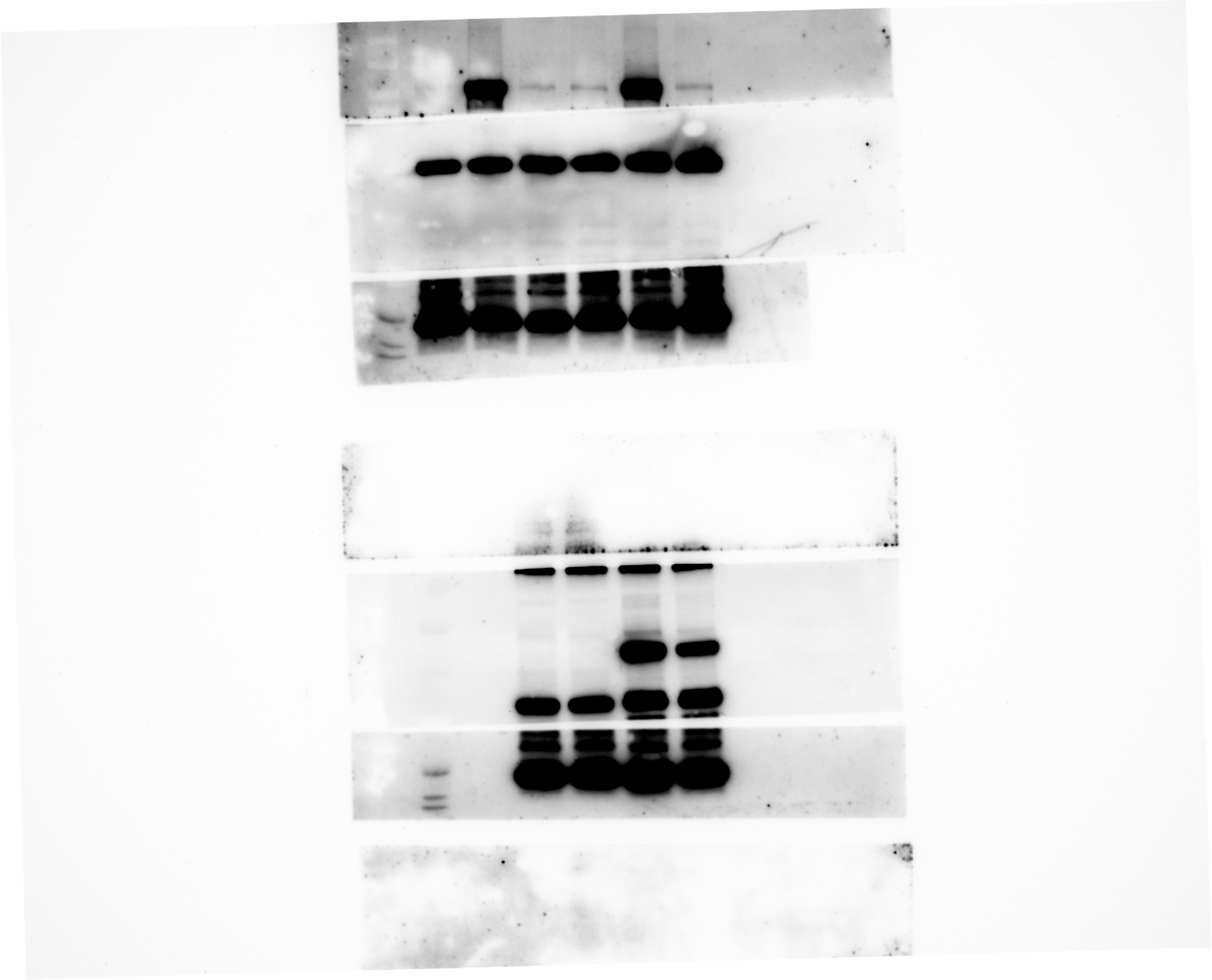

Supplement: Figure 6—figure supplement 2—source data 3. [file elife-80156-fig6-figsupp2-data3.tif]

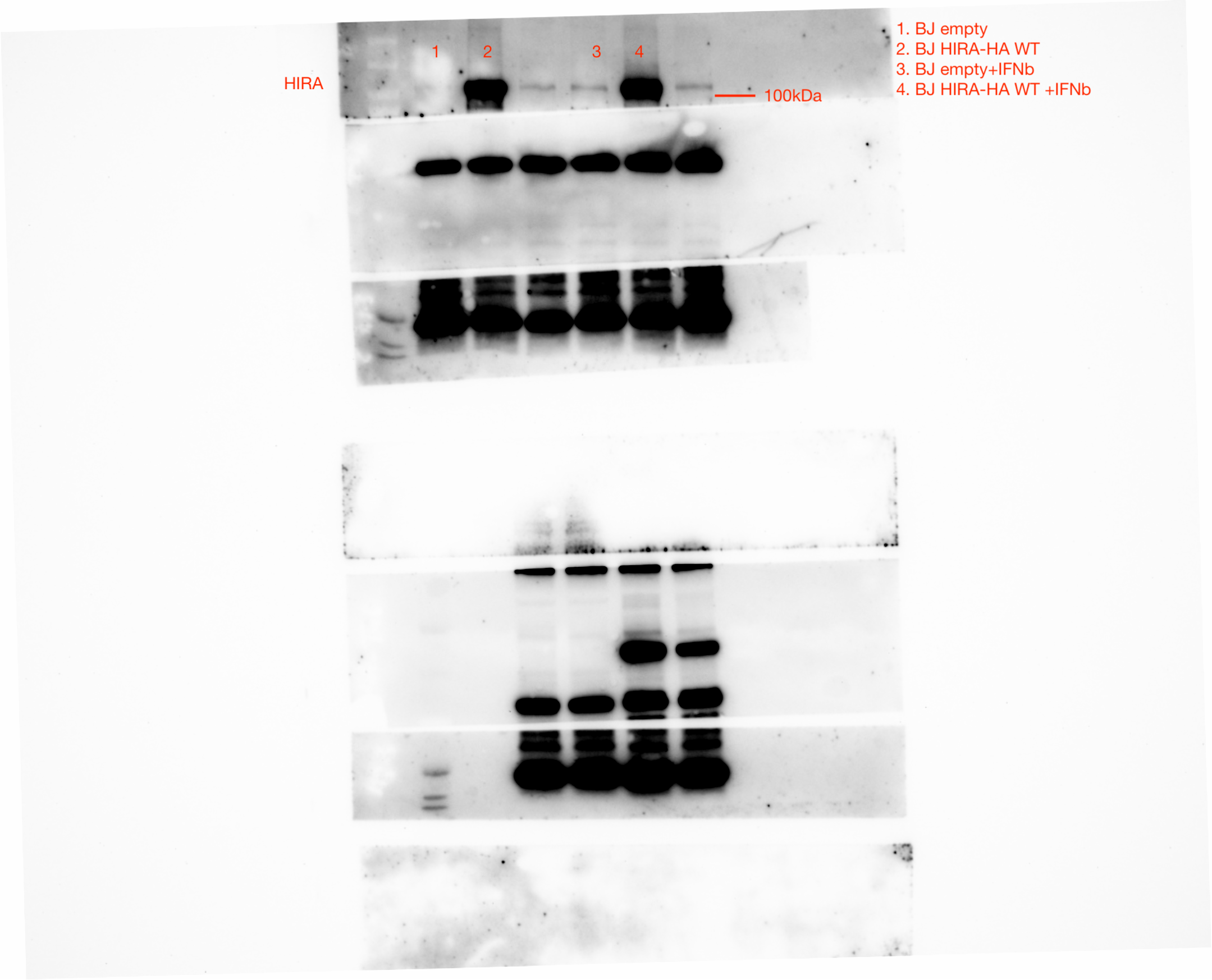

Supplement: Figure 6—figure supplement 2—source data 4. [file elife-80156-fig6-figsupp2-data4.tif]

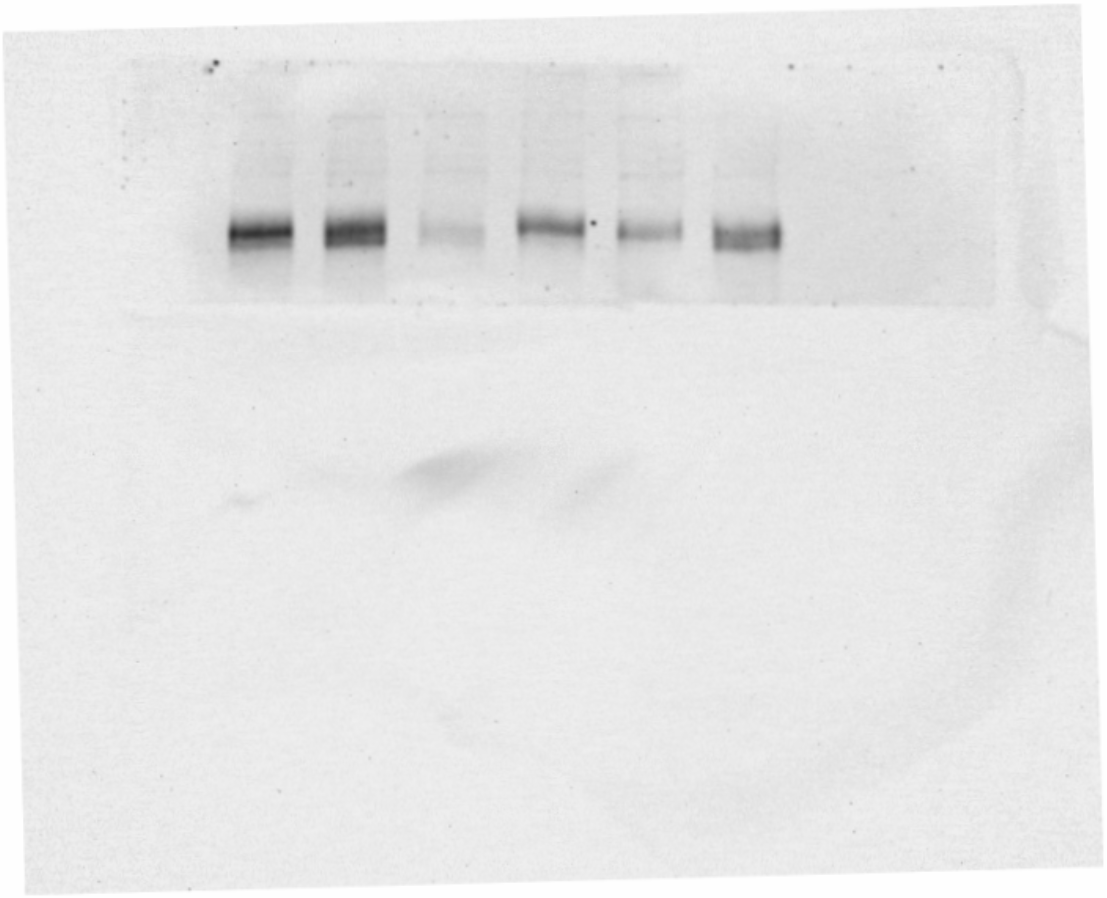

Supplement: Figure 7—source data 1. [file elife-80156-fig7-data1.tif]

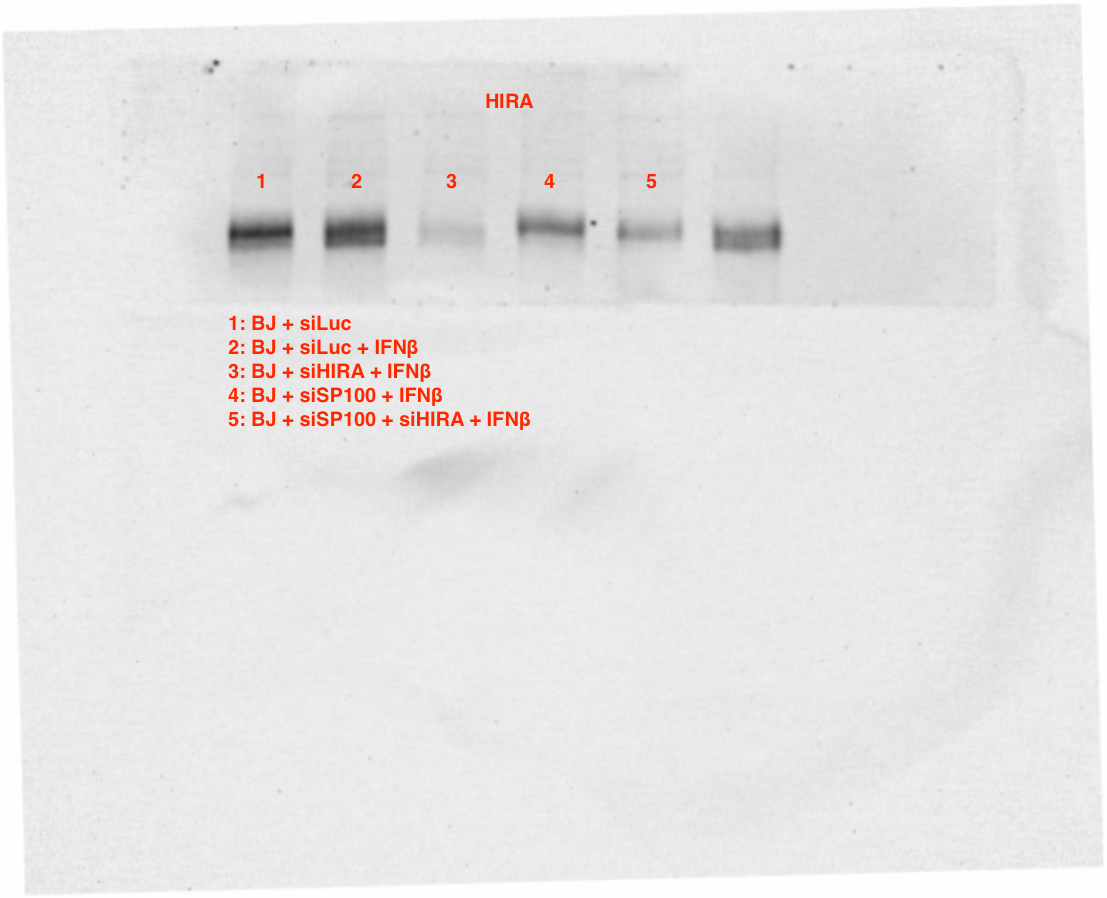

Supplement: Figure 7—source data 2. [file elife-80156-fig7-data2.tif]

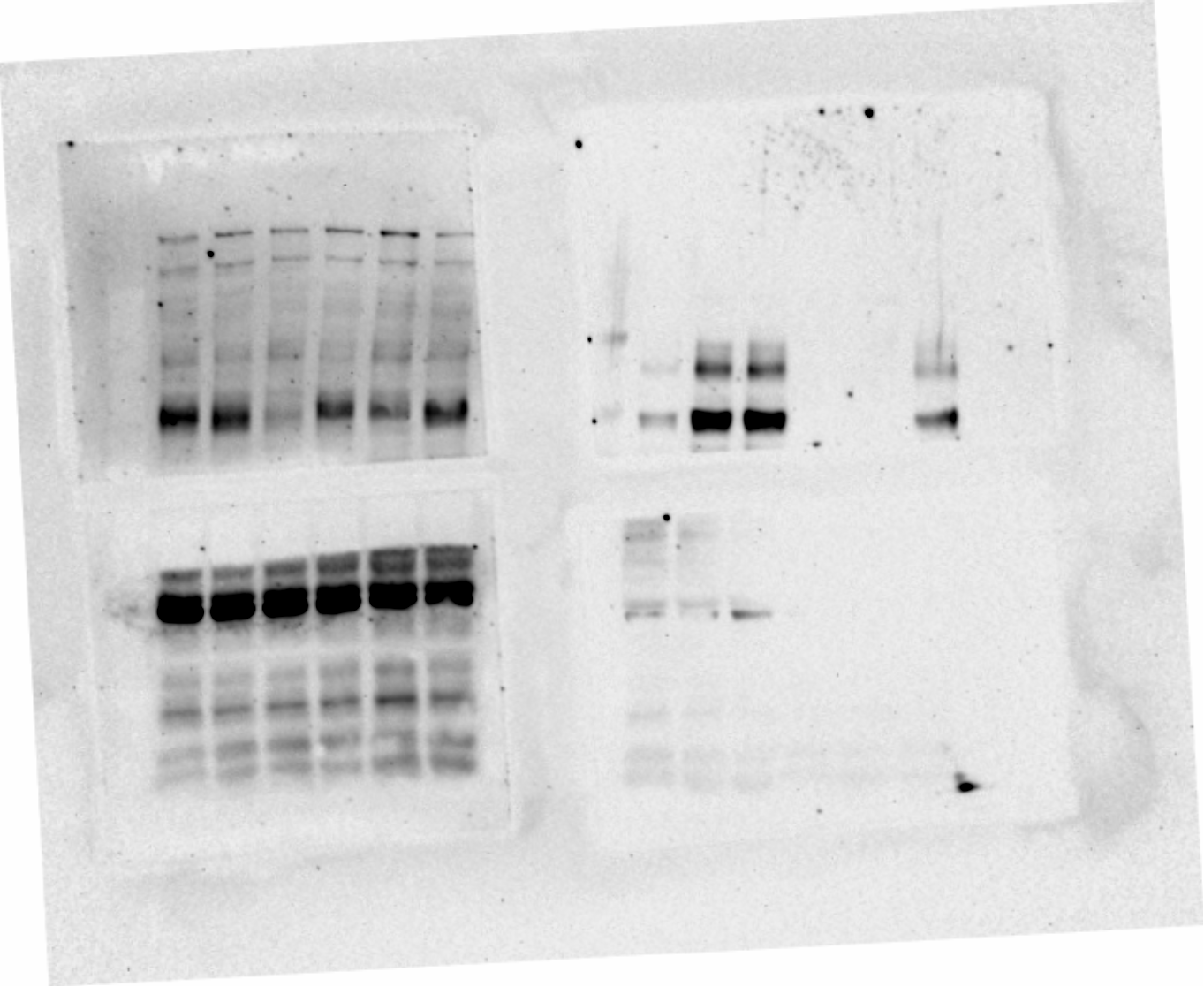

Supplement: Figure 7—source data 3. [file elife-80156-fig7-data3.tif]

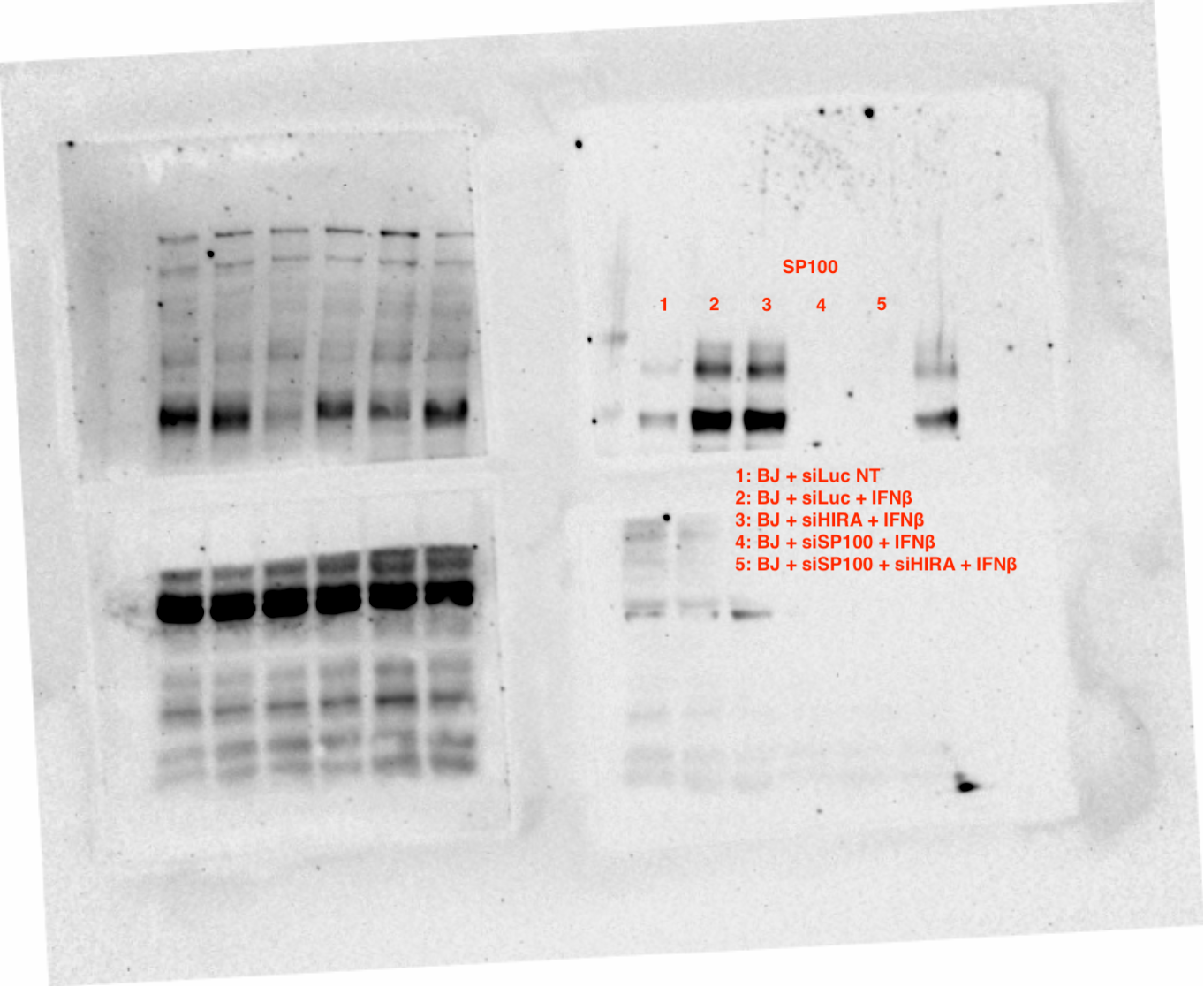

Supplement: Figure 7—source data 4. [file elife-80156-fig7-data4.tif]

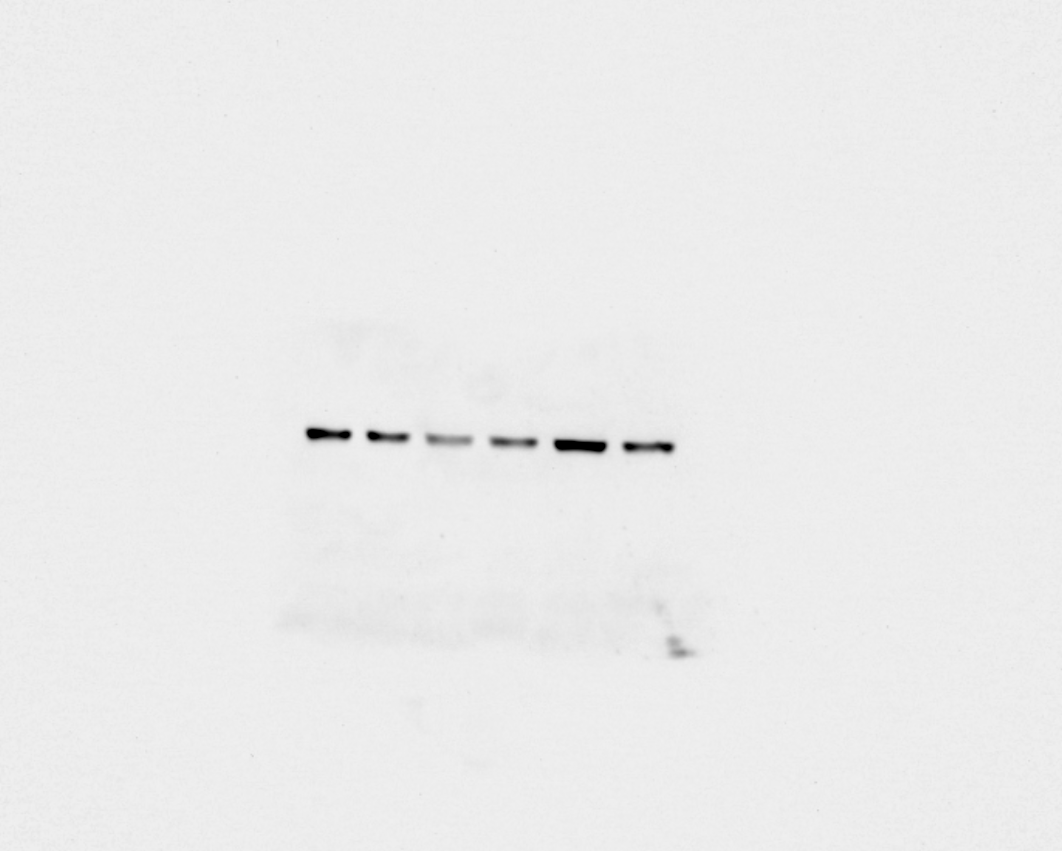

Supplement: Figure 7—source data 5. [file elife-80156-fig7-data5.tif]

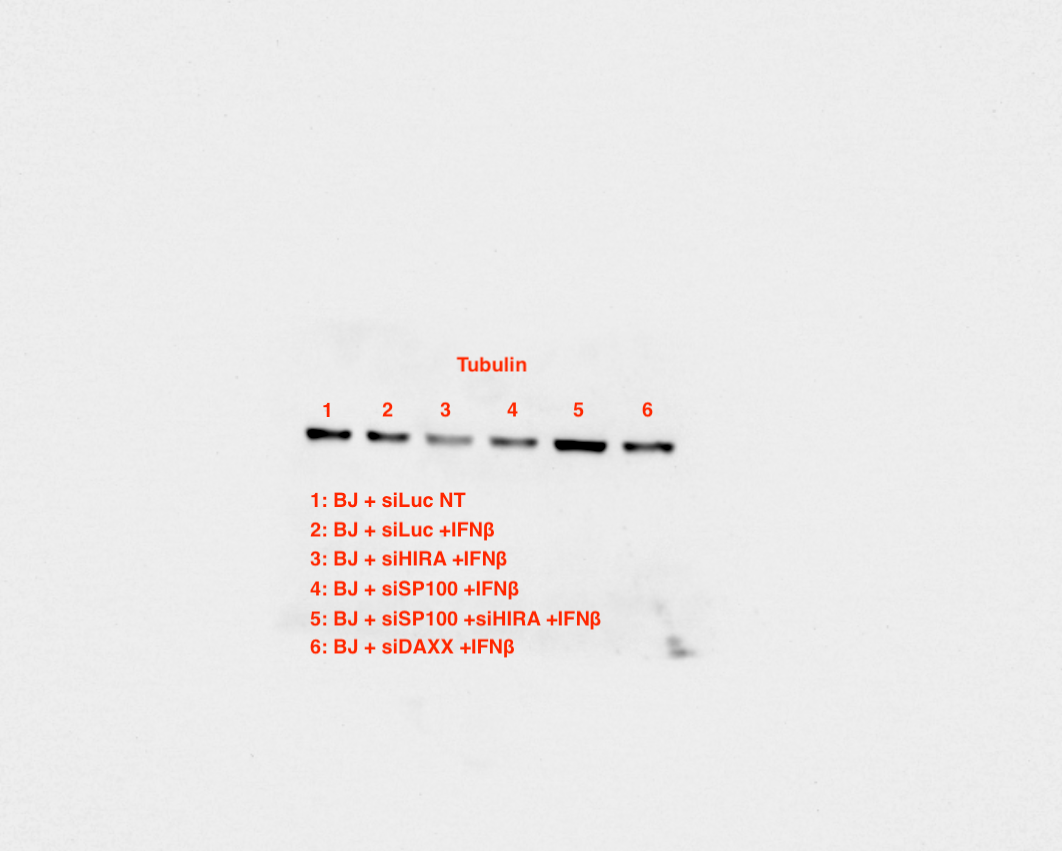

Supplement: Figure 7—source data 6. [file elife-80156-fig7-data6.tif]
